# High-Contrast Fluorescence Imaging in Fixed and Living Cells Using Optimized Optical Switches

**DOI:** 10.1371/journal.pone.0064738

**Published:** 2013-06-05

**Authors:** Liangxing Wu, Yingrui Dai, Xiaoli Jiang, Chutima Petchprayoon, Jessie E. Lee, Tao Jiang, Yuling Yan, Gerard Marriott

**Affiliations:** 1 Department of Bioengineering, University of California, Berkeley, California, United States of America; 2 Department of Bioengineering, Santa Clara University, Santa Clara, California, United States of America; University of Illinois, Urbana-Champaign, United States of America

## Abstract

We present the design, synthesis and characterization of new functionalized fluorescent optical switches for rapid, all-visible light-mediated manipulation of fluorescence signals from labelled structures within living cells, and as probes for high-contrast optical lock-in detection (OLID) imaging microscopy. A triazole-substituted BIPS (TzBIPS) is identified from a rational synthetic design strategy that undergoes robust, rapid and reversible, visible light-driven transitions between a colorless spiro- (SP) and a far-red absorbing merocyanine (MC) state within living cells. The excited MC-state of TzBIPS may also decay to the MC-ground state emitting near infra-red fluorescence, which is used as a sensitive and quantitative read-out of the state of the optical switch in living cells. The SP to MC transition for a membrane-targeted TzBIPS probe (C_12_-TzBIPS) is triggered at 405 nm at an energy level compatible with studies in living cells, while the action spectrum of the reverse transition (MC to SP) has a maximum at 650 nm. The SP to MC transition is complete within the 790 ns pixel dwell time of the confocal microscope, while a single cycle of optical switching between the SP and MC states in a region of interest is complete within 8 ms (125 Hz) within living cells, the fastest rate attained for any optical switch probe in a biological sample. This property can be exploited for real-time correction of background signals in living cells. A reactive form of TzBIPS is linked to secondary antibodies and used, in conjunction with an enhanced scope-based analysis of the modulated MC-fluorescence in immuno-stained cells, for high-contrast immunofluorescence microscopic analysis of the actin cytoskeleton.

## Introduction

Photochemical manipulation of organic molecules has been used for reversible and irreversible control of the two states of photochromic molecules for more than a century [1]–[4], with recent attention shifting towards the design of both synthetic and genetically-encoded photochromes for applications in biology [2], [4]. For example, photochromes that exhibit fluorescence emission in only one of their two states are key to the success of super-resolution fluorescence microscopy [5]–[7], high-contrast fluorescence microscopy (optical lock-in detection, OLID) [8]–[10], and for optical control of protein activity and cellular processes in living systems [11]–[13]. The new optical switches introduced in this study are optimized for high-contrast imaging of ensemble populations of probe molecules in fixed and living cells.

**Figure 1:**
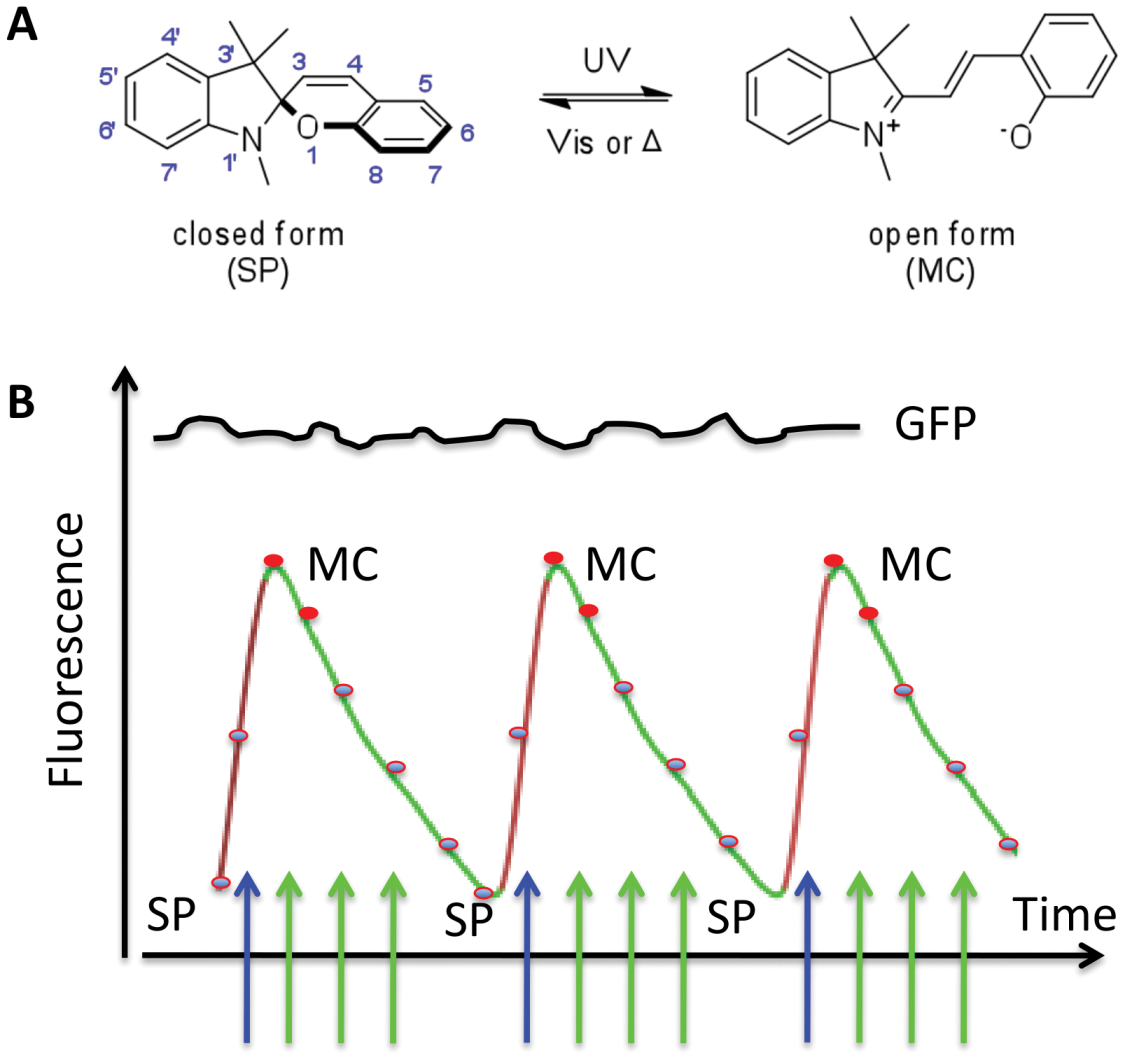
Schematic representations of optical switching reactions and the modulation of MC fluorescence. (**A**), Optically-induced transitions between the SP and MC states of BIPS. (**B**), Modulation of the MC-fluorescence signal in response to orthogonal control of the SP and MC states.

Benzospiropyran-derived photochromes, including 1′,3′-dihydro-1′,3′,3′-trimethyl-spiro[2H-1-benzopyran-2,2′-(2H)-indole] (BIPS) [14]–[17], have been used as optical switches for applications in living cells [8]–[10], [18]–[19]. BIPS undergoes rapid and reversible, high quantum yield transitions between a closed, colorless and hydrophobic spiropyran (SP), and an open, brightly-colored and polar merocyanine (MC), as shown in Figure 1A. Exposure of the MC-state to visible light results in formation of the SP-state, or decay of the MC-excited state to the same MC-ground state, with emission of red fluorescence (Figure 1A) [8]–[10]. The MC-fluorescence while necessarily low because of the competing MC to SP transition, is extremely useful for studies in cells and tissue, as it provides sensitive, quantitative, dynamic and high-spatial resolution read-outs of the two states of the optical switch in the sample [2], [6], currently not possible using azobenzene-derived photochromes [4], [11]. Moreover, since the excited-state transitions between the SP and MC states proceed with defined quantum yields, exposure of a BIPS probe such as NitroBIPS to a defined, alternating sequence of near-UV and visible light results in a time-dependent change in the populations of the two states of the switch, manifest in an intensity waveform of MC-fluorescence [8], as schematized in Figure 1B. On the other hand, the corresponding fluorescence intensity from non-switchable fluorophores, or from background emission in the sample is more or less constant (Figure 1B). The unique properties of optical switch probes have been exploited in the new high-contrast imaging technique of optical lock-in detection (OLID) imaging microscopy [8]–[10]. The modulated fluorescence signal arising from control of a 2-state fluorescent photochrome, or photochromic FRET probe, is isolated from larger “DC”-like background signals in the sample using a digital lock-in detection approach and results in significant increases in signal contrast [8]–[10].

Unfortunately, most synthetic photochromes are poorly-suited for studies within living cells, as at least one of the two transitions is driven by exposing the cell to <365 nm light [18]–[20], which usually leads to a stress response [21]. While we were the first to show this effect can be minimized by using 2-photon excitation 720 nm to carry out the SP to MC transition in NitroBIPS [9], [12], an easier solution to reduce phototoxicity is to shift the action spectrum for the SP to MC transition to the red >400 nm (or >800 nm for 2-photon excitation) [22]. To this end, we detail a systematic chemical modification strategy in which small π–bonding groups are appended to different sites on the BIPS probe to red-shift the SP- and MC-absorption bands, and further characterized to identify probes that maintain optimal quantum yields for excited state transitions between the SP and MC states, and MC-fluorescence. We also demonstrate the new red-shifted NitroBIPS and their functionalized derivatives are well-suited for rapid optical switching of labeled structures within living cells. These enhancements including an improved analysis for OLID imaging are used as part of a new approach for high-contrast immunofluorescence imaging microscopy.

## Results

### Rationally-designed Red-shifted BIPS Probes

**Figure 2:**
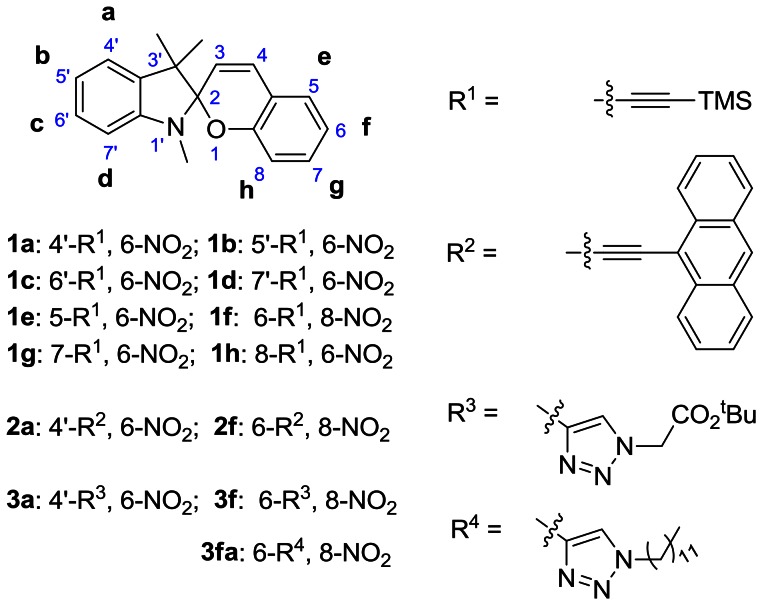
Summary of the structures of the BIPS derived probes prepared for the red-shifting of the SP and MC spectra.

The strategy to red-shift the SP-absorption spectrum of the BIPS photochrome involves extending π–bond through alkynyl substitution at each locus on the indoline and benzopyran sides of the BIPS molecule [1a–h; Figure 2]. Details of the synthesis and spectroscopic and photochemical characterization of all probes prepared for this study are provided in the (Information S1).

Analysis of the spectroscopic and photochemical data of the new BIPS probes allows us to evaluate the effect of introducing alkyne groups at defined sites on the BIPS molecule on specific spectroscopic and photochemical properties of the SP- and MC-states, the results of which are summarized in Table 1. In general, the absorption spectrum of the SP-state of alkynyl-substituted BIPS is more red-shifted when on the benzopyran side compared to the indoline side, while the extinction coefficient at the maximum SP-absorption wavelength is higher for substitutions made on the indoline side compared to the benzopyran side (Table 1). The SP-state of alkyne substituted BIPS probes (1a–1h) exhibit an intense red coloration upon a brief exposure to 365 nm or 405 nm, followed by a slower thermally-driven (dark reaction) return to the colorless SP-state. The half-life for the thermally-driven MC to SP transition is on the order of 100 seconds in ethanol. This time constant decreases for alkyne substitution made on the indoline side, and increases for alkyne substitutions on the benzopyran side of BIPS. BIPS probes having alkyne groups on the indoline side (1a–1d) have their maximum MC-fluorescence wavelength red-shifted by 5–10 nm compared to 6-NO_2_-BIPS, while substitutions made on the benzopyran side shift the MC-fluorescence maximum to 650 nm and 680 nm (compounds 1h and 1f respectively). A complete analysis of the spectroscopic and photochromic properties of all alkynyl substituted BIPS identified compounds 1a and 1f (Figure 2), as the most promising candidates for further study. In any case, the alkyne substituted NitroBIPS were converted to their respective anthracene and triazole derivatives in an effort to further extend the absorption maximum of the SP-state without compromising their ability to undergo reversible optical switching and to emit MC-fluorescence.

**Table 1:** Spectroscopic properties of BIPS probes in ethanol.

	λ_max, SP_ (nm)	ε_max, SP_ (M^−1^ cm^−1^)	ε_405__nm, SP_ (M^−1^ cm^−1^)	λ_max, MC_ (nm)	A_0_ e (30 s_365 nm)	A_0_ f (60 s_405 nm)	t_1/2_ (s)
A^a^	336	9600	300	539	0.400	0.019	1386
1a	329	12100	300	545	0.409	0.025	359
1b	336	10600	300	559	0.272	0.009	82
1c	322	14500	300	548	0.395	0.017	127
1d	332	17200	300	557	0.257	0.014	51
1e^b^	331	8500	700	N/A	N/A	N/A	N/A
1f	357	3600	1300	578	0.173	0.067	Stable^d^
1g^b^	336	6800	900	N/A	N/A	N/A	N/A
1h	337	9300	700	550	0.392	0.028	5715
2a	398	15500	14500	548	0.382	0.162	273
2f^c^	423	20900	21600	599	0.022	0.106	N/A^c^
3a	334	8800	200	538	0.342	0.026	821
3f	352	3100	1300	585	0.112	0.091	830

a Reference compound 6-NO_2_-BIPS;

b Photochemistry of **1e** and **1g** is too low to be measured;

c The MC-state of **2f** does not convert back to the SP-state even with light irradiation;

d The MC-state of **1f** is thermally stable in the dark at room temperature but it converts back to SP state upon exposure to green light;

e Colorability of the probe after exposure to 365 nm light for 30 s;

f Colorability of the probe after exposure to 405 nm light for 60 s.

### Triazole and Anthracene Derivatives of BIPS

**Figure 3:**
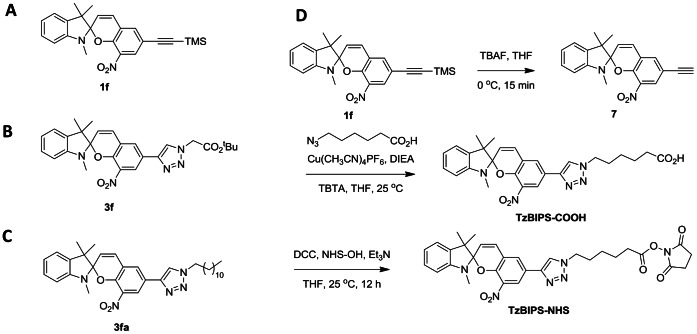
Specific examples of BIPS derived probes used in this study. **A**), 6-substituted TMS-alkynyl-BIPS (compound 1f); **B**), TzBIPS, Triazole-substituted BIPS (compound 3f); **C**), C_12_-TzBIPS (compound 3fa); **D**), Synthetic scheme used to prepare triazole substituted BIPS, and the N-hydroxysuccinimide ester derivative (NHS-TzBIPS).

**Figure 4:**
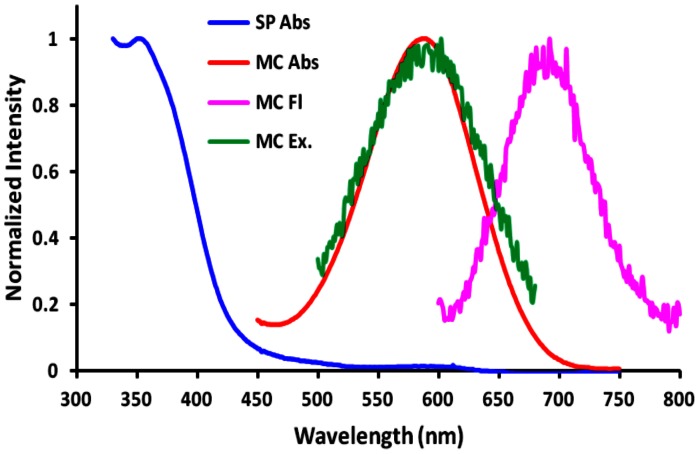
Normalized absorption and fluorescence of TzBIPS (3f) in ethanol: SP absorbance (blue); MC absorbance (red); MC excitation spectrum (green) and MC fluorescence (pink). Spectra are recorded for the probe dissolved to 30 µM in ethanol.

The alkynyl substituted BIPS probes are readily converted to their corresponding anthracenes, and triazole derivatives. The spectral and photochromic properties of these molecules are summarized in Table 1. Although the SP-states of the anthracene-substituted probes are significantly red shifted compared to alkynyl-BIPS (1a and 1f), only compound 2a exhibits reversible optical switching between the SP and MC states in solution or in living cells. The triazole-substituted BIPS probe, 3f (TzBIPS; Figure 3B) has the most red-shifted SP- and MC-absorption and excitation spectra, and MC-emission spectrum of all the triazole substituted probes (Figure 4). TzBIPS also exhibits robust and efficient orthogonal optical switching between its SP and MC states following alternate exposure to 405 nm (SP to MC) and >550 nm (MC to SP). The quantum yields for transitions between the SP and MC states are notoriously difficult to measure, with the best estimates given as 0.1 [23]. We also note that the quantum yield for MC-fluorescence must be, as we have previously noted [8], necessarily low because of the competing decay of the MC-state from the MC to SP transition. The capacity of the SP-state of BIPS to form the MC-state is quantified by the colorability, which is defined as the absorbance A_0_ of the solution at the maximum MC-absorption wavelength under a defined irradiation condition (see footnote to table 1). TzBIPS has the highest colorability of all probes prepared for this study with the value at 0.091 (Table 1). Based on these superior properties we selected TzBIPS (3f) and its derivatives for imaging applications within living cells (Information S1).

### High-fidelity Optical Switching of TzBIPS in Living Cells

**Figure 5:**
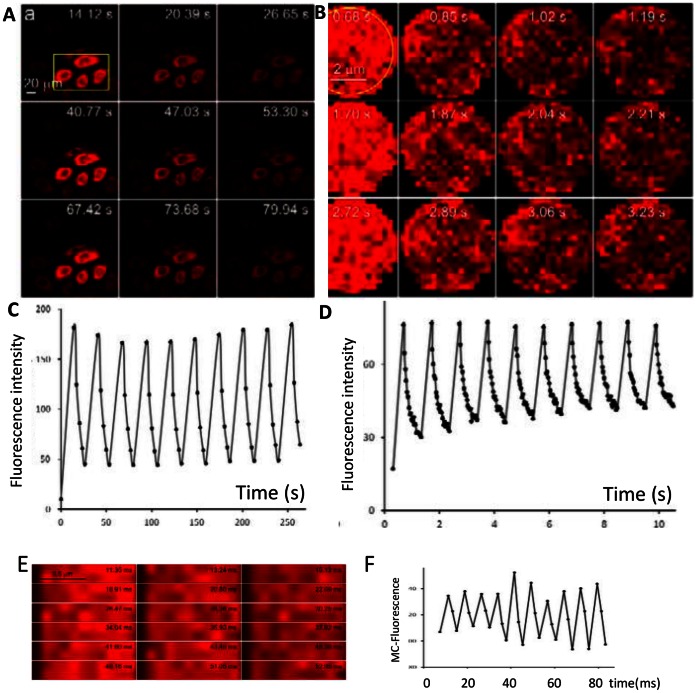
Optical switching of C_12_-TzBIPS (Fig. 3C) in living NBT-II cells. **A**), Image montage showing MC fluorescence signal of MC-state of C_12_-TzBIPS within a field of living cells over 3 cycles of optical switching using a low power objective; **B**), Higher magnification view of the optical switching of MC-fluorescence of C_12_-TzBIPS within a single cell in the same sample; **C**), Intensity trace of MC-fluorescence corresponding to the yellow boxed region in (5A); **D**), Intensity trace of MC-fluorescence within the yellow boxed region in (5B). **E**), Image montage 6 cycles of rapid optical switching of C_12_-TzBIPS in living cells within a narrow field of view; **F**), Intensity trace of the MC-fluorescence averaged over the entire image field (**E**).

First, we set about studying the optical switching properties of a cell permeable, and membrane-targeting version of TzBIPS, named C_12_-TzBIPS (Figure 3C). A single cycle of optical switching between the SP- and MC-states of the C_12_-TzBIPS in living NBT-II cells (obtained from ATTC) is achieved by first irradiating the yellow-boxed area (Figure 5A) in the field with 5 scans at 405 nm, which effectively triggers the SP to MC transition. The image montage shown in figure 5A shows that the MC-fluorescence is generated from the SP-state only in the region exposed to 405 nm. The entire image field is then exposed to multiple scans at 555 nm, which progressively decreases the MC fluorescence signal in the yellow-boxed region to a very low level (Figure 5C). This reduction in the intensity of the MC-signal is the result of the progressive formation of SP from the 555 nm excited MC molecules, whereas no MC-fluorescence signal change outside of this region. A relatively low power of 555 nm light is sufficient to bring about the MC to SP transition for cells shown in figure 5A (4.5% of the maximum laser output) and is not accompanied by any significant photobleaching of MC, as seen by the constancy of the maximum MC-signal over the 10 cycles (Figure 5C). Subsequent exposure of the yellow-boxed region of the same field to 405 nm results in a repopulation of the MC-state while an additional exposure of the entire field to 555 nm yet again decreases the intensity of the red MC-fluorescence signal. Multiple cycles of optical switching between the SP and MC states of C_12_-TzBIPS can be brought about in living cells and quantified through measurements of the MC fluorescence signal (Figure 5C). Optical switching between the SP and MC states of C_12_-TzBIPS in living cells is robust, with little change in the maximum intensity of the MC-fluorescence over the 10 cycles shown in Figure 5C. Moreover, the SP to MC transition is brought about for C_12_-TzBIPS within living cells using only 10% of the maximum output of the 5 mW laser, and cells are only exposed to 405 nm for 10% of the duty cycle. Modest exposure of the cell to 405 nm light was not accompanied by any noticeable effect on the cell morphology or other indicator evidence of phototoxic effects, for example blebbing, cell-rounding and presence of retraction fibers. In fact the exposure level to 405 nm light is far less than that used to excite CFP fusion proteins, and is comparable to that used to control Lov2-Rac1 in cells [13].

A much faster rate of optical switching of the MC-fluorescence of C_12_-TzBIPS is realized by reducing the region over which the 405 nm and 555 nm lasers are scanned. NBT-II cells from the same sample used above were selected for a rapid optical switching study (Figure 5 B, D). In particular, a 5 µm×5 µm region within a single cell is exposed to 10 sequential scans of 405 nm at 10% of the maximum laser power. This perturbation generates the MC-state within the selected field and is followed by carrying out 20 sequential scans of the same field at 555 nm (10% of the maximum power), with a pixel dwell time of 1.58 µs. A montage of MC-fluorescence images in the 5 µm × 5 µm region is shown for the first 3 cycles of optical switching (Figure 5B), with a plot of the MC-intensity profile for 10 cycles shown in Figure 5D. The time to complete a single cycle of optical switching is now reduced to ∼1 second again with little evidence of fatigue or photobleaching over the 10 cycles (Figure 5D).

#### Rapid optical switching of MC-fluorescence in living cells

Manipulating the SP and MC states of C_12_-TzBIPS in a smaller region of interest is used to increase the rate of optical switching within labeled cells [13]. Thus by increasing the 555 nm laser power to 20% and reducing the pixel dwell time to 0.79 µs, each image in the field is acquired within 1.89 ms, and a single cycle of optical switching is complete within 7.56 ms. An image montage of MC-fluorescence is shown for 6 cycles of optical switching in Figure 5E, and an intensity trace of MC fluorescence over 10 cycles shown in Figure 5F. As expected, the very narrow dimension of the selected region accentuates the mobility of vesicles, and precludes tracking of individual vesicles between successive frames. Nonetheless, it is evident from the average MC-intensity over the entire field that the MC-fluorescence is modulated within a few milliseconds as a result of rapid transitions between the SP and MC (Figure 5F). Significantly, this demonstration shows that it is now possible to carry out a 4-cycle OLID imaging analysis of a TzBIPS probe within a cell at the video rate.

### OLID Immunofluorescence Microscopy

An N-hydroxysuccinimide ester derivative of TzBIPS (Figure 3D) is used to prepare covalent antibody conjugates, which are used as part of an OLID-based approach to improve image contrast in immunofluorescence microscopy. NIH 3T3 cells are fixed using a methanol fixing procedure, and labeled with a polyclonal anti-actin antibody raised in rabbit. After washing off unbound rabbit anti-actin antibody, the cells are treated with the TzBIPS-conjugated goat anti-rabbit antibody. The cells were imaged by confocal microscopy by using a Zeiss LSM 700 microscope [10]. Images of MC-fluorescence are recorded for the same image field throughout the course of optical switching brought about exposing the TzBIPS-labelled preparation to multiple cycles of optical perturbation that includes two scans at 405 nm (15% of 5 mW) immediately followed by 10 scans of the same field at 555 nm (55% of 10 mW).

### Enhanced Lock-in Detection Approach to Analyze Modulated Fluorescence Signals

An improved method for optical lock-in detection (OLID) fluorescence microscopy is employed in this study to generate high-contrast images of the modulated MC-emission from TzBIPS labeled actin filaments in cells. In the “scope” approach to OLID imaging [24], we exploit our earlier finding that the 405 nm-mediated change in the fluorescence signal from the MC-state is a sufficiently sensitive indicator of optical switching of the TzBIPS probe in a pixel, and is easily discriminated when analyzed over multiple cycles of optical switching compared to the corresponding response of the signal from background probes and noise. We have introduced both the scope and scope-weighted correlation measures, which are calculated on a pixel-by-pixel basis as described in our previous work [24]. In brief, the scope values are calculated on a pixel-by-pixel basis as follows:
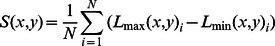



Where, *N is* the number of cycles used to calculate the scope value, *L*_max_ (x,y)*_i_* and *L*_min_ (x,y)*_i_* are local maximum and minimum intensity respectively for the n^th^ switching cycle, and (x, y) represents the pixel location. The scope is an average measure of the intensity modulation for the pixel location within a switching cycle. It is computed for each pixel in the image over several cycles of optical switching. For the images detailed in this study, where the noise is very strong and the signal from MC-emission is weak, we introduce a modified scope-based approach, in which a weighted intensity image is constructed using the scope measures as the weights.

#### OLID analysis of actin in immuno-stained cells

**Figure 6:**
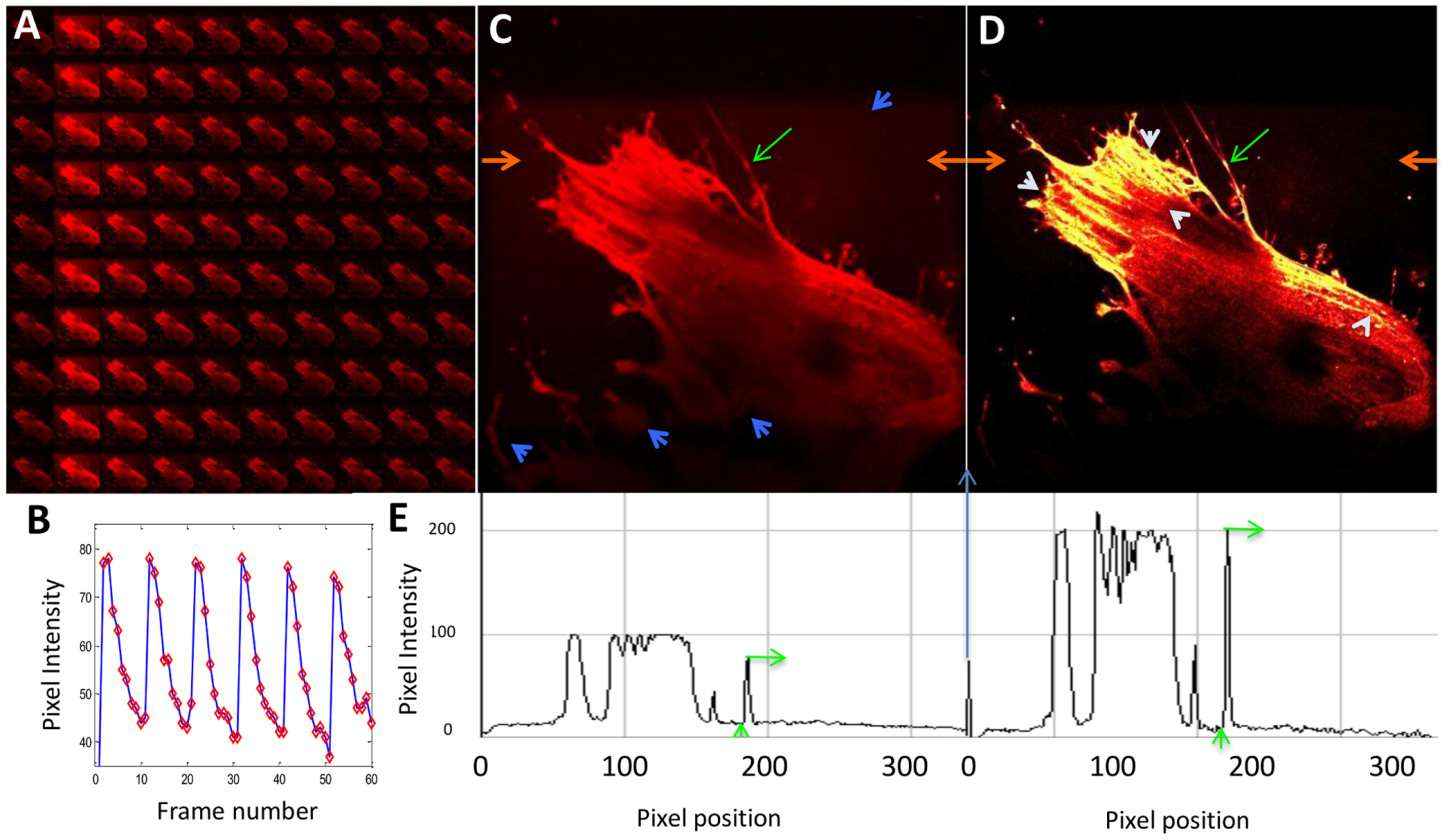
OLID analysis of actin cytoskeleton in NIH 3T3 cells as seen through the MC fluorescence of a TzBIPS labelled second antibody. Goat anti-rabbit antibodies labelled with TzBIPS are used to label an rabbit anti-actin antibody previously added to fixed NIH 3T3 cells. **A**), a montage of MC-fluorescence images over 10 cycles of optical switching within a the immunostained sample harboring a diffuse background signal. The montage suggests that little to no bleaching or fatigue of the TzBIPS probe, which is confirmed in a trace of red fluorescence in a highly modulated region of the image field seen over the ten cycles of optical switching (**B**). trace of MC-fluorescence signal in a stress fiber over the course of optical switching; (**C**), image of the red-fluorescence in the sample. Blue arrows reveal loci of background signals that are not visible in the corrleation image. (**D**), corresponding image of scope-weighted intensity. White arrows indicate regions of the actin cytoskeleton that are well-resolved in the scope-weighted intensity image but not in the red fluorescence intensity image. (**E**), Pixel intensity profile as a function of pixel position along a line indicated by the orange arrows in figure 6 **C,D**. The intensities of the actin-rich structure (indicated by the green arrow in figures 6 **C,D**) and its immediate background are indicated by the green arrows in figure 6 **E** and used to calculate the signal/background ratio.

NIH 3T3 cells are labelled with antibodies against actin and a TzBIPS labeled secondary antibody with a diffuse background fluorescence introduced during chemical fixation. The sample is scanned at 405 nm, which brings about an immediate increase in red MC-fluorescence that is visible upon subsequent excitation of the field to 555 nm. Further scanning of the field with 555 nm reduces the intensity of MC-fluorescence, as seen in the montage of the images of the field recorded over 10 cycles of optical switching (Figure 6A). The MC-fluorescence signal exhibits an identical intensity profile for each cycle of optical switching, and a variance of ∼10% in the maximum signal (Figure 6B). Significantly, the image montage and MC-intensity trace show that TzBIPS experiences little in the way of fatigue or bleaching of MC-fluorescence over the 10 cycles of optical switching (Figure 6A, B). The red fluorescence intensity image of these cells (Figure 6C) recorded immediately following exposure of the cells to 405 nm reveals the presence of actin filaments in stress fibers, cell-surface attachments and at the cell cortex, membrane labeled structures and a diffuse signal throughout the cell that obscures detail of individual actin structures and illuminates other regions indicated with blue arrows that may arise from background introduced during chemical fixation or MC (actin filaments) fluorescence (Figure 6C). The scope measure is computed for every pixel in the image field over the course of the 10 cycles of optical switching, and used to generate the scope-weighted intensity image.

Moreover, since the TzBIPS-labelled second antibodies are completely immobilized (by their chemical fixation) over the course of the 10 cycles of optical switching, the value of scope is high and largely uniform within MC-rich regions. Image contrast may be enhanced then by using the scope measures as weights in a scope-weighted intensity image (Figure 6D). This contrast-enhanced image is realized by multiplying the scope measures at each pixel by the corresponding intensity of the fluorescence image (from Figure 6C). The contrast in the scope-weighted image, shown in Figure 6D, is significantly improved compared to the intensity image (Figure 6C), revealing individual bundles of actin filaments at actin stress fibers close to the leading edge of the cell and fine details of cortical actin structures (indicated in white arrows) that are obscured by the background emission in the sample (indicated by blue arrows in Figure 6C). Also apparent in the scope-weighted image (Figure 6D) is the higher contrast in regions away from the cell, the intensity signal of which is evidently dominated by background signals where the corresponding scope value is close to zero, and thus the weighted intensity is almost zero.

Clearly, the scope-weighted image (Figure 6D) leads to a significant improvement in the contrast and noise reduction compared to the original image (Figure 6C). Quantitative measures of the improvement in image contrast between the original and the scope-weighted intensity images (Figure 6C,D) are derived from analysis of the fluorescence intensity profile for a line drawn on the same path for both images (indicated by red arrows in Figures 6C and 6D). The pixel intensity as a function of pixel position is reproduced respectively for both images as shown in Figure 6E. The green arrows in the plot indicate the intensities for an actin rich structure and its adjacent background indicated by green arrows in Figures 6 C,D. The ratio of these intensities provides a measure of the signal to noise in each image as 4.85 and 20.92 respectively, corresponding to an improvement in contrast of a factor of 4.31. Ordinarily, the intensity image of the immune-stained cells reported in Figure 6C would be the one used by an investigator, yet our scope-based OLID approach can be used to improve the signal to background ratio for the image by more than 4-fold. The improvement in image contrast arises largely from the selective amplification of the modulated MC fluorescence signal from the non-switching background fluorescence. In the example shown in Figure 6, the background signal is relatively low and arises from extraneous red emitting molecules created or added to the preparation during the chemical fixation and washing steps. Even greater improvements in image contrast then can be expected in immune-stained samples harboring much higher level of background, such as those derived from thick slices of tissue and intrinsically fluorescent samples such as algae and plant cells. Finally, further improvements in image contrast for samples containing higher background signals than those present in Figure 6 are possible by combining the scope-weighted intensity (used in this study) and the correlation coefficient [8], [24]. Finally, while the focus of our studies using TzBIPS has been on imaging of ensemble populations of labeled proteins and structures in fixed and living cells, it is clear that the optimized optical switching and MC-fluorescence properties of TzBIPS will also find use as probes for ensemble and single molecule imaging beyond the diffraction limit [7], [25]–[27].

### Summary

We have detailed the rational design, synthesis and characterization of new fluorescent optical switches based on the BIPS photochrome for all-visible light driven control of MC-fluorescence in living systems. These spectroscopic and photochemical analyses are used to identify a new class of BIPS photochrome, called TzBIPS whose SP and MC absorption spectra, and MC-fluorescence are the most red-shifted of all known BIPS derived photochromes [3], [18]–[20]. A membrane-directed C_12_-TzBIPS probe is shown to undergo rapid and reversible, high-fidelity optical switching between the SP and MC states in response to alternate exposure of the cell to low powers of 405 nm and 555 nm lasers. Optical perturbations carried out over 10 or more cycles of optical switching do not cause any noticeable fatigue or bleaching or stress-like effects on the cell. The progress of optical switching is quantified in these living cells by recording images of the 555 nm induced MC-fluorescence. The 405 nm triggered SP to MC transition of C_12_-TzBIPS is complete within the 790 ns pixel dwell time of the confocal microscope, which suggests that the photochemistry reaction underlying the SP to MC transition is most certainly faster, and is not a time-limiting factor for optical switching. A single cycle of optical switching of C_12_-TzBIPS in a region of interest is realized within 8 ms (125 Hz) within living cells, the fastest of any reported BIPS probe. This property allows the background fluorescence in a sample to be corrected (subtracted by subtracting the post from the pre-405 nm exposed MC-fluorescence images) much faster than the video-rate, a unique capability for probes used within living cells. We note that the rate of optical switching of MC-fluorescence could be improved even further by decreasing the time required to switch between scanning the field with the 405 nm and 555 nm lasers. Finally we introduce an amino-reactive form of TzBIPS for covalent labeling of biomolecules, including secondary antibodies that are used in this study as part of an approach for high-contrast IF imaging microscopy. The scope-weighted intensity analysis is particularly well-suited for high-contrast imaging of chemically-fixed TzBIPS probes, and can realize improvements in the signal to background ratio of >4-fold improvement, even for samples that are otherwise considered background free. The new TzBIPS labeled antibody simplifies the design of antibody probes for OLID-IF compared to FRET-based antibody probes [10], [28], and will be useful for IF preparations containing high levels of intrinsic fluorescence, and for the imaging of labeled cells within thick tissue.

## Supporting Information

Information S1 **Details of the synthesis, spectroscopic and photochemical characterization of all the probes.**
(PDF)
