# Supplementary material for: High-Contrast Fluorescence Imaging in Fixed and Living Cells Using Optimized Optical Switches
Source: PLoS One. 2013 Jun 5;8(6):e64738. doi: 10.1371/journal.pone.0064738 (PMC3674008; doi:10.1371/journal.pone.0064738)
Supplement: Information S1 — Details of the synthesis, spectroscopic and photochemical characterization of all the probes. (PDF) [file pone.0064738.s001.pdf]

Supporting Information

High-contrast Fluorescence Imaging in Fixed and Living Cells Using Optimized Optical Switches

Liangxing Wu, Yingrui Dai, Xiaoli Jiang, Chutima Petchprayoon, Jessie Eileen Lee, Tao Jiang, Yuling Yan and Gerard Marriott

Department of Bioengineering, University of California-Berkeley, Berkeley, CA 94720

Email: marriott1@berkeley.edu

TABLE OF CONTENTS

General Experimental Methods S2

Design and Synthesis of Compounds 1-5 S2-S20

Spectroscopic and Photochromic Properties S21-S27

Copies of ^{1}H, ^{13}C NMR and MS Spectra S28-S49

General Experimental Methods

All reactions were carried out under an atmosphere of dry nitrogen. Glassware were oven-dried prior to use. Unless otherwise indicated, common reagents or materials were obtained from commercial source and used without further purification. Dry distilled THF and CH_2Cl_2 were obtained from Acros and used as received. Flash column chromatography was performed using silica gel 60 (70-230 mesh). Analytical thin layer chromatography (TLC) was carried out on Merck silica gel plates with QF-254 indicator and visualized by UV. Absorbance spectra were recorded on a Shimadzu UV-1601PC spectrophotometer at room temperature. Fluorescence spectra were obtained on a AMINCO-Bowman Series 2 spectrofluorometer with 4 nm excitation and emission slit width at room temperature.

^{1}H and ^{13}C NMR spectra were recorded on a Bruker 400 (400 MHz ^{1}H; 100 MHz ^{13}C) spectrometer at room temperature. Chemical shifts were reported in ppm relative to the residual solvent signal ($CDCl_3$: 99.8 % D contains 0.05% v/v TMS, δ 7.26 ppm ^{1}H; δ 77.00 ppm ^{13}C).

Photochemistry: a hand-held UV lamp (UVGL-25) was used as the 365 nm light source. A laser pointer (GLP-405nm) http://www.green-blue-laser.com/id126.html was used as the 405 nm light source. An OptoLED light from CAIRN research http://www.cairn-research.co.uk/catalogue/illumination/optoled/product/optoled-light-source was used as the light source for 530 nm.

Design of red-shifted BIPS derivatives

There is increasing scepticism amongst biologists about using near ultraviolet light to generate fluorescent signals from a labeled cell. The main issues underlying this trend are: living cells undergo a UV-triggered stress response that can interfere with the study at hand and, most commercial confocal fluorescence microscopes do not allow for laser or wde-field excitation of the field with light lower than 400 nm. The challenge in exploiting unique properties of optical switch probes in cell and tissue biology then is to shift the SP-absorption band beyond 400 nm, while maintaining

robust and high-fidelity switching between the SP and MC states.

Our strategy in red-shifting the SP-absorption band from a typical valiue of 350 nm to beyond 400 nm is to maintain the core aromatic structure of BIPS while extending π-bond conjugation at different sites on the aromatic scaffold. We reasoned that this strategy would lead to a red-shifting of the SP-absorption band while providing a quantitative evaluation and insight into how these spectral shifts affect quantum yields for excite state transitions between SP and MC. The alkynyl substituent is chosen to extend the conjugation because (i) it is compact, engages in π-bonding with the indoline and pyran moeities and only modestly increases the mass of BIPS; (ii) the alkyne group is easily introduced into BIPS by using a palladium catalyzed *Sonogashira* coupling reaction; (iii) red-shifting of the absorption wavelength of aromatic probes with alkynyl groups has already been demonstrated for BODIPY; (iv) alkynyl groups are readily functionalized *eg*. by direct attachment to an aryl moiety or by conversion to a triazole via click chemistry. Here we describe the synthesis of BIPS molecules harboring alknyl groups at different sites and carry out a systematic investigation of the effect of the alkyne substituent on the spectroscopic and photochromic properties for each substitution. Alkyne substitutions are carried out using the 6-NitroBIPS with the exception of 8-nitro-BIPS (compound **1f**).

Several alkynyl BIPS with promising photochromic properties are chosen for further functionalization with anthracene or triazole in order to improve their photoswitching performance. In particular, 9-Alkynyl-anthracene has a strong absorbtion band at 405 nm and is easily prepared from the alkynyl-BIPS while the triazole group will also increase π-conjugation and is readily generated from the alkyne by using click chemistry.

Synthesis of BIPS derivatives

The synthesis of new BIPS probes is summarized in Scheme S1-2 and detailed in the experimental procedures. The alkyne substituted BIPS **1a-1h** are prepared from the corresponding Iodo-BIPS precursors **4a-4h** (bromo-BIPS for **4f**) by a *Sonogashira*

coupling reaction. The iodo-BIPS are synthesized by condensation of corresponding indoline and salicylaldehyde. The aldehyde fragment for BIPS **4a-4d** is a commercially available compound 2-hydroxy-5-nitrobenzaldehyde. The indoline fragments for **4a-4d** are synthesized from the corresponding Iodo-anilines using reported conditions. It is noteworthy that the two indolines **5a** and **5c**, which can be separated by chromatography, are generated from 3-iodoaniline. For **4e-4h**, we note that the indoline fragment is commercially available and the substituted salicylaldehydes are prepared according to reported procedures. Unfortunately, several reactions conditions employed in the preparation of 5-iodo-3-nitrosalicylaldehyde all lead to the formation of inseparable mixtures.

a

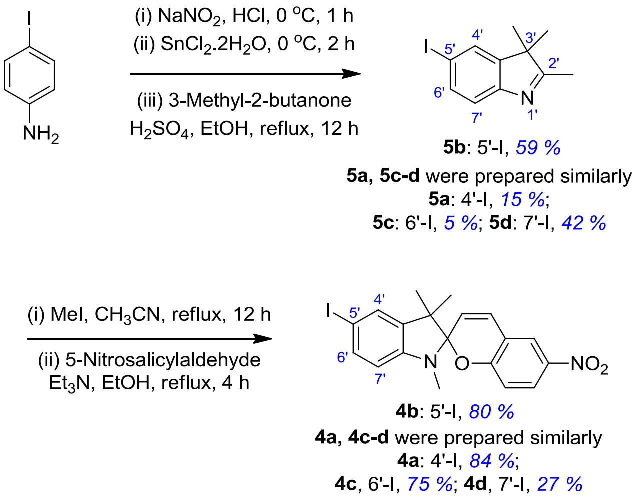

b

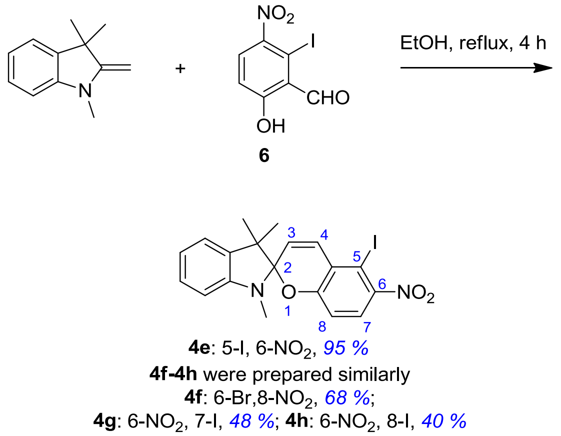

Scheme S1. Illustrative synthesis of (a) **4a** - **4d**; (b) **4e** - **4h**.

Commercial 5-bromo-3-nitrosalicylaldehyde is used instead to afford the 6-bromo-8-Nitro-BIPS, **4f**. BIPS derivatives **4a-4h** are generated in good yield through reaction of indolines with salicylaldehydes in boiling ethanol.

a

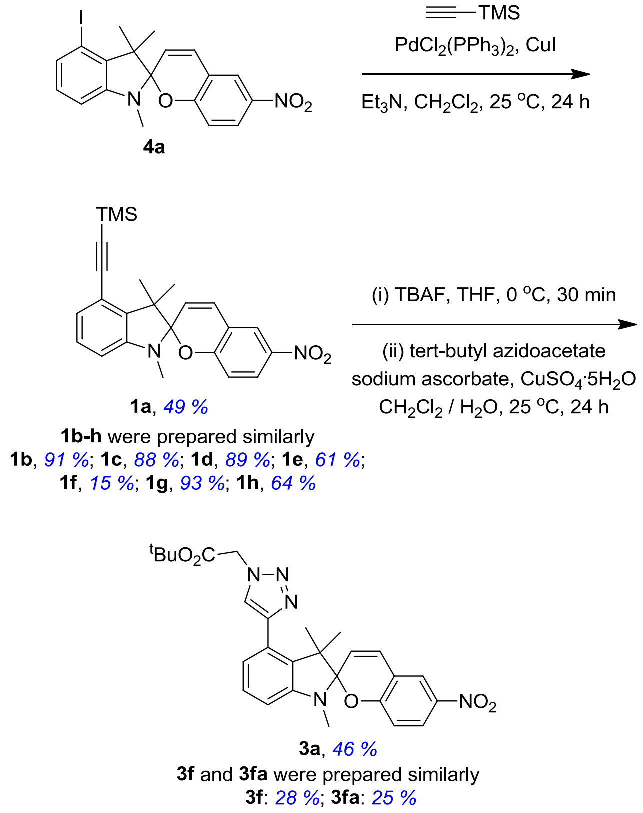

b

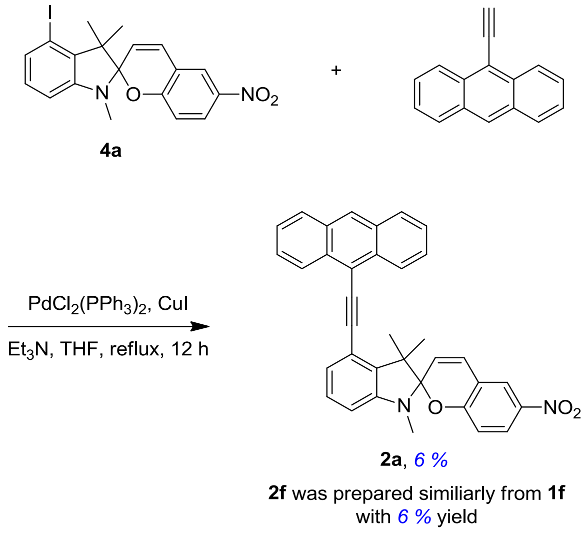

Scheme S2. Illustrative synthesis of compound (a) **1** and **3**; (b) **2**.

Compounds **4a-4h** are coupled with trimethylsilylacetylene to afford the alkynyl-BIPS **1a-1h**. The Sonogashira coupling is successful even at at 20c for iodo-BIPS. However, the reaction did not go to completion for the bromo-BIPS **4f** even after prolonged reaction time and elevated temperature although sufficient material is isolated for spectroscopic studies. The trimethylsilyl group in compounds **1a-h** is removed by tetrabutylammonium fluoride (TBAF) to generate the free acetylene derivative. Coupling of the deprotected compounds with 9-bromoanthracene was unsuccessful due to dimerization of the free acetylene BIPS. Thus compounds **2a** and **2f** are prepared from the treaction of the BIPS halide with 9-alkyne-anthracene using *Sonogashira* coupling. The low yield for these compounds is due to incomplete reactions and decomposition of the starting materials at high temperature.

Cu(I)-catalyzed cycloaddition of the free acetylene BIPS and tert-butyl azidoacetate afford the triazole substituted BIPS **3a** and **3f** in moderate yields. A derivative **3fa** with a long hydrocarbon chain for labeling of cell membranes is also prepared by a similar method.

A typical synthesis of compound 1

An oven-dried 100 mL Schlenk tube is charged with compound **4b** (103 mg, 0.23 mmol), $PdCl_2(PPh_3)_2$ (14 mg, 0.02 mmol) and CuI (4 mg, 0.02 mmol). The Schlenk tube is evacuated and back-filled with nitrogen. The process is repeated for three times. Dry dichloromethane (10 mL) is added via syringe to the Schlenk tube, followed by Et_3N (0.32 mL, 2.30 mmol) and trimethylsilyl acetylene (162 uL, 1.15 mmol). The reaction mixture is stirred at toom temperature for 24 h and then concentrated in vacuo. The residue is purified by flash chromatography (silica gel, 30 to 50 % CH_2Cl_2/Hexanes) to afford the pure product **1b** (88 mg, 91 %) as a yellow solid. ^{1}H NMR (400 MHz, $CDCl_3$) δ 8.04 – 7.99 (m, 2H), 7.35 (dd, *J* = 8.0, 1.5 Hz, 1H), 7.19 (d, *J* = 1.5 Hz, 1H), 6.93 (d, *J* = 10.3 Hz, 1H), 6.73 (d, *J* = 8.4 Hz, 1H), 6.46 (d, *J* = 8.0 Hz, 1H), 5.83 (d, *J* = 10.3 Hz, 1H), 2.75 (s, 3H), 1.28 (s, 3H), 1.16 (s, 3H), 0.25 (s, 9H); ^{13}C NMR (100 MHz, $CDCl_3$) δ 159.42, 147.91, 141.02, 136.13, 132.50, 128.44, 125.87, 125.36, 122.67, 121.05, 118.49, 115.39, 113.73, 106.68, 106.25, 106.08, 91.51, 52.02, 28.72, 25.64, 19.77, 0.12. HRMS (ESI) m/z calculated for

$C_{24}H_{27}N_2O_3Si$ ([M+H]$^+$) 419.1785; found 419.1789.

Compounds **1a**, **1c** – **1h** are synthesized using similar procedure as described for compound **1b**.

Compound 1a

The reaction did not go to completion even after long reaction times. Thus the reaction mixture is refluxed overnight and TLC showed complete conversion of the starting marterial **4a**.

Yellow solid (48 mg, 49 %). ^{1}H NMR (400 MHz, $CDCl_3$) δ 8.05 – 7.99 (m, 2H), 7.12 (t, *J* = 7.8 Hz, 1H), 6.96 – 6.92 (m, 2H), 6.76 (d, *J* = 8.7 Hz, 1H), 6.52 (d, *J* = 7.8 Hz, 1H), 5.83 (d, *J* = 10.3 Hz, 1H), 2.74 (s, 3H), 1.52 (s, 3H), 1.28 (s, 3H), 0.24 (s, 9H); ^{13}C NMR (100 MHz, $CDCl_3$) δ 159.82, 147.97, 140.90, 136.48, 128.48, 127.61, 125.88, 124.24, 122.67, 121.31, 118.50, 117.98, 115.37, 107.38, 106.33, 102.93, 98.47, 53.54, 28.90, 22.91, 19.38, -0.27. HRMS (ESI) m/z calculated for $C_{24}H_{27}N_2O_3Si$ ([M+H]$^+$) 419.1785; found 419.1788.

Compound 1c

Yellow solid (78 mg, 88 %). ^{1}H NMR (400 MHz, $CDCl_3$) δ 8.04 – 7.99 (m, 2H), 7.05 – 6.98 (m, 2H), 6.93 (d, *J* = 10.4 Hz, 1H), 6.73 (d, *J* = 8.4 Hz, 1H), 6.64 (s, 1H), 5.84 (d, *J* = 10.4 Hz, 1H), 2.73 (s, 3H), 1.27 (s, 3H), 1.17 (s, 3H), 0.25 (s, 9H); ^{13}C NMR (100 MHz, $CDCl_3$) δ 159.54, 147.53, 140.98, 137.09, 128.42, 125.85, 124.16, 122.66, 122.30, 121.34, 121.16, 118.54, 115.38, 110.12, 106.18, 105.83, 93.02, 52.20, 28.79, 25.59, 19.70, 0.00. HRMS (ESI) m/z calculated for $C_{24}H_{27}N_2O_3Si$ ([M+H]$^+$) 419.1785; found 419.1791.

Compound 1d

Yellow solid (84 mg, 89 %). ^{1}H NMR (400 MHz, $CDCl_3$) δ 8.05 – 7.99 (m, 2H), 7.24 (dd, *J* = 7.9, 1.2 Hz, 1H), 7.00 (dd, *J* = 7.2, 1.2 Hz, 1H), 6.94 (d, *J* = 10.4 Hz, 1H), 6.80 – 6.74 (m, 2H), 5.83 (d, *J* = 10.4 Hz, 1H), 3.23 (s, 3H), 1.27 (s, 3H), 1.14 (s, 3H), 0.21 (s, 9H); ^{13}C NMR (100 MHz, $CDCl_3$) δ 159.56, 147.60, 140.98, 137.15, 133.30, 128.48, 125.85, 122.66, 122.11, 121.34, 119.41, 118.42, 115.38, 106.48,

103.68, 103.20, 97.33, 50.98, 30.65, 25.88, 19.83, -0.31. HRMS (ESI) m/z calculated for $C_{24}H_{27}N_2O_3Si$ ($[M+H]^+$) 419.1785; found 419.1787.

Compound 1e

Yellow solid (63 mg, 61 %). ^1H NMR (400 MHz, $CDCl_3$) δ 7.89 (d, *J* = 9.1 Hz, 1H), 7.46 (d, *J* = 10.6 Hz, 1H), 7.21 (td, *J* = 7.7, 1.2 Hz, 1H), 7.10 (dd, *J* = 7.3, 0.8 Hz, 1H), 6.89 (td, *J* = 7.5, 0.8 Hz, 1H), 6.73 (d, *J* = 9.1 Hz, 1H), 6.57 (d, *J* = 7.7 Hz, 1H), 5.96 (d, *J* = 10.6 Hz, 1H), 2.74 (s, 3H), 1.29 (s, 3H), 1.21 (s, 3H), 0.35 (s, 9H); ^{13}C NMR (100 MHz, $CDCl_3$) δ 158.35, 147.64, 143.10, 136.02, 127.82, 127.05, 126.61, 122.49, 121.52, 121.14, 119.73, 116.18, 115.19, 109.16, 107.01, 105.89, 96.84, 52.27, 28.82, 25.80, 19.90, -0.30. HRMS (ESI) m/z calculated for $C_{24}H_{27}N_2O_3Si$ ($[M+H]^+$) 419.1785; found 419.1787.

Compound 1f

Prepared from compound **4f** (6-Br-8-NO_2-BIPS) by refluxing in THF overnight. The reaction did not go to completion even after elongated reaction time. The product is purified by preparative TLC plates (10 % EtOAc/Hexanes) to give a yellow solid (12 mg, 15 %).

^1H NMR (400 MHz, $CDCl_3$) δ 7.78 (d, *J* = 2.0 Hz, 1H), 7.35 (d, *J* = 2.0 Hz, 1H), 7.19 – 7.13 (m, 1H), 7.07 – 7.03 (m, 1H), 6.89 – 6.83 (m, 2H), 6.53 (d, *J* = 8.0 Hz, 1H), 5.88 (d, *J* = 10.4 Hz, 1H), 2.72 (s, 3H), 1.33 (s, 3H), 1.18 (s, 3H), 0.24 (s, 9H); HRMS (ESI) m/z calculated for $C_{24}H_{27}N_2O_3Si$ ($[M+H]^+$) 419.1785; found 419.1794.

Compound 1g

Yellow solid (97 mg, 93 %). ^1H NMR (400 MHz, $CDCl_3$) δ 7.90 (s, 1H), 7.21 (td, *J* = 7.7, 1.2 Hz, 1H), 7.12 – 7.07 (m, 1H), 6.97 (s, 1H), 6.94 – 6.87 (m, 2H), 6.56 (d, *J* = 7.7 Hz, 1H), 5.90 (d, *J* = 10.3 Hz, 1H), 2.74 (s, 3H), 1.31 (s, 3H), 1.20 (s, 3H), 0.26 (s, 9H); ^{13}C NMR (100 MHz, $CDCl_3$) δ 157.73, 147.56, 142.25, 135.95, 127.77, 123.66, 122.43, 121.43, 121.16, 120.25, 119.78, 118.87, 107.03, 106.24, 104.05, 99.94, 52.24, 28.79, 25.78, 19.86, -0.46. HRMS (ESI) m/z calculated for $C_{24}H_{27}N_2O_3Si$ ($[M+H]^+$) 419.1785; found 419.1787.

Compound 1h

Blue solid (66 mg, 64 %). ^{1}H NMR (400 MHz, $CDCl_3$) δ 8.11 (d, *J* = 2.7 Hz, 1H), 7.93 (d, *J* = 2.7 Hz, 1H), 7.15 (td, *J* = 7.7, 1.2 Hz, 1H), 7.06 (dd, *J* = 7.3, 0.8 Hz, 1H), 6.90 (d, *J* = 10.4 Hz, 1H), 6.85 (td, *J* = 7.5, 0.8 Hz, 1H), 6.55 (d, *J* = 7.7 Hz, 1H), 5.91 (d, *J* = 10.4 Hz, 1H), 2.72 (s, 3H), 1.31 (s, 3H), 1.20 (s, 3H), -0.07 (s, 9H); ^{13}C NMR (100 MHz, $CDCl_3$) δ 160.56, 147.65, 140.08, 136.13, 128.06, 127.92, 127.62, 122.06, 121.99, 121.29, 119.89, 118.64, 111.24, 107.30, 107.15, 101.92, 97.16, 52.15, 28.92, 25.51, 19.90, -0.68. HRMS (ESI) m/z calculated for $C_{24}H_{27}N_2O_3Si$ ($[M+H]^+$) 419.1785; found 419.1792.

Illustrative synthesis of compound 2

An oven-dried 100 mL Schlenk tube is charged with compound **4a** (134 mg, 0.23 mmol), 9-alkynylanthracene (250 mg, 1.20 mmol), $PdCl_2(PPh_3)_2$ (14 mg, 0.02 mmol) and CuI (4 mg, 0.02 mmol). The Schlenk tube is evacuated and back-filled with nitrogen. The process is repeated for three times. Dry THF (10 mL) is added via syringe to the Schlenk tube, followed by 1 mL of Et_3N. The reaction mixture is refluxed for 24 h and then concentrated in vacuo. The residue is passed through a short pad of silica gel eluted with 15 % EtOAc/Hexanes to afford the crude product which is further purified by preparative TLC plates (silica gel, 15 % EtOAc/Hexanes) to afford the pure product **2a** (10 mg, 6 %) as a yellow solid. ^{1}H NMR (400 MHz, $CDCl_3$) δ 8.62 (d, *J* = 8.5 Hz, 2H), 8.45 (s, 1H), 8.07 – 7.98 (m, 4H), 7.60 – 7.54 (m, 2H), 7.54 – 7.48 (m, 2H), 7.29 (d, *J* = 4.6 Hz, 2H), 6.96 (d, *J* = 10.4 Hz, 1H), 6.83 (d, *J* = 8.9 Hz, 1H), 6.66 – 6.59 (m, 1H), 5.88 (d, *J* = 10.4 Hz, 1H), 2.80 (s, 3H), 1.70 (s, 3H), 1.45 (s, 3H). HRMS (ESI) m/z calculated for $C_{35}H_{27}N_2O_3$ ($[M+H]^+$) 523.2016; found 523.2006.

Compound 2f

Green solid (10 mg, 6 %). ^{1}H NMR (400 MHz, $CDCl_3$) δ 8.58 (d, *J* = 8.7 Hz, 2H), 8.46 (s, 1H), 8.08 – 8.00 (m, 3H), 7.66 – 7.58 (m, 3H), 7.56 – 7.50 (m, 2H), 7.23 – 7.16 (m, 1H), 7.09 (d, *J* = 6.8 Hz, 1H), 7.00 (d, *J* = 10.4 Hz, 1H), 6.89 (t, *J* = 7.2 Hz, 1H), 6.57 (d, *J* = 8.0 Hz, 1H), 5.96 (d, *J* = 10.4 Hz, 1H), 2.79 (s, 3H), 1.41 (s, 3H), 1.23 (s, 3H). HRMS (ESI) m/z calculated for $C_{35}H_{27}N_2O_3$ ($[M+H]^+$) 523.2016; found

523.2011.

Illustrative synthesis of compound 3

A solution of compound **1a** (27 mg, 0.065 mmol) in 5 mL of dry THF is cooled to 0 ^{o}C and TBAF (1M in THF, 85 μL) is added dropwise via syringe. After stirring at 0 ^{o}C for 15 min, the reaction mixture is diluted with 20 mL of EtOAc and ished with water then brine. The organic layer is dried over Na_2SO_4 then concentrated under vacuum. The residue is dissolved in 5 mL of CH_2Cl_2 and azido tert-butylacetate (52 mg, 0.33 mmol) is added. Then 5 mL of water is added to the solution, followed by $CuSO_4{\cdot}5H_2O$ (2.5 mg, 0.01 mmol) and sodium ascorbate (2 mg, 0.01 mmol). The reaction mixture is stirred at room temperature overnight and diluted with 20 mL of CH_2Cl_2. The organic layer is ished with water, brine, dried over Na_2SO_4 and then concentrated under vacuum. The residue is purified by flash chromatography (silica gel, 20 to 40 % EtOAc/Hexanes) to afford the pure product **3a** (15 mg, 46 %) as a white solid. ^{1}H NMR (400 MHz, $CDCl_3$) δ 8.00 – 7.95 (m, 2H), 7.71 (s, 1H), 7.24 (t, *J* = 7.8 Hz, 1H), 6.92 (d, *J* = 10.4 Hz, 1H), 6.82 (dd, *J* = 7.7, 0.9 Hz, 1H), 6.74 – 6.70 (m, 1H), 6.62 (dd, *J* = 7.8, 0.7 Hz, 1H), 5.88 (d, *J* = 10.4 Hz, 1H), 5.11 (s, 2H), 2.77 (s, 3H), 1.52 (s, 3H), 1.47 (s, 9H), 0.98 (s, 3H); ^{13}C NMR (100 MHz, $CDCl_3$) δ 165.14, 159.98, 148.79, 147.27, 140.78, 133.99, 128.54, 127.67, 126.76, 125.76, 123.42, 122.67, 122.41, 121.56, 118.53, 115.31, 107.54, 107.30, 83.78, 53.78, 51.60, 29.34, 27.94, 24.84, 19.17. HRMS (ESI) m/z calculated for $C_{27}H_{30}N_5O_5$ ($[M+H]^+$) 504.2241; found 504.2247.

Compound 3f

Green solid (12 mg, 29 %). ^{1}H NMR (400 MHz, $CDCl_3$) δ 8.03 (d, *J* = 2.0 Hz, 1H), 7.92 (d, *J* = 2.4 Hz, 1H), 7.90 (s, 1H), 7.17 (td, *J* = 7.6, 1.2 Hz, 1H), 7.09 – 7.05 (m, 1H), 6.99 (d, *J* = 10.4 Hz, 1H), 6.89 – 6.84 (m, 1H), 6.54 (d, *J* = 8.0 Hz, 1H), 5.91 (d, *J* = 10.4 Hz 1H), 5.11 (s, 2H), 2.75 (s, 3H), 1.51 (s, 9H), 1.37 (s, 3H), 1.21 (s, 3H). ^{13}C NMR (100 MHz, $CDCl_3$) δ 165.09, 148.16, 147.40, 145.92, 137.05, 135.95, 128.41, 128.24, 127.68, 122.61, 122.25, 122.13, 122.10, 121.88, 121.39, 120.79, 119.84, 107.08, 106.97, 84.09, 52.19, 51.65, 29.69, 27.98, 26.03, 20.01. HRMS (ESI)

m/z calculated for $C_{27}H_{30}N_5O_5$ ($[M+H]^+$) 504.2241; found 504.2251.

Compound 3fa

Green solid (2 mg, 25 %). ^{1}H NMR (400 MHz, $CDCl_3$) δ 7.99 (d, *J* = 2.1 Hz, 1H), 7.93 (d, *J* = 2.1 Hz, 1H), 7.73 (s, 1H), 7.17 (td, *J* = 7.7, 1.2 Hz, 1H), 7.07 (d, *J* = 7.3 Hz, 1H), 6.99 (d, *J* = 10.4 Hz, 1H), 6.86 (t, *J* = 7.0 Hz, 1H), 6.54 (d, *J* = 7.6 Hz, 1H), 5.91 (d, *J* = 10.4 Hz, 1H), 4.40 (t, *J* = 7.2 Hz, 2H), 2.75 (s, 3H), 2.00 – 1.88 (m, 2H), 1.56 (s, 3H), 1.39 - 1.18 (m, 2H), 0.87 (t, *J* = 6.8 Hz, 3H). HRMS (ESI) m/z calculated for $C_{33}H_{44}N_5O_3$ ($[M+H]^+$) 558.3439; found 558.3430.

Synthesis of Compounds 5a-d

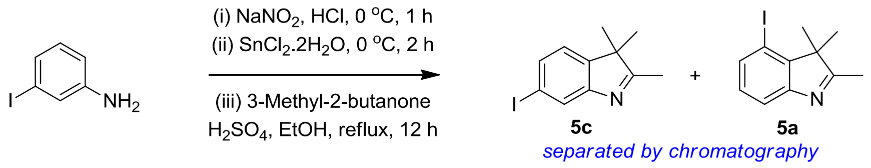

To a 100 mL flask were added 3-iodoaniline (2.5 g, 11.4 mmol) and concentrated hydrochloric acid (20 mL). The mixture was cooled to 0 ^{o}C in an ice bath. A solution of $NaNO_2$ (0.94 g, 13.7 mmol) in water (20 mL) was added dropwise to the aniline suspension. The mixture was stirred for 30 min at 0 ^{o}C and then a solution of $SnCl_2.2H_2O$ (7.7 g, 34.2 mmol) in concentrated hydrochloric acid (10 mL) was added dropwise. The reaction mixture was stirred at 0 ^{o}C for a further 2 h. The precipitate was filtered and dried. The solid was dissolved in 30 mL of EtOH then 3-methylbutanone (2.5 mL, 22.8 mmol) and concentrated H_2SO_4 (0.3 mL) were added. The reaction solution was refluxed for overnight and then cooled to room temperature. The volatile components were removed under vacuum and the residue was dissolved in EtOAc (100 mL) then washed with saturated Na_2CO_3 aqueous solution, water and brine. The organic layer was dried over Na_2SO_4 and evaporated under reduced pressure. The residue was purified by flash chromatography (10 % to 20 % EtOAc/Hexanes) to afford pure **5c** (143 mg, 4.4 %), pure **5a** (481 mg, 14.8 %) and 1.46 g mixture of **5a** and **5c**.

For **5a**: ^{1}H NMR (400 MHz, $CDCl_3$) δ 7.45 (dd, *J* = 7.9, 0.8 Hz, 1H), 7.39 (dd, *J* = 7.6, 0.8 Hz, 1H), 6.88 (t, *J* = 7.8 Hz, 1H), 2.14 (s, 3H), 1.28 (s, 6H).
For **5c**: ^{1}H NMR (400 MHz, $CDCl_3$) δ 7.83 (d, *J* = 1.5 Hz, 1H), 7.48 (dd, *J* = 7.7, 1.5 Hz, 1H), 6.99 (d, *J* = 7.7 Hz, 1H), 2.23 (s, 3H), 1.23 (s, 6H).

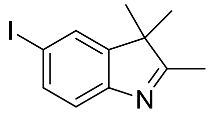

5b

Red oil (1.69 g, 59 %), prepared using similar conditions as described for **5a**. ^{1}H NMR (400 MHz, $CDCl_3$) δ 7.59 (dd, *J* = 8.0, 1.7 Hz, 1H), 7.57 (d, *J* = 1.7 Hz, 1H), 7.26 (d, *J* = 8.0, 1H), 2.23 (s, 3H), 1.26 (s, 6H); ^{13}C NMR (100 MHz, $CDCl_3$) δ 188.24, 153.34, 148.09, 136.56, 130.56, 121.73, 89.85, 53.94, 22.84, 15.30.

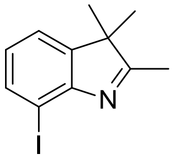

5d

Brown solid (1.21 g, 42 %), prepared using similar conditions as described for **5a**.

Synthesis of Compounds 4a-d

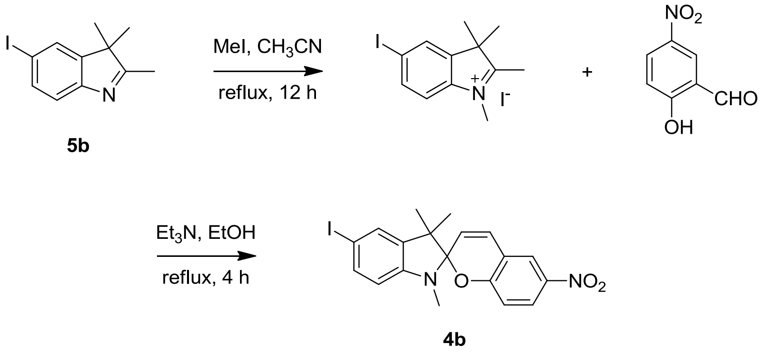

Compound **5b** (0.86 g, 3 mmol) was dissolved in 10 mL of CH_3CN and MeI (1.9 mL, 30 mmol) was added to the solution. The mixture was stirred at 65 ^{o}C for overnight. The precipitate formed was filtered and washed with Et_2O. The solid and 5-

nitrosalicylaldehyde (461 mg, 2.76 mmol) were dissolved in 15 mL of ethanol then Et_3N (0.57 mL, 4.14 mmol) was added. The mixture was refluxed for 4 h then cooled to room temperature. The volatile components were removed under vacuum. The residue was purified by flash chromatography (20 to 40 % CH_2Cl_2/Hexanes) to afford the pure product **4b** (1.08 g, 80 %) as a yellow solid. ^1H NMR (400 MHz, $CDCl_3$) δ 8.55-7.99 (m, 2H), 7.47 (dd, *J* = 8.2, 1.8 Hz, 1H), 7.33 (d, *J* = 1.8 Hz, 1H), 6.94 (d, *J* = 10.3 Hz, 1H), 6.76 (d, *J* = 8.4 Hz, 1H), 6.35 (d, *J* = 8.2 Hz, 1H), 5.83 (d, *J* = 10.3 Hz, 1H), 2.72 (s, 3H), 1.27 (s, 3H), 1.18 (s, 3H). ^{13}C NMR (100 MHz, $CDCl_3$) δ 159.38, 147.41, 141.01, 138.87, 136.44, 130.38, 128.51, 125.86, 122.67, 120.92, 118.46, 115.37, 109.33, 106.00, 80.78, 52.13, 28.76, 25.67, 19.72. HRMS (ESI) m/z calculated for $C_{19}H_{18}IN_2O_3$ ($[M+H]^+$) 449.0357; found 449.0363.

Compound **4a, 4c, 4d** were prepared using similar conditions as described for **4b**.

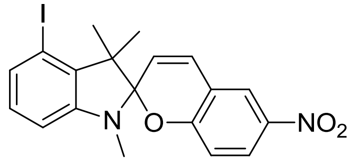

4a

White solid (635 mg, 84 %). ^1H NMR (400 MHz, $CDCl_3$) δ 8.05-8.00 (m, 2H), 7.29 (dd, *J* = 8.0, 0.8 Hz, 1H), 6.96 (d, *J* = 10.4 Hz, 1H), 6.87 (t, *J* = 7.9 Hz, 1H), 6.77 (d, *J* = 8.6 Hz, 1H), 6.52 (dd, *J* = 7.8, 0.6 Hz, 1H), 5.82 (d, *J* = 10.4 Hz, 1H), 2.73 (s, 3H), 1.45 (s, 3H), 1.25 (s, 3H); ^{13}C NMR (100 MHz, $CDCl_3$) δ 159.68, 149.57, 140.95, 135.70, 131.00, 129.40, 128.93, 125.95, 122.76, 120.85, 118.28, 115.34, 106.98, 106.58, 89.94, 54.87, 28.85, 22.15, 19.91. HRMS (ESI) m/z calculated for $C_{19}H_{18}IN_2O_3$ ($[M+H]^+$) 449.0357; found 449.0362.

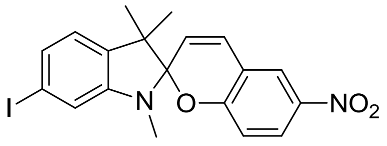

4c

White solid (168 mg, 75 %). ^{1}H NMR (400 MHz, $CDCl_3$) δ 8.05-8.00 (m, 2H), 7.21 (dd, *J* = 7.6, 1.5 Hz, 1H), 6.94 (d, *J* = 10.3 Hz, 1H), 6.87 (d, *J* = 1.4 Hz, 1H), 6.82 (d, *J* = 7.6 Hz, 1H), 6.77 (d, *J* = 8.4 Hz, 1H), 5.84 (d, *J* = 10.3 Hz, 1H), 2.72 (s, 3H), 1.27 (s, 3H), 1.17 (s, 3H); ^{13}C NMR (100 MHz, $CDCl_3$) δ 159.36, 148.99, 141.02, 136.08, 128.54, 128.49, 125.87, 123.29, 122.67, 120.97, 118.46, 116.00, 115.39, 106.03, 92.49, 51.99, 28.77, 25.59, 19.67. HRMS (ESI) m/z calculated for $C_{19}H_{18}IN_2O_3$ ($[M+H]^+$) 449.0357; found 449.0360.

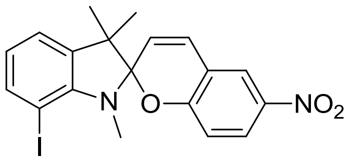

4d

Yellow solid (415 mg, 27 %). ^{1}H NMR (400 MHz, $CDCl_3$) δ 8.05-8.00 (m, 2H), 7.61 (dd, *J* = 8.0, 1.2 Hz, 1H), 7.03 (dd, *J* = 7.2, 1.2 Hz, 1H), 6.94 (d, *J* = 10.3 Hz, 1H), 6.79 (d, *J* = 8.8 Hz, 1H), 6.61-6.56 (m, 1H), 5.83 (d, *J* = 10.4 Hz, 1H), 3.16 (s, 3H), 1.27 (s, 3H), 1.15 (s, 3H); ^{13}C NMR (100 MHz, $CDCl_3$) δ 159.43, 147.17, 141.01, 140.38, 139.16, 128.63, 125.90, 122.70, 121.82, 121.55, 121.42, 118.31, 115.38, 107.14, 70.80, 51.03, 32.47, 25.93, 19.74. HRMS (ESI) m/z calculated for $C_{19}H_{18}IN_2O_3$ ($[M+H]^+$) 449.0357; found 449.0362.

Synthesis of Compound 4e-h

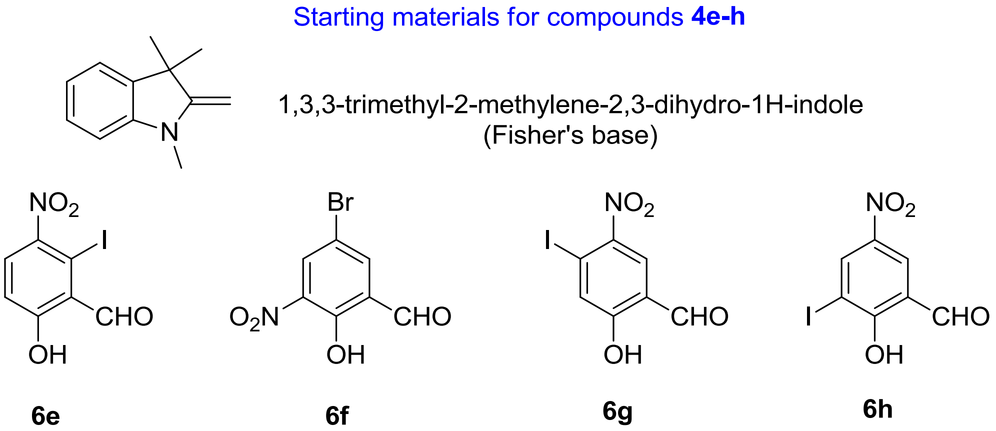

The starting materials for compounds **4e-h** are showed in the above figure. Compounds **6f**, **6h** and Fisher's base are commercially available and used as received. Attempt to prepare 5-iodo-3-nitrosalicylaldehyde lead to an inseparable mixture, thus the commercially available compound **6f** was used instead. Compounds **6e** and **6g** were prepared according to literature procedures.[1]

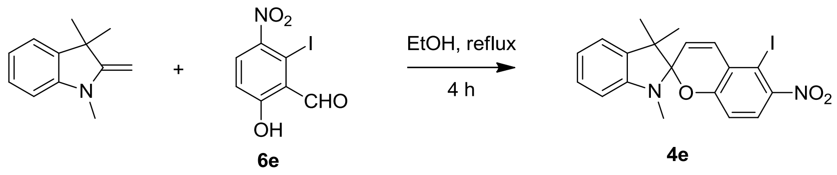

Compound **6e** (368 mg, 1.25 mmol) was dissolved in 10 mL of EtOH and a solution of Fisher's base (217 mg, 1.25 mmol) in 5 mL of EtOH was added. The reaction mixture was refluxed for 4 h then cooled to room temperature. The solvent was removed under vacuum and the residue was purified by flash chromatography (30 to 50 % CH_2Cl_2/Hexanes) to afford compound **4e** (533 mg, 95 %) as a yellow solid. ^{1}H NMR (400 MHz, $CDCl_3$) δ 7.64 (d, J = 8.9 Hz, 1H), 7.35 (d, J = 10.7 Hz, 1H), 7.23 (t, J = 7.7 Hz, 1H), 7.11 (d, J = 7.2 Hz, 1H), 6.91 (t, J = 7.2 Hz, 1H), 6.76 (d, J = 8.9 Hz, 1H), 6.58 (d, J = 7.7 Hz, 1H), 5.91 (d, J = 10.7 Hz, 1H), 2.75 (s, 3H), 1.31 (s, 3H), 1.22 (s, 3H); ^{13}C NMR (100 MHz, $CDCl_3$) δ 157.63, 147.55, 147.16, 135.86, 133.56, 127.81, 126.78, 123.62, 122.95, 121.48, 119.75, 115.60, 106.99, 105.97, 91.71, 52.17, 28.84, 25.72, 19.93. HRMS (ESI) m/z calculated for $C_{19}H_{18}IN_2O_3$ ($[M+H]^+$) 449.0357; found 449.0358.

Compounds **4f-h** were prepared using similar conditions as described for compound **4e**.

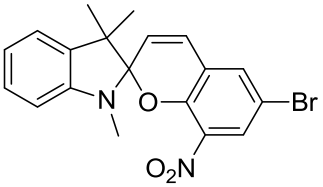

4f

Yellow solid (439 mg, 55 %). ^{1}H NMR (400 MHz, $CDCl_3$) δ 7.81 (d, *J* = 2.4 Hz, 1H), 7.39 (d, *J* = 2.4 Hz, 1H), 7.18 (td, *J* = 7.7, 1.2 Hz, 1H), 7.09 – 7.06 (m, 1H), 6.90 – 6.84 (m, 2H), 6.55 (d, *J* = 7.7 Hz, 1H), 5.92 (d, *J* = 10.4 Hz, 1H), 2.74 (s, 3H), 1.36 (s, 3H), 1.20 (s, 3H); ^{13}C NMR (100 MHz, $CDCl_3$) δ 147.41, 147.19, 137.38, 135.70, 133.39, 127.68, 127.46, 127.36, 123.26, 122.67, 121.33, 119.90, 110.29, 107.06, 52.24, 28.76, 25.92, 19.91. HRMS (ESI) m/z calculated for $C_{19}H_{18}BrN_2O_3$ ($[M+H]^+$) 401.0495; found 401.0501.

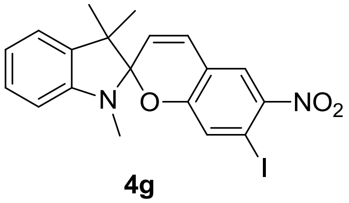

4g

Yellow solid (235 mg, 48 %). ^{1}H NMR (400 MHz, $CDCl_3$) δ 7.82 (s, 1H), 7.41 (s, 1H), 7.24 – 7.18 (m, 1H), 7.12 – 7.07 (m, 1H), 6.93 – 6.87 (m, 2H), 6.57 (d, *J* = 7.7 Hz, 1H), 5.90 (d, *J* = 10.3 Hz, 1H), 2.75 (s, 3H), 1.31 (s, 3H), 1.19 (s, 3H); ^{13}C NMR (100 MHz, $CDCl_3$) δ 157.62, 147.48, 144.52, 135.87, 128.19, 127.84, 127.65, 124.20, 122.39, 121.48, 119.87, 118.71, 107.09, 106.67, 87.40, 52.32, 28.84, 25.81, 19.88. HRMS (ESI) m/z calculated for $C_{19}H_{18}IN_2O_3$ ($[M+H]^+$) 449.0357; found 449.0363.

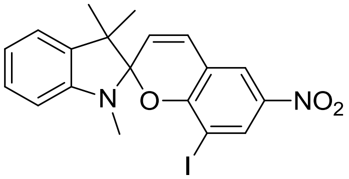

4h

Green solid (361 mg, 40 %). ^{1}H NMR (400 MHz, $CDCl_3$) δ 8.46 (d, *J* = 2.6 Hz, 1H), 7.99 (d, *J* = 2.6 Hz, 1H), 7.21 (td, *J* = 7.7, 1.3 Hz, 1H), 7.12 – 7.09 (m, 1H), 6.91 (dd, *J* = 7.4, 0.9 Hz, 1H), 6.87 (d, *J* = 10.3 Hz, 1H), 6.58 (d, *J* = 7.7 Hz, 1H), 5.88 (d, *J* = 10.3 Hz, 1H),

2.70 (s, 3H), 1.31 (s, 3H), 1.21 (s, 3H); ^{13}C NMR (100 MHz, $CDCl_3$) δ 158.15, 147.40, 141.34, 135.89, 134.46, 128.17, 127.69, 122.29, 122.18, 121.38, 119.92, 118.40, 108.51, 107.17, 82.53, 52.15, 28.91, 25.68, 20.21. HRMS (ESI) m/z calculated for $C_{19}H_{18}IN_2O_3$ ($[M+H]^+$) 449.0357; found 449.0363.

Synthesis of TzBIPS-NHS:

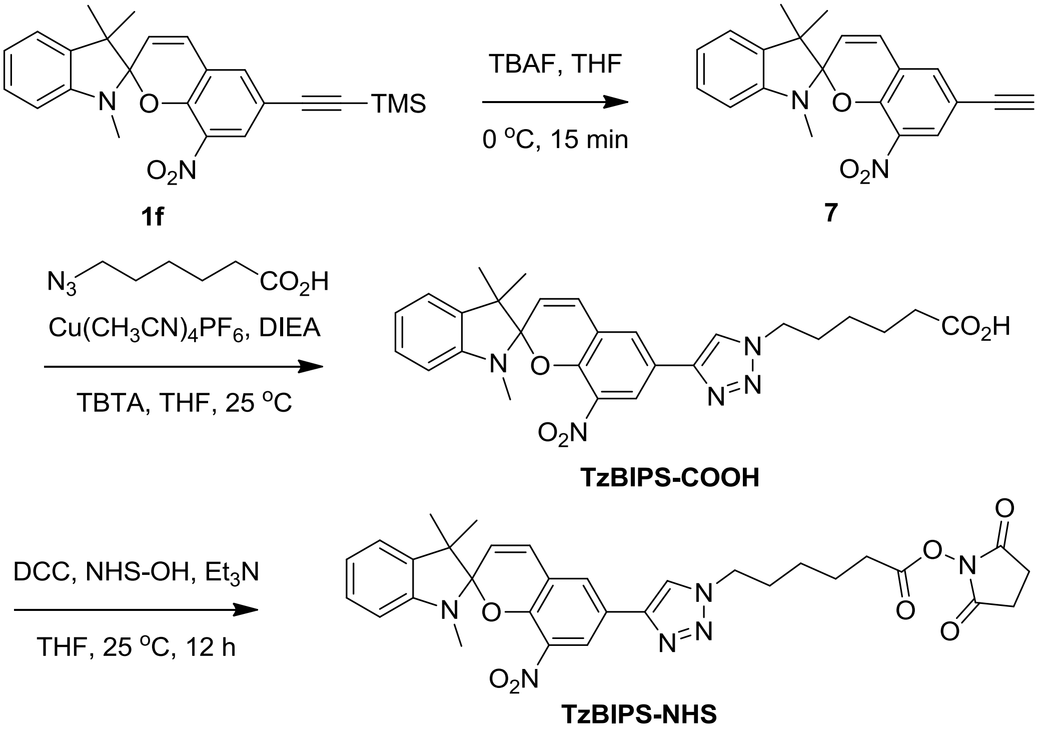

*Step 1: Synthesis of compound **7***

To a stirred solution of compound **1f** (97 mg, 0.23 mmol) in 5 mL of dry THF was added TBAF (1 M in THF, 0.28 mL) at 0 °C. After stirred at 0 °C for 15 min, the reaction mixture was diluted with EtOAc then washed with water and brine. The organic layer was dried over Na_2SO_4 then concentrated. The residue was purified by flash chromatography (silica gel, 10 to 20 % EtOAc/Hexanes) to afford the pure product **7** (42 mg, 53 %) as a blue solid. ^1H NMR (400 MHz, $CDCl_3$) δ 7.81 (d, J = 2.0 Hz, 1H), 7.37 (d, J = 2.0 Hz, 1H), 7.17 (td, J = 7.7, 1.2 Hz, 1H), 7.07 (d, J = 7.2 Hz, 1H), 6.91 – 6.84 (m, 2H), 6.54 (d, J = 7.7 Hz, 1H), 5.90 (d, J = 10.4 Hz, 1H), 3.05 (s, 1H), 2.74 (s, 3H), 1.35 (s, 3H), 1.20 (s, 3H).

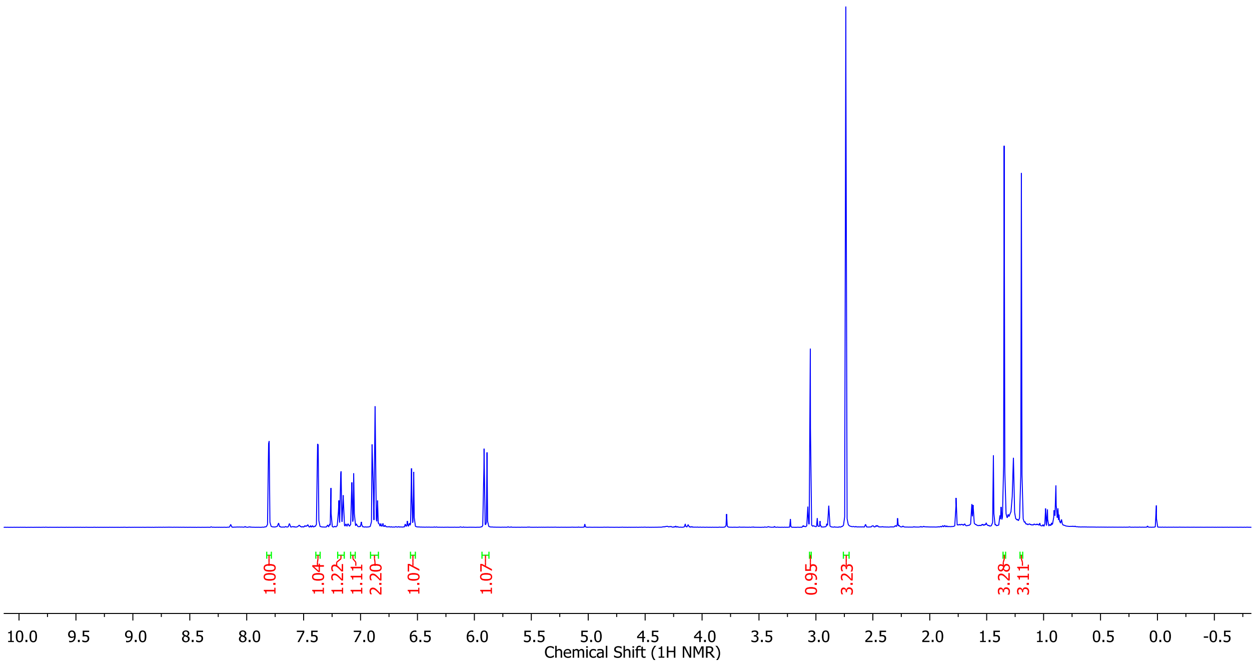

Step 2: Synthesis of TzBIPS-COOH

To a stirred solution of compound **7** (42 mg, 0.12 mmol), $Cu(CH_3CN)_4PF_6$ (9 mg, 0.024 mmol) and tris[(1-benzyl-1H-1,2,3-triazol-4-yl)methyl]amine (TBTA, 3 mg, 0.006 mmol) in 5 mL of THF was added DIEA (0.21 mL, 1.2 mmol), followed by a solution of 6-azido-hexanoic acid (57 mg, 0.36 mmol) in 1 mL of THF. The reaction mixture was stirred at room temperature for 2 h then diluted with EtOAc. The mixture was acidified with 1N HCl then washed with water and brine. The organic layer was dried over Na2SO4 and concentrated. The residue was purified by flash chromatography (silica gel, 0 to 20 % MeOH/DCM) to afford the pure product **TzBIPS-COOH** (36 mg, 60 %) as a purple solid. ^{1}H NMR (400 MHz, $CDCl_3$) δ 8.00 (d, *J* = 2.1 Hz, 1H), 7.92 (d, *J* = 2.1 Hz, 1H), 7.77 (s, 1H), 7.17 (td, *J* = 7.6, 1.2 Hz, 1H), 7.07 (d, *J* = 6.4 Hz, 1H), 6.99 (d, *J* = 10.4 Hz, 1H), 6.86 (t, *J* = 7.4 Hz, 1H), 6.54 (d, *J* = 7.7 Hz, 1H), 5.91 (d, *J* = 10.3 Hz, 1H), 4.42 (t, *J* = 7.0 Hz, 2H), 2.75 (s, 3H), 2.37 (t, *J* = 7.2 Hz, 2H), 2.01 – 1.96 (m, 2H), 1.73 – 1.68 (m, 2H), 1.46 – 1.38 (m, 2H), 1.36 (s, 3H), 1.20 (s, 3H). HRMS (ESI) m/z calculated for $C_{27}H_{28}N_5O_5$ ($[M-H]^-$) 502.2096; found 502.2089.

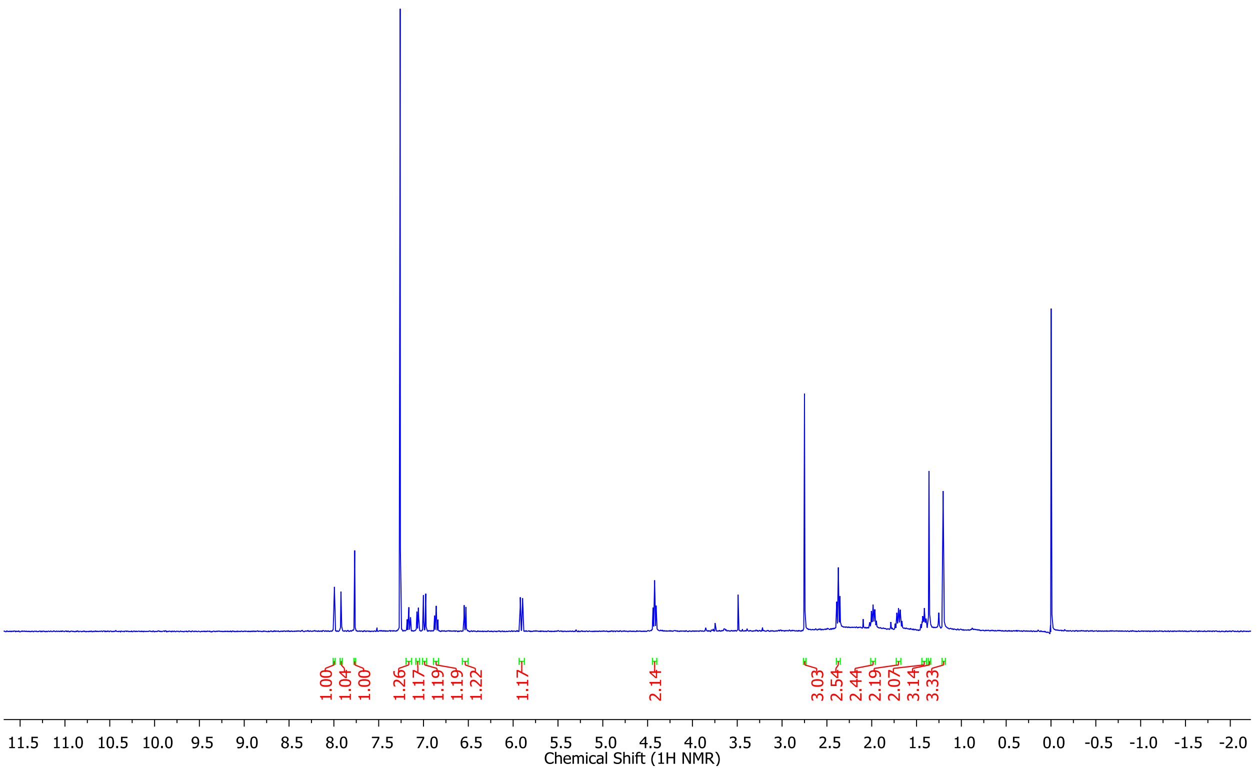
1.00
1.04
1.00
1.26
1.17
1.19
1.19
1.22
1.17
2.14
3.03
2.54
2.44
2.19
2.07
3.14
3.33
11.5 11.0 10.5 10.0 9.5 9.0 8.5 8.0 7.5 7.0 6.5 6.0 5.5 5.0 4.5 4.0 3.5 3.0 2.5 2.0 1.5 1.0 0.5 0.0 -0.5 -1.0 -1.5 -2.0
Chemical Shift (1H NMR)

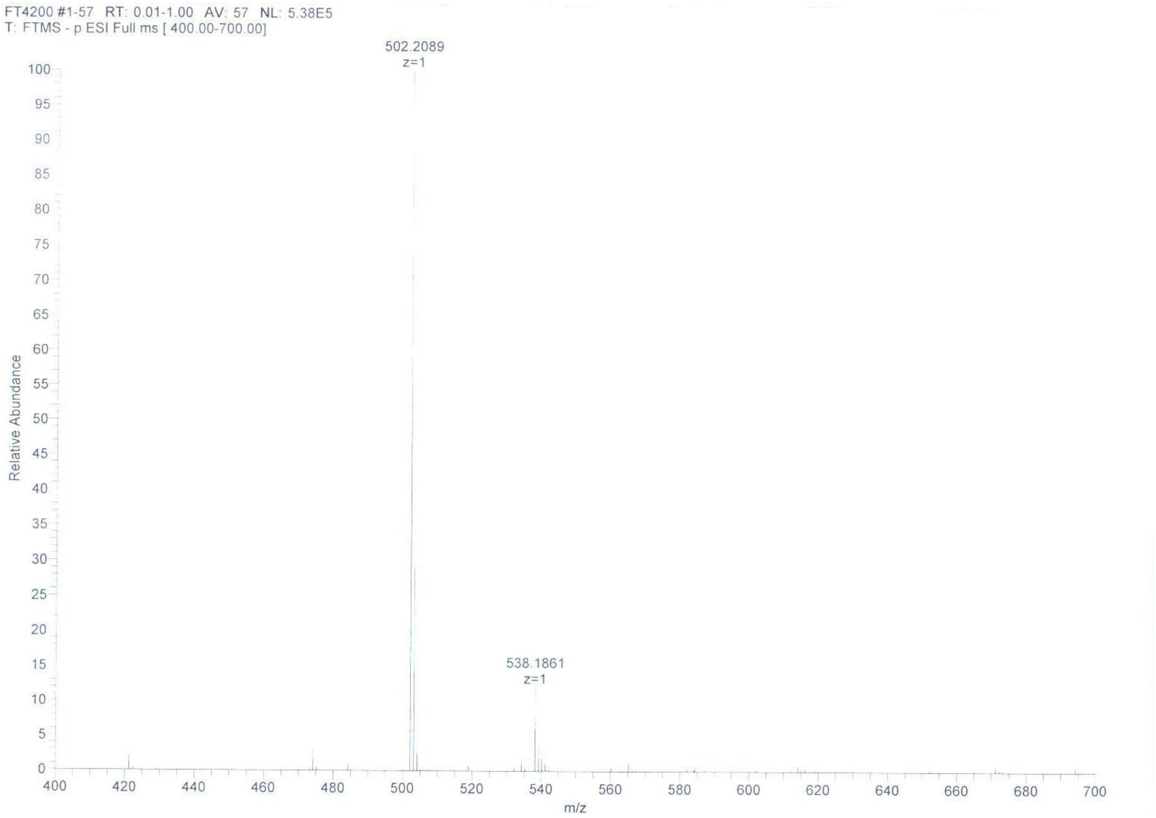
FT4200 #1-57 RT: 0.01-1.00 AV: 57 NL: 5.38E5
T: FTMS - p ESI Full ms [400.00-700.00]
502.2089
z=1
538.1861
z=1
Relative Abundance
m/z

Step 3: synthesis of TzBIPS-NHS

To a stirred solution of TzBIPS-COOH (4 mg, 0.008 mmol), dicyclohexcylcarbodiimide (DCC, 2 mg, 0.0096 mmol) and N-hydroxysuccinimide (NHS-OH, 1.2 mg, 0.01 mmol) in 3 mL of THF was added triethylamine (4 μL, 0.024 mmol). The reaction mixture was stirred at room temperature overnight then filtered and concentrated. The residue was used directly without further purification.

References:

1. (a) Inouye, M.; Akamatsu, K.; Nakazumi, H. *J. Am. Chem. Soc.* **1997**, *119*, 9160-9165; (b) Hodgson, H. H.; Jenkinson, T. A. *J. Chem. Soc.* **1928**, 2272-80.

Spectroscopic and Photochromic Properties of Compounds

a. b.

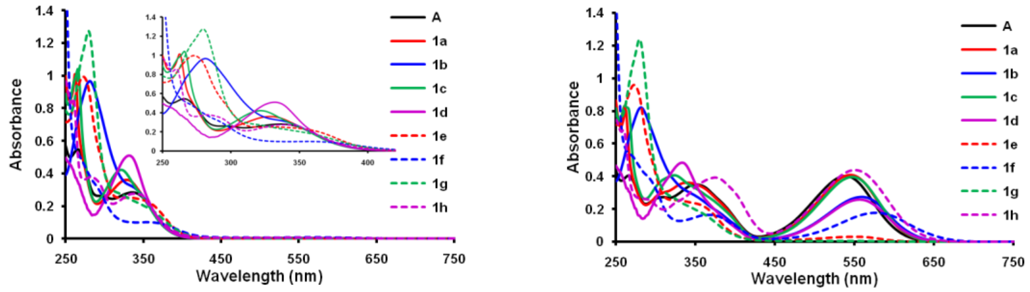

c. d.

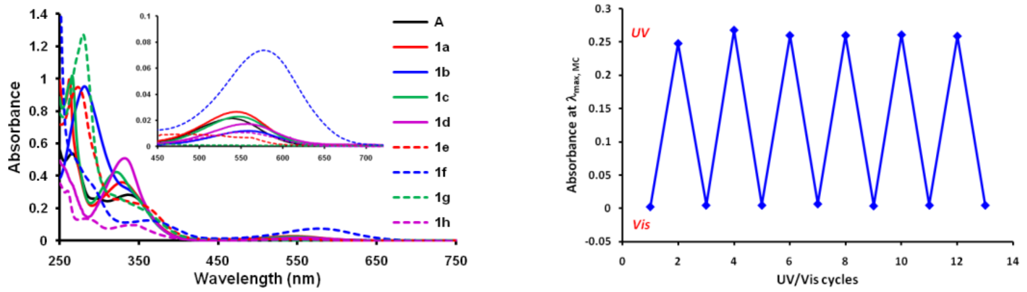

Figure S1. Absorbance spectra of compounds **1a-h** (30 µM in EtOH): (**a**) as dissolved in solution; (**b**) irradiated with 365 nm light for 30 s; (**c**) irradiated with 405 nm light for 60 s; (**d**) illustrative example of reversibility of photoswitching for compound **1b**: irradiated alternatively with 365 nm light for 30 s and 530 nm light for 5 min.

a. b.

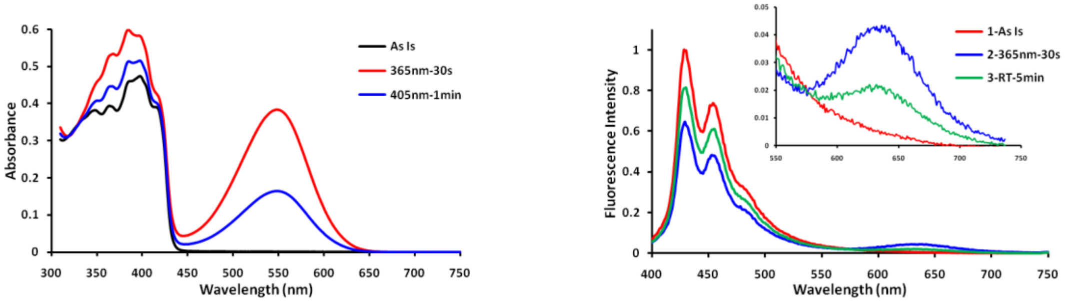

Figure S2. Optical properties of compound **2a**: (**a**) comparison of absorbance of **2a** in ethanol: as dissolved in solution (black); irradiated with 365 nm light for 30 s (red); irradiated with 405 nm light for 60 s (blue); (**b**) fluorescence spectra of **2a** in ethanol (30 µM), as dissolved in solution (red); irradiated with 365 nm light for 30 s (blue); thermal recovery of the irradiated solution in the dark at room temperature, fluorescence spectrum after 5 min at room temperature is shown (green).

a. b.

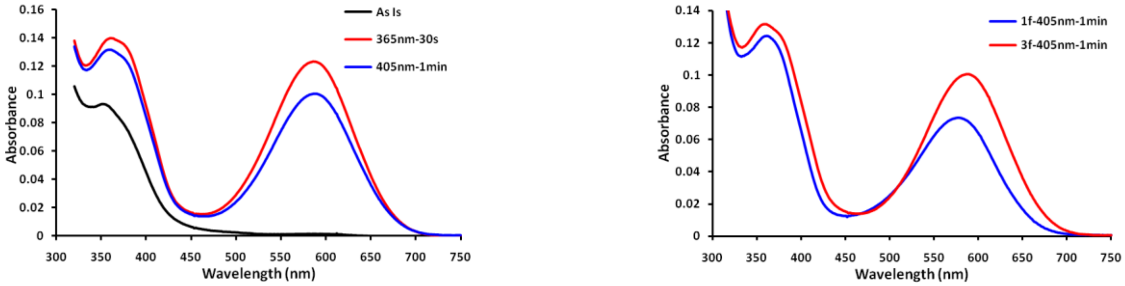

Figure S3. (**a**) Comparison of absorbance of **3f** in ethanol (30 µM): as dissolved in solution (black); irradiated with 365 nm light for 30 s (red); irradiated with 405 nm light for 60 s (blue); (**b**) Comparison of absorbance spectra of **1f** and **3f** in ethanol (30 µM), the solution is irradiated with 405 nm light for 60 s.

(**a**)

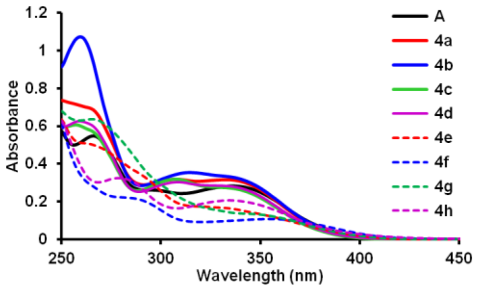

(**b**)

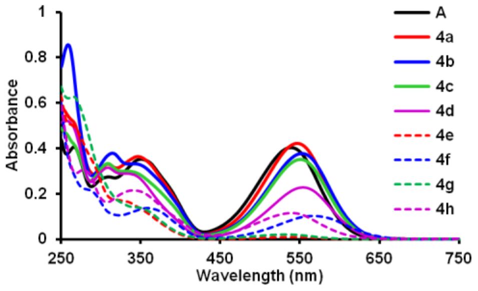

(**c**)

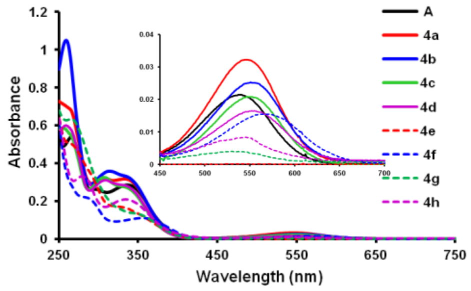

Figure S4: Absorbance spectra of compounds **4a-h** and reference compound **A** (30 µM in EtOH): (**a**) as dissolved in solution; (**b**) irradiated with 365 nm light for 30 s; (**c**) irradiated with 405 nm light for 60 s.

(**a**)

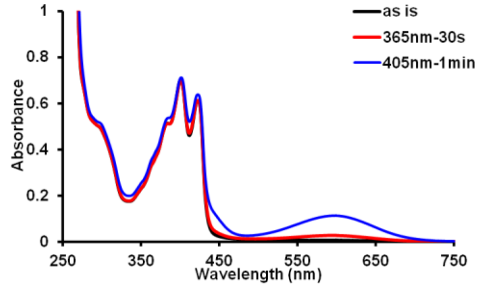

(**b**)

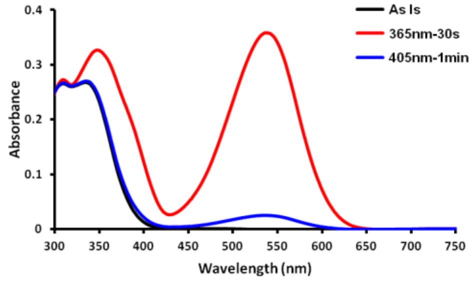

Figure S5: Absorbance spectra of compounds (**a**) **2f** and (**b**) **3a** (30 µM in EtOH): as dissolved in solution (black curve); irradiated with 365 nm light for 30 s (red curve); irradiated with 405 nm light for 60 s (blue curve).

Kinetic profiles of thermal decay of the MC states in the dark at room temperature:

(a)

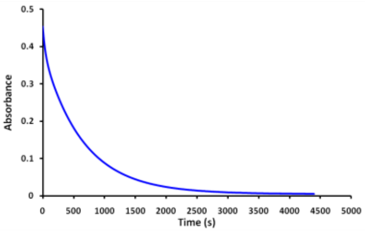

(b)

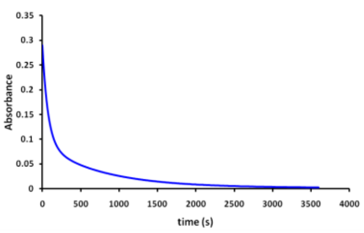

(c)

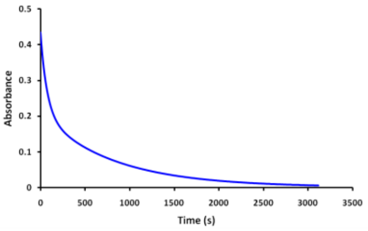

(d)

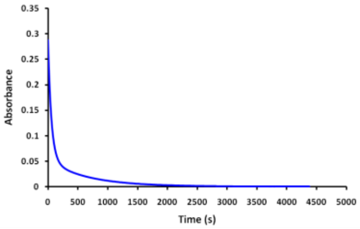

(e)

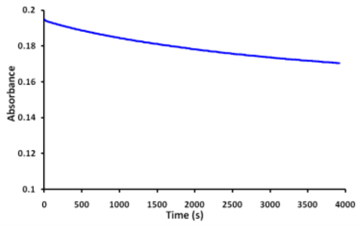

(f)

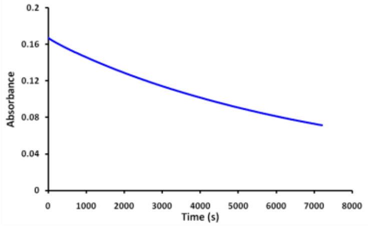

(g)

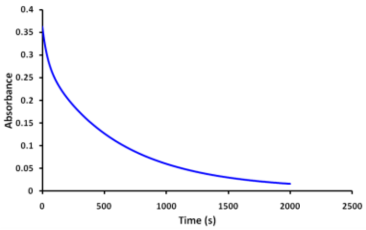

(h)

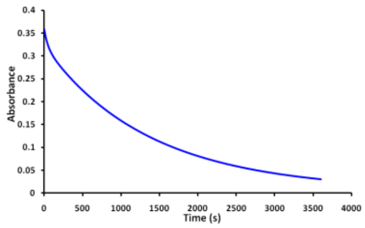

(i)

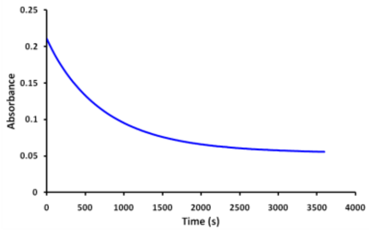

Figure S6. Kinetic profiles of thermal decay of the MC states in the dark at room temperature for compounds **1-3:** (**a**) **1a**; (**b**) **1b**; (**c**) **1c**; (**d**) **1d**; (**e**) **1f**; (**f**) **1h**; (**g**) **2a**; (**h**) **3a**; (**i**) **3f**. For compounds **1e**, **1g** and **2f**, photochemistry is not efficient to be measured.

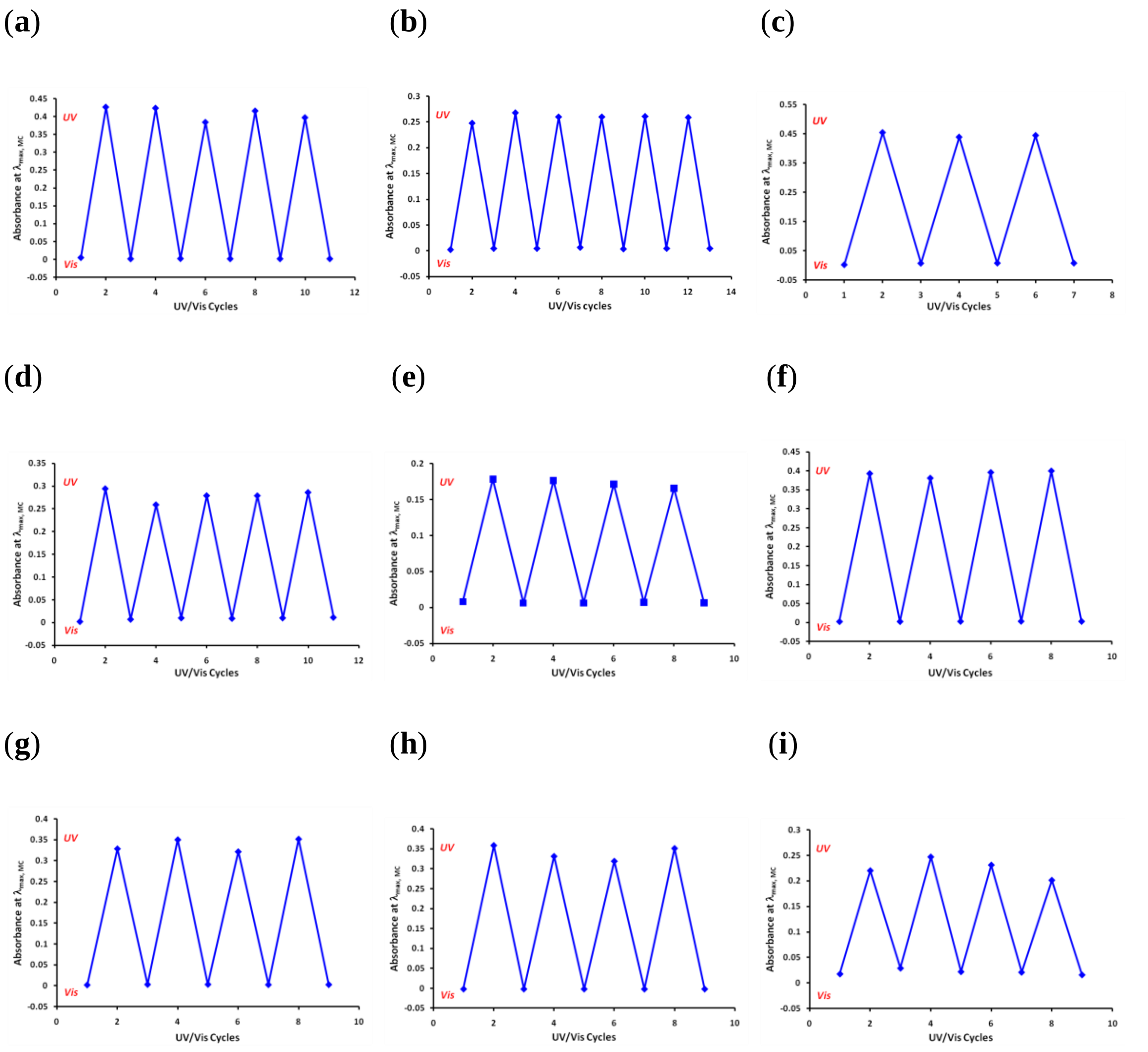

Figure S7. Demonstration of the reversibility of photoswitching for compounds **1-3:** (**a**) **1a**; (**b**) **1b**; (**c**) **1c**; (**d**) **1d**; (**e**) **1f**; (**f**) **1h**; (**g**) **2a**; (**h**) **3a**; (**i**) **3f**. For compounds **1e**, **1g** and **2f**, photochemistry is not efficient to be measured. The solution was irradiated alternatively with 365 nm light for 30 s and 530 nm light for 5 min then the absorption spectrum was recorded.

Table S1. Spectroscopic properties of the new BIPS derivatives **1-4** (30 µM in ethanol)

	$\lambda_{max,SP}$ (nm)	$\varepsilon_{max,SP}$ ($M^{-1}cm^{-1}$)	$\varepsilon_{405,SP}$ ($M^{-1}cm^{-1}$)	$\lambda_{max,MC}$ (nm)	$\lambda_{Fl, MC}$ (nm)	**A_0** (30s_365nm)	**A_0** (60s_405nm)	$t_{1/2}$ (s)
**A**	336	9600	300	539	630	0.400	0.019	1386
**1a**	329	12100	300	545	635	0.409	0.025	359
**1b**	336	10600	300	559	640	0.272	0.009	82
**1c**	322	14500	300	548	640	0.395	0.017	127
**1d**	332	17200	300	557	640	0.257	0.014	51
**1e**	331	8500	700	N/A	N/A	N/A	N/A	N/A
**1f**	357	3600	1300	578	680	0.173	0.067	stable
**1g**	336	6800	900	N/A	N/A	N/A	N/A	N/A
**1h**	337	9300	700	550	650	0.392	0.028	5715
**2a**	398	15500	14500	548	-	0.382	0.162	273
**2f**	423	20900	21600	599	-	0.022	0.106	N/A
**3a**	334	8800	200	538	630	0.342	0.026	821
**3f**	352	3100	1300	585	690	0.112	0.091	830
**4a**	332	10900	200	547	630	0.420	0.029	188
**4b**	336*	11000	300	552	635	0.377	0.020	142
**4c**	336*	8900	200	550	630	0.350	0.016	66
**4d**	336*	9400	200	553	630	0.227	0.012	32
**4e**	336*	5600	500	541	630	0.011	N/A	N/A
**4f**	355	3500	700	566	670	0.101	0.014	N/A
**4g**	350	4400	400	533	630	0.020	N/A	N/A
**4h**	335	7000	700	540	630	0.102	0.005	N/A

Optical switching of red-shifted BIPS probes in living cells

The most promising red-shifted optical switch probes **3f** is chosen for further testing in living cells. NIH 3T3 cells are incubated with 50 μM of probe in medium for 30 min at 37 ^oC followed by washing with fresh culture medium. Cells loaded with these probes are indistinguishable from control cells, indicating the probes are not cytotoxic. Strong and uniform MC fluorescence is observed for both probes in the cells indicating they penetrate the plasma membrane and are retained within the cytoplasm although in some cases vesicles staining is also found.

Optical switching of the red-shifted BIPS probes in living NIH 3T3 cells is carried out using a Zeiss 700 confocal microscope equipped with 405 nm and 555 nm lasers. A demonstration of optical switching of **3f** is shown in Figure S8a. As indicated in the scheme of Figure S8c, a single cycle of optical switching between the SP and MC states of **3f** is achieved by first irradiating the red-circled area in the field of view (Figure S8a) with two sequential scans at 405 nm. Exposure of the sample to 405 nm generates the fluorescent MC state, which is observed on exposing the entire field to 555 nm light. Some of the 555 nm excited MC-molecules may undergo a MC to SP transition. Consequently the MC-fluorescence signal diminishes to almost zero following five sequential scans of the entire field to 555 nm. The intensity of MC-fluorescence over a single cycle of optical swiching is only modulated within the red-circled area *ie* that exposed to both 405 nm and 555 nm (Figure S8a). Subsequent illumination of SP molecules in the same red circled area with two sequential scans at 405 nm, repopulates the MC state as seen by the increase in MC fluorescence, whereas no red fluorescence is detected outside of the circled area. Frames showing modulation of **3f** within the red circled region is shown for two cycles of optical switching (Figure S8a). The corresponding plot of the MC-intensity within the red circled region within a labeled cell is shown in Figure S8d. Faster cycling times can be realized by increasing the intensity of the 555 nm laser and by reducing the area of the exposed sample.

The robust and highly efficient optical switching of **3f** within living cells is realized

with power levels for the 405 and 555 nm lasers set at 10 % of the laser capacity. Even so only 2 scans are required to trigger the almost complete conversion of SP to MC in the 405 nm exposed region and only a handful of scans at 555 nm is required to convet all the MC to the SP state. These poperties suggest that the new red shifted BIPS exhibit superior performance in optical switching compared to previously reported probes. Also significant is that the MC-fluorescence signal reaches almost its initial value in the selected area after two scans of 405 nm irradiation indicating that optical switching of compound **3f** is reversible and robust even in living cells. The same sequence of 405 nm and 555 nm scans of a field of cells labeled with 6-NO_2-BIPS under the same conditions has no effect on the intensity of MC-fluorescence, indicating 405 nm does not trigger the SP to MC transition for this most widely use BIPS probe (Figure S8b).

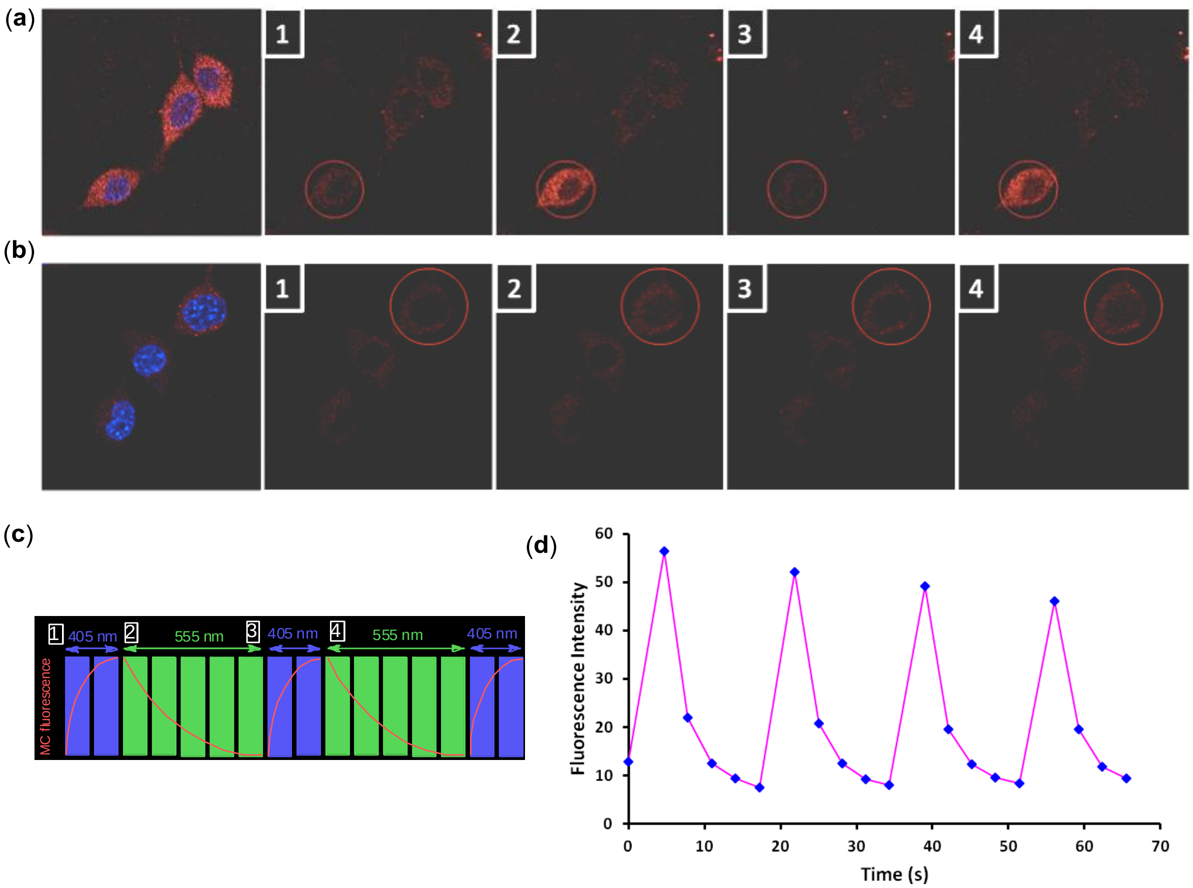

Figure S8. Optical switching of the probes (50 μM) in living NIH-3T3 cells: (**a**) probe **3f**; (**b**) reference compound 6-NO_2-BIPS: fluorescence pictures of all the cells in the imaging field with nuclei stained with DAPI (leftmost image); Montage of MC-fluorescence over two cycles of optical switching (1-4, corresponding to the time point 1-4 in Figure 6c); (**c**) Schematic representation of the laser perturbation sequence used for optical switching in this study; (**d**) MC-fluorescence intensity profile over multiple cycles of optical switching taken from the cell within the red circle in Figure S8a.

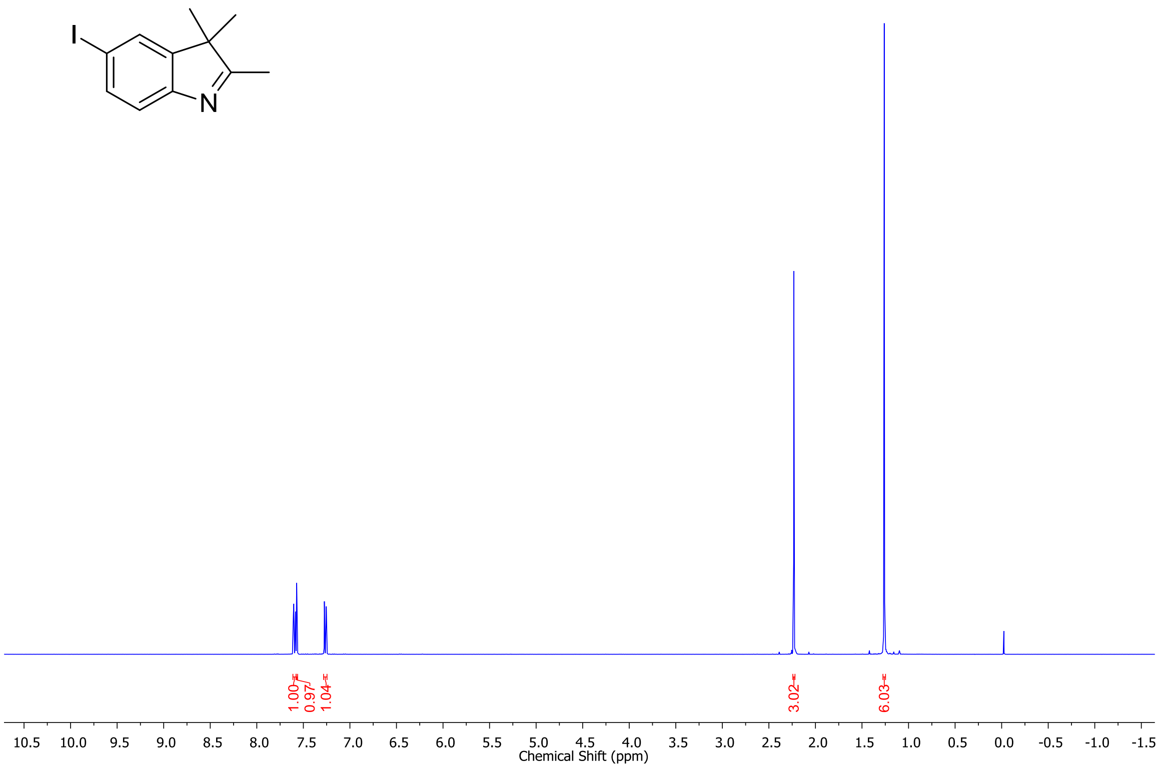

I
N
1.00
0.97
1.04
3.02
6.03
10.5 10.0 9.5 9.0 8.5 8.0 7.5 7.0 6.5 6.0 5.5 5.0 4.5 4.0 3.5 3.0 2.5 2.0 1.5 1.0 0.5 0.0 -0.5 -1.0 -1.5
Chemical Shift (ppm)

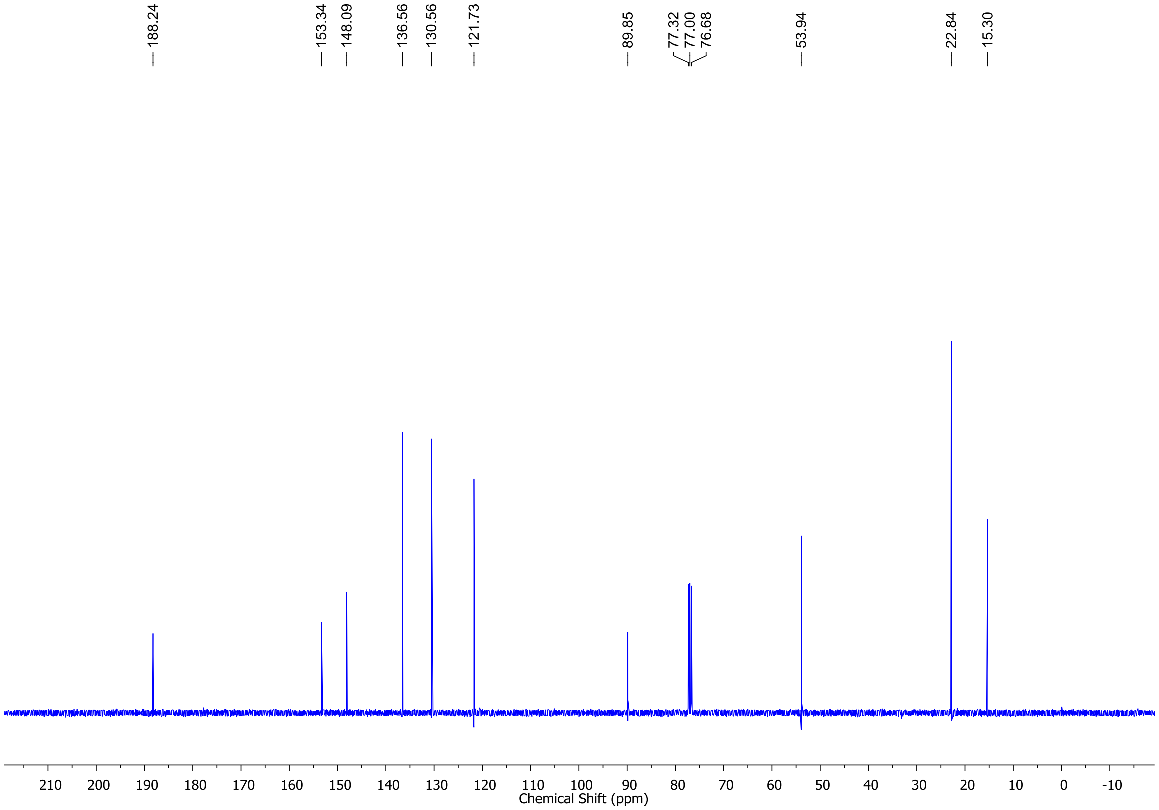

188.24
153.34
148.09
136.56
130.56
121.73
89.85
77.32
77.00
76.68
53.94
22.84
15.30
210 200 190 180 170 160 150 140 130 120 110 100 90 80 70 60 50 40 30 20 10 0 -10
Chemical Shift (ppm)

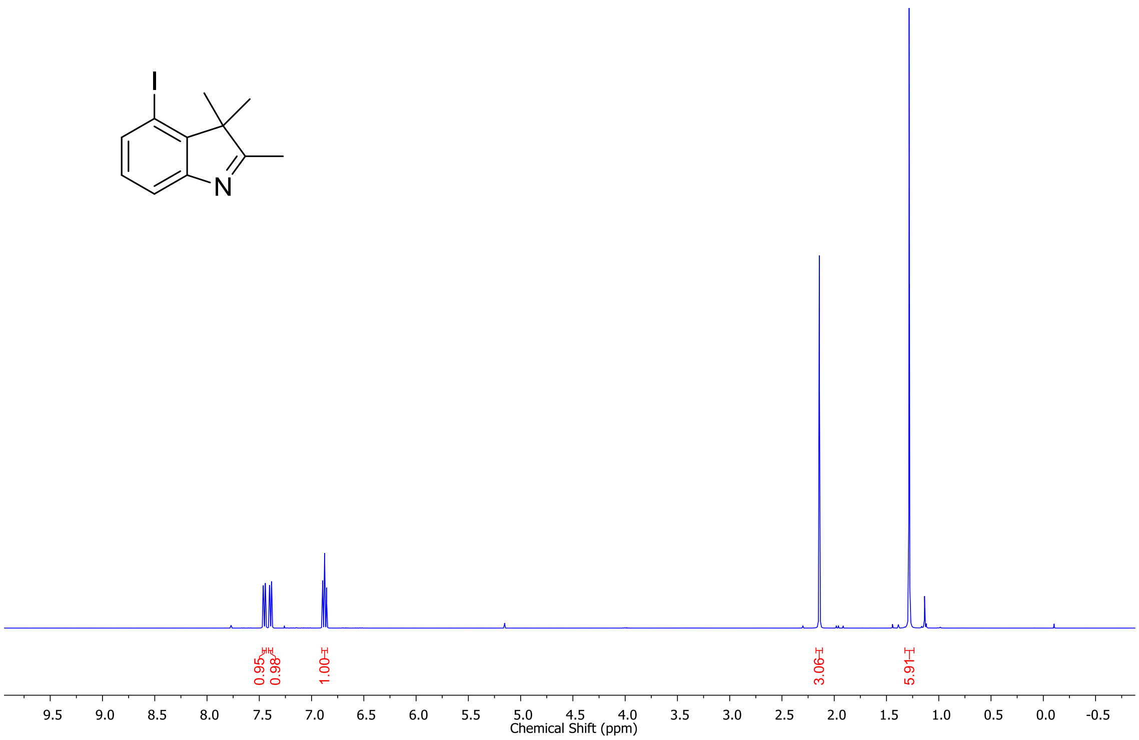
I
N
0.95
0.98
1.00
3.06
5.91
9.5 9.0 8.5 8.0 7.5 7.0 6.5 6.0 5.5 5.0 4.5 4.0 3.5 3.0 2.5 2.0 1.5 1.0 0.5 0.0 -0.5
Chemical Shift (ppm)

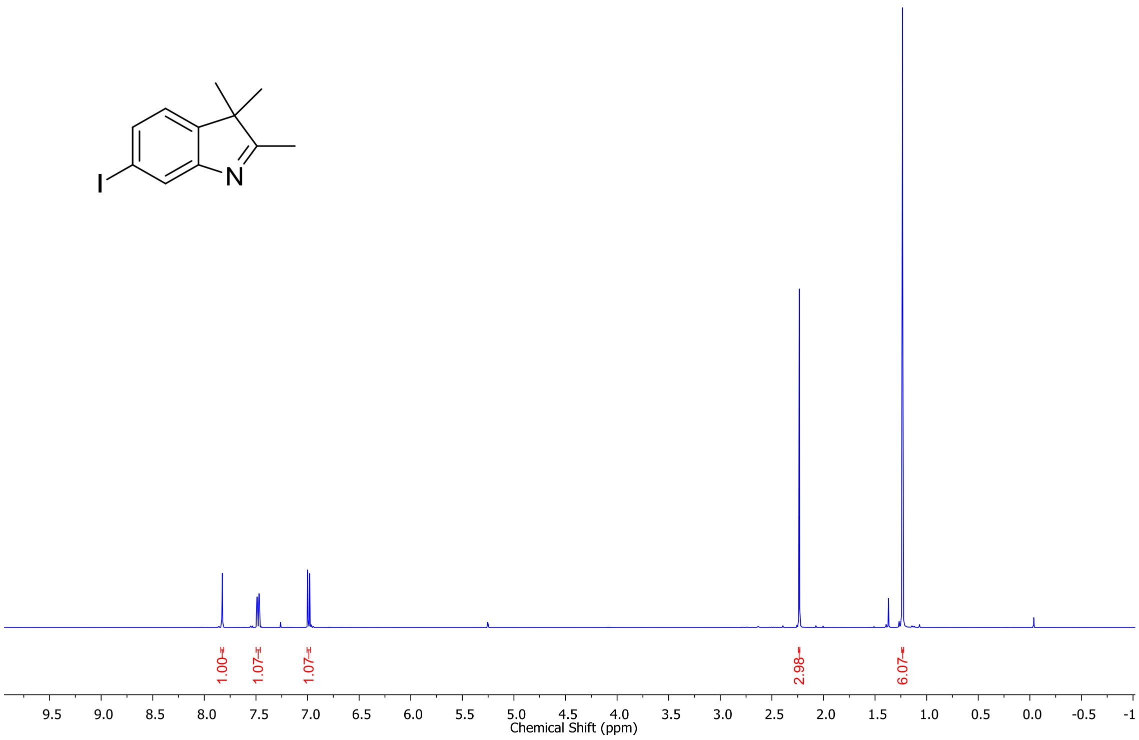
I
N
1.00
1.07
1.07
2.98
6.07
9.5 9.0 8.5 8.0 7.5 7.0 6.5 6.0 5.5 5.0 4.5 4.0 3.5 3.0 2.5 2.0 1.5 1.0 0.5 0.0 -0.5 -1
Chemical Shift (ppm)

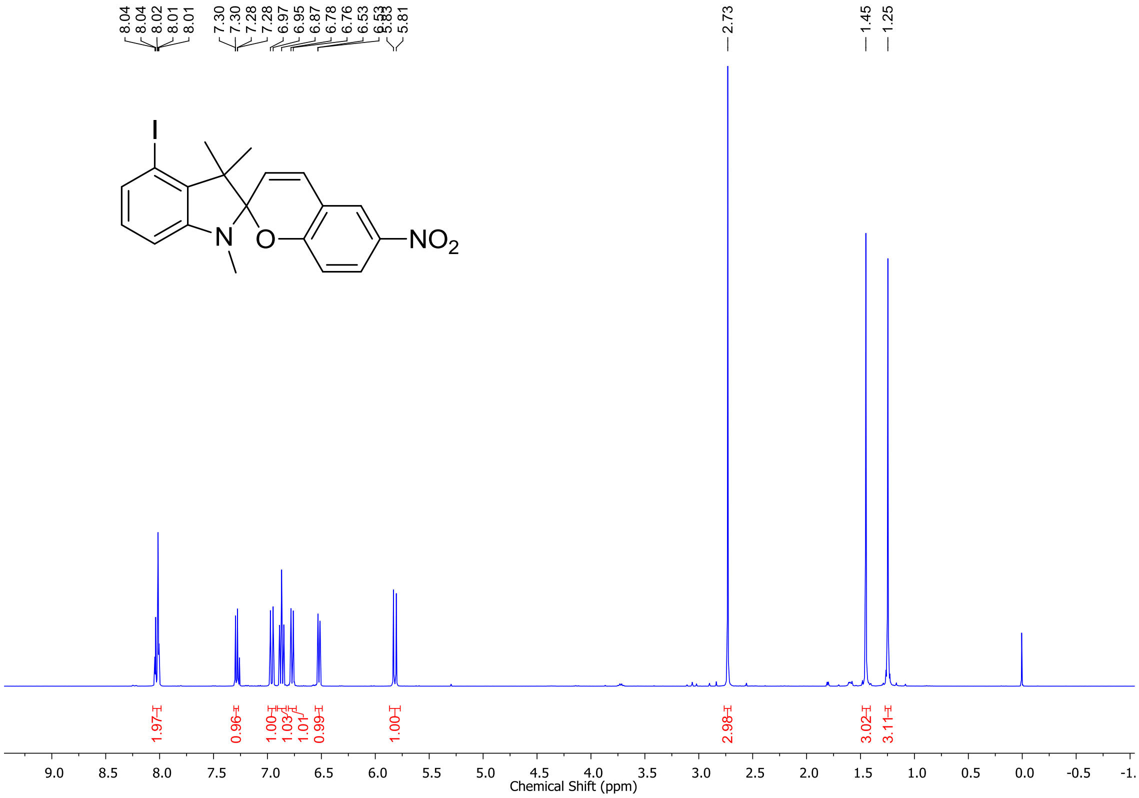

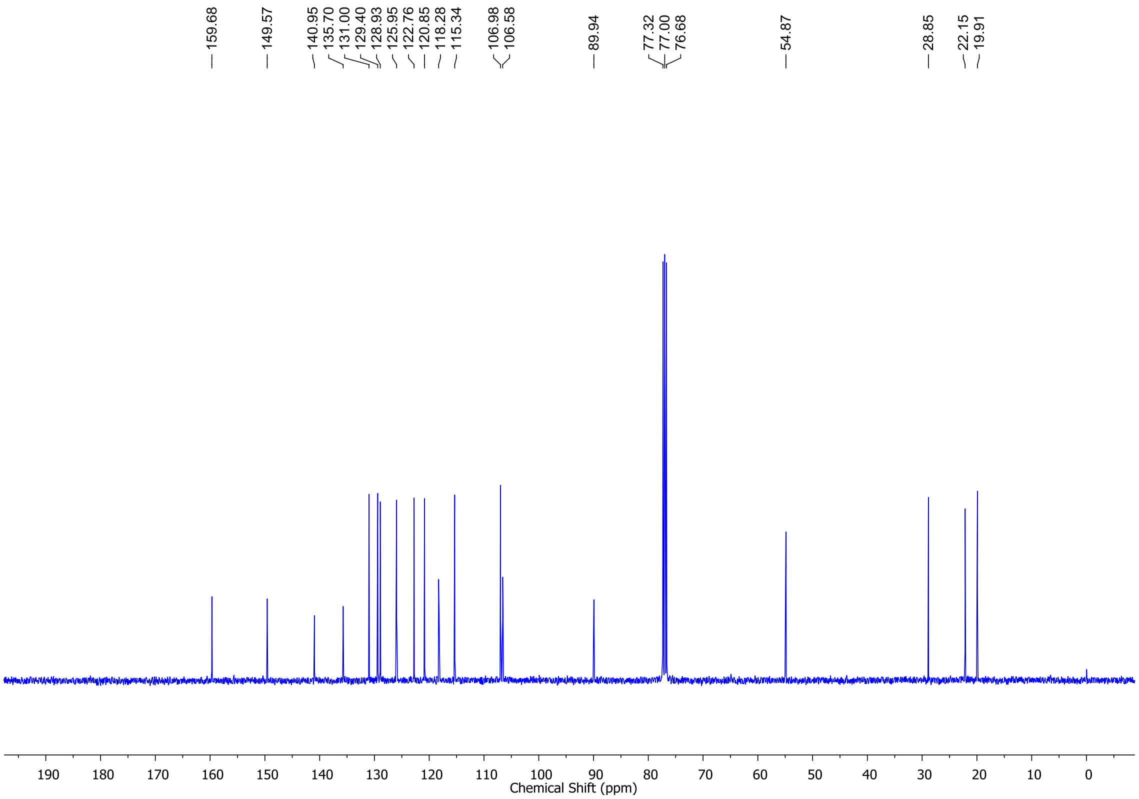

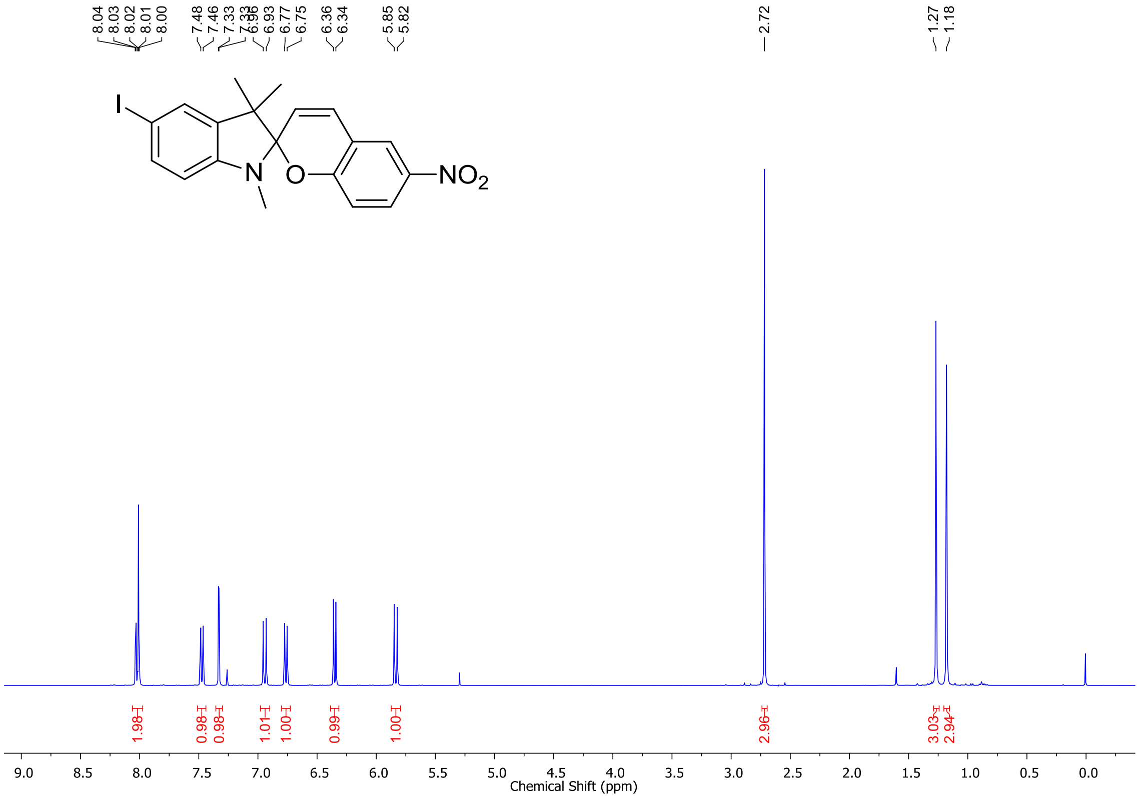

8.04
8.03
8.02
8.01
8.00
7.48
7.46
7.33
7.33
6.96
6.93
6.77
6.75
6.36
6.34
5.85
5.82
2.72
1.27
1.18
I
N
O
NO2
1.98
0.98
0.98
1.01
1.00
0.99
1.00
2.96
3.03
2.94
9.0
8.5
8.0
7.5
7.0
6.5
6.0
5.5
5.0
4.5
4.0
3.5
3.0
2.5
2.0
1.5
1.0
0.5
0.0
Chemical Shift (ppm)

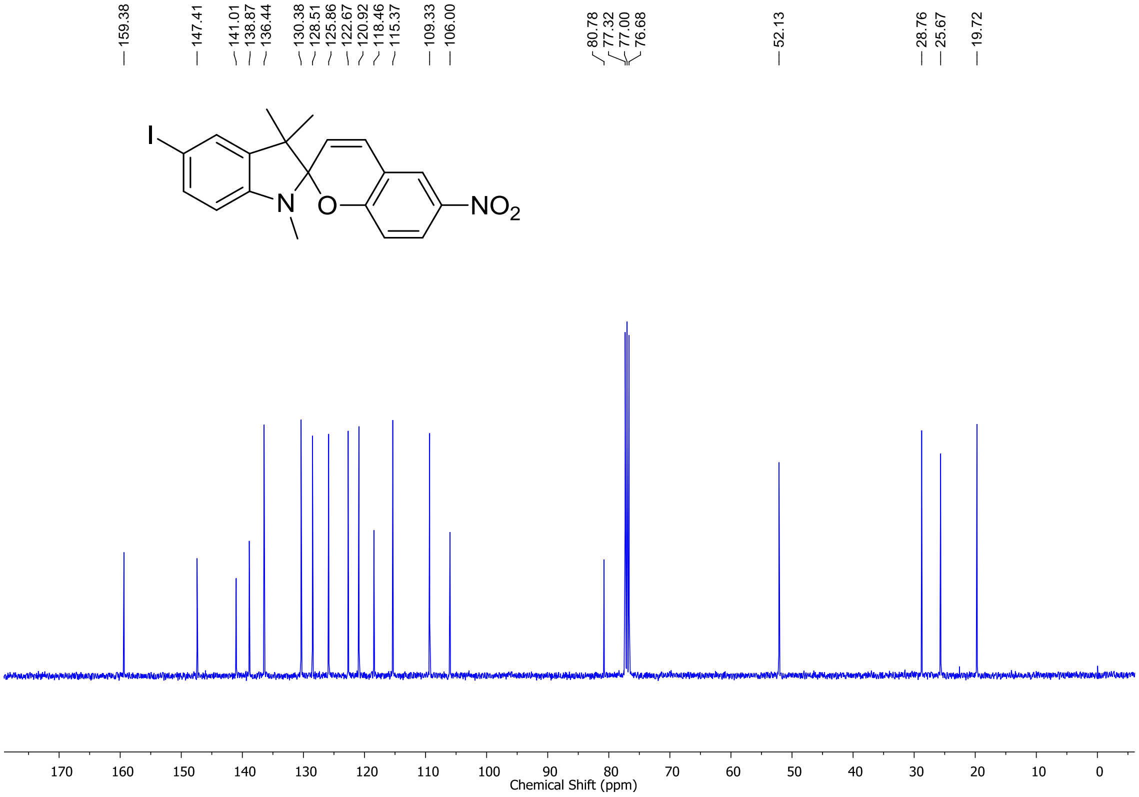

159.38
147.41
141.01
138.87
136.44
130.38
128.51
125.86
122.67
120.92
118.46
115.37
109.33
106.00
80.78
77.32
77.00
76.68
52.13
28.76
25.67
19.72
I
N
O
NO2
170
160
150
140
130
120
110
100
90
80
70
60
50
40
30
20
10
0
Chemical Shift (ppm)

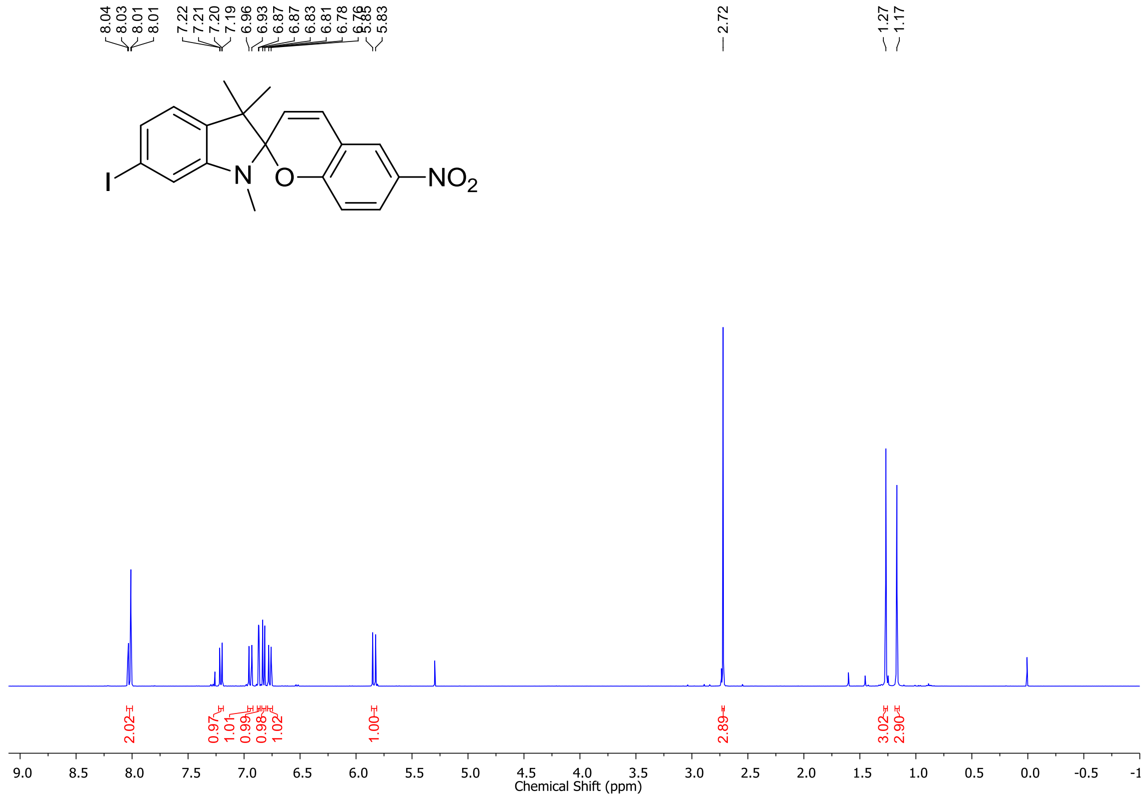

8.04
8.03
8.01
8.01
7.22
7.21
7.20
7.19
6.96
6.93
6.87
6.87
6.83
6.81
6.78
6.76
5.85
5.83
2.72
1.27
1.17
I
N
O
NO2
2.02
0.97
1.01
0.99
0.98
1.02
1.00
2.89
3.02
2.90
Chemical Shift (ppm)

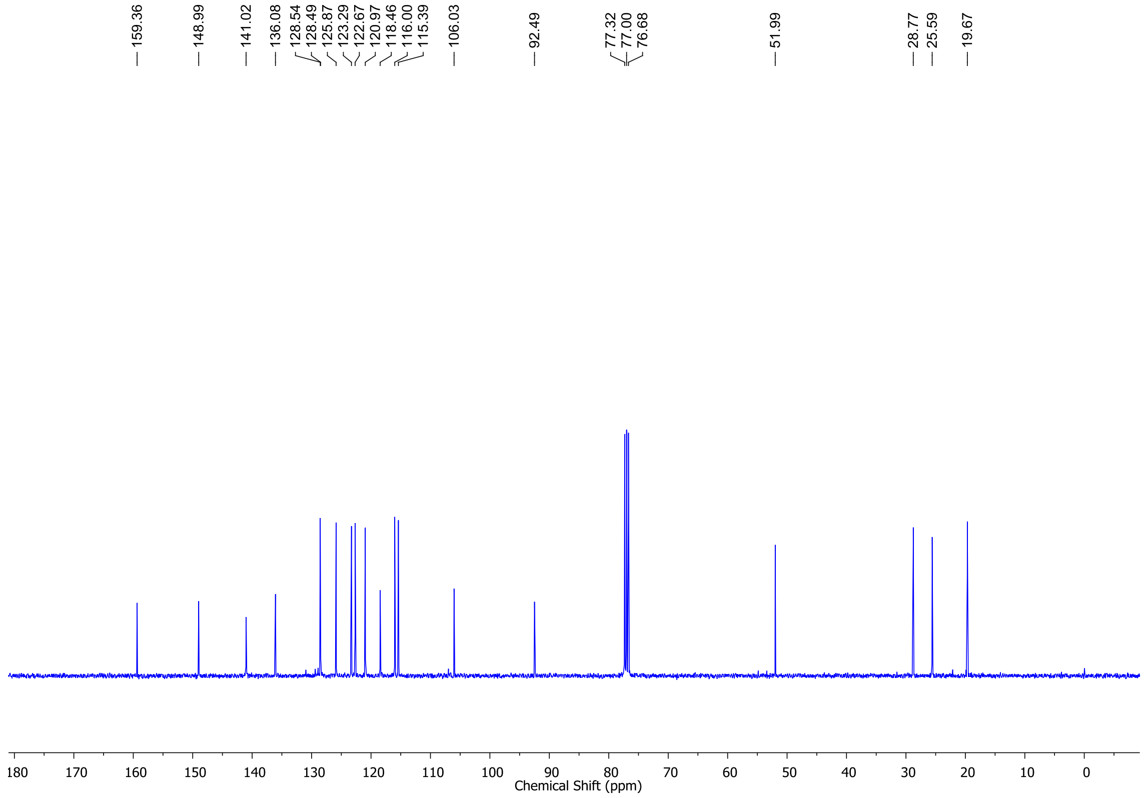

159.36
148.99
141.02
136.08
128.54
128.49
125.87
123.29
122.67
120.97
118.46
116.00
115.39
106.03
92.49
77.32
77.00
76.68
51.99
28.77
25.59
19.67
Chemical Shift (ppm)

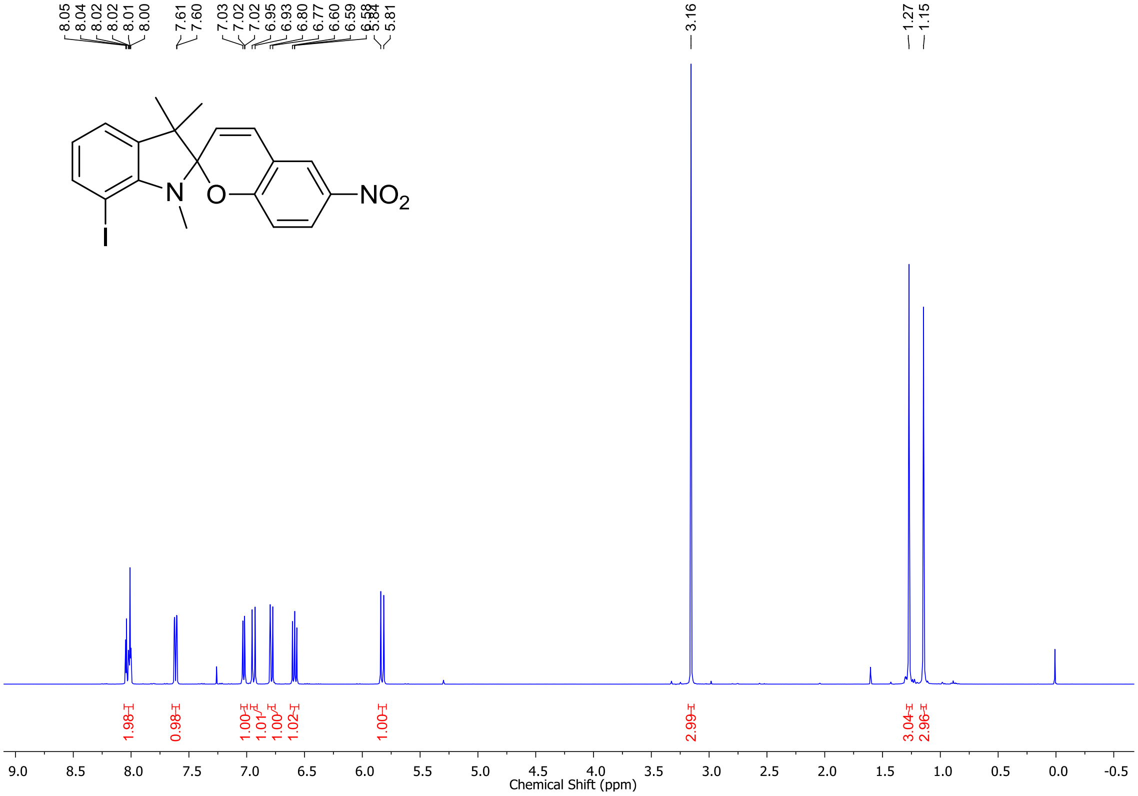

8.05
8.04
8.02
8.02
8.01
8.00
7.61
7.60
7.03
7.02
7.02
6.95
6.93
6.80
6.77
6.60
6.59
6.58
5.84
5.81
3.16
1.27
1.15
N
O
NO2
I
1.98
0.98
1.00
1.01
1.00
1.02
1.00
2.99
3.04
2.96
9.0
8.5
8.0
7.5
7.0
6.5
6.0
5.5
5.0
4.5
4.0
3.5
3.0
2.5
2.0
1.5
1.0
0.5
0.0
-0.5
Chemical Shift (ppm)

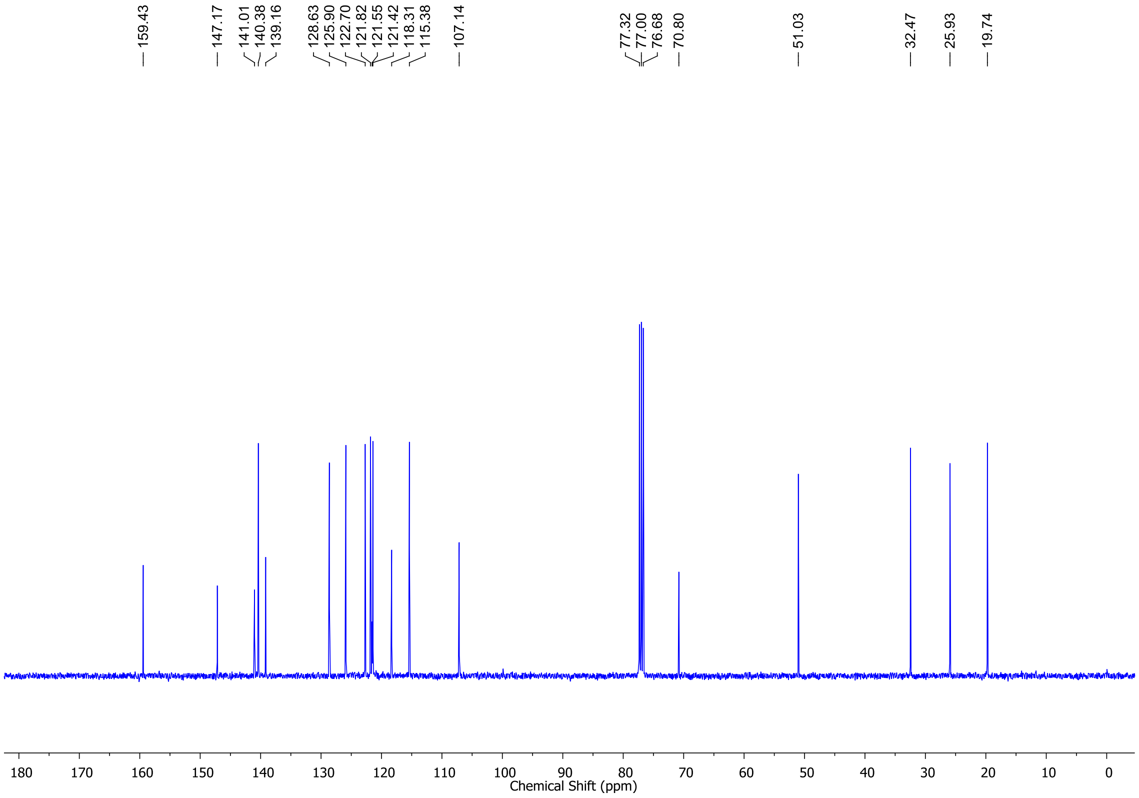

159.43
147.17
141.01
140.38
139.16
128.63
125.90
122.70
121.82
121.55
121.42
118.31
115.38
107.14
77.32
77.00
76.68
70.80
51.03
32.47
25.93
19.74
180
170
160
150
140
130
120
110
100
90
80
70
60
50
40
30
20
10
0
Chemical Shift (ppm)

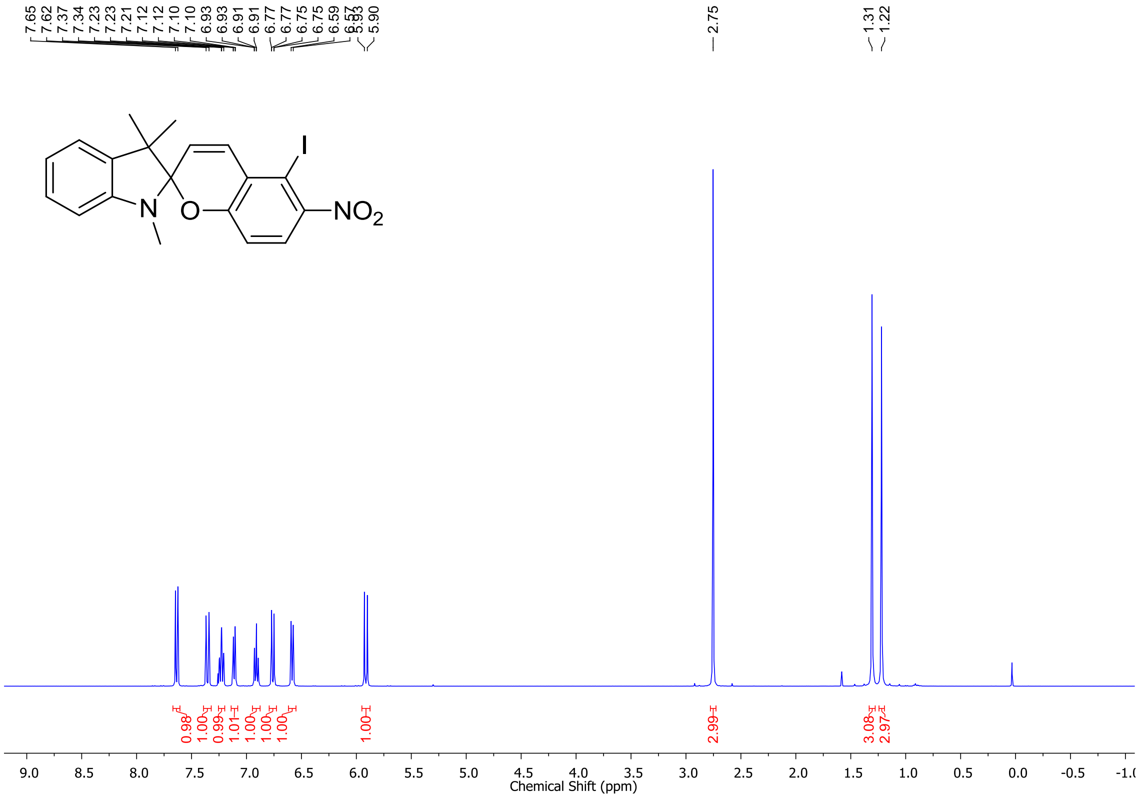

7.65
7.62
7.37
7.34
7.23
7.23
7.21
7.12
7.12
7.10
7.10
6.93
6.93
6.91
6.91
6.77
6.77
6.75
6.75
6.59
6.57
5.93
5.90
2.75
1.31
1.22
I
N
O
NO2
0.98
1.00
0.99
1.01
1.00
1.00
1.00
1.00
2.99
3.08
2.97
9.0
8.5
8.0
7.5
7.0
6.5
6.0
5.5
5.0
4.5
4.0
3.5
3.0
2.5
2.0
1.5
1.0
0.5
0.0
-0.5
Chemical Shift (ppm)

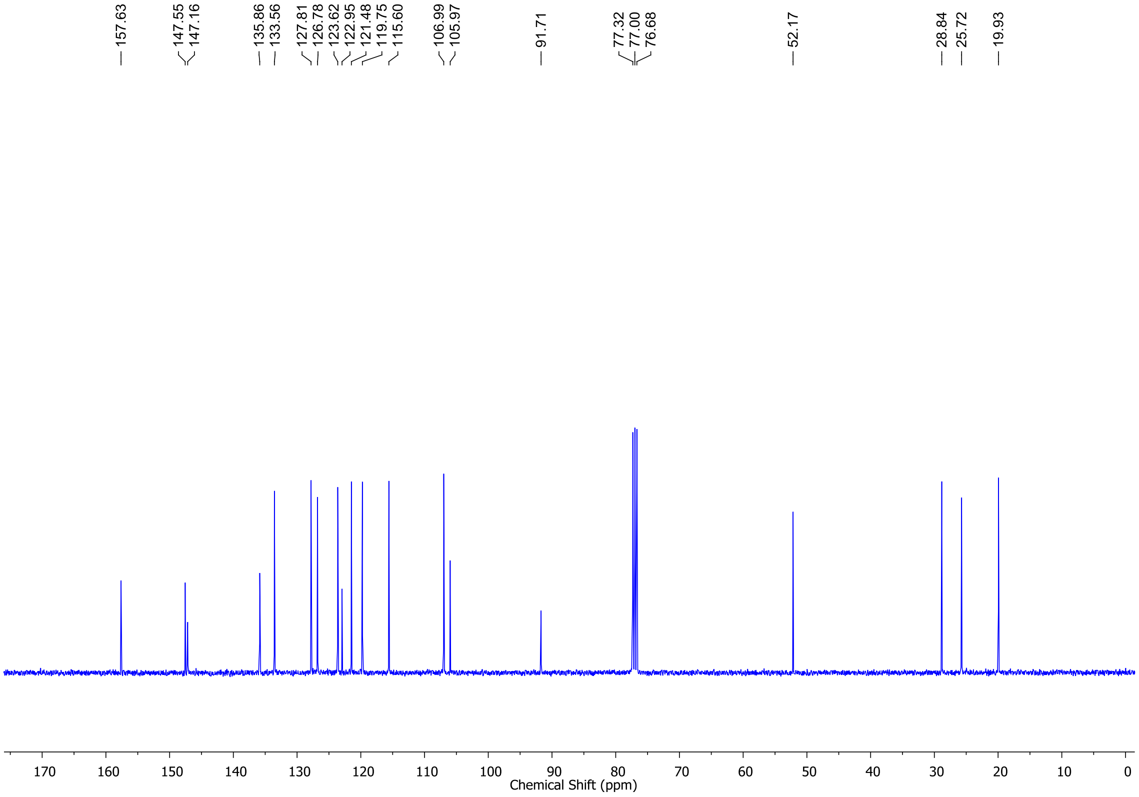

157.63
147.55
147.16
135.86
133.56
127.81
126.78
123.62
122.95
121.48
119.75
115.60
106.99
105.97
91.71
77.32
77.00
76.68
52.17
28.84
25.72
19.93
170
160
150
140
130
120
110
100
90
80
70
60
50
40
30
20
10
0
Chemical Shift (ppm)

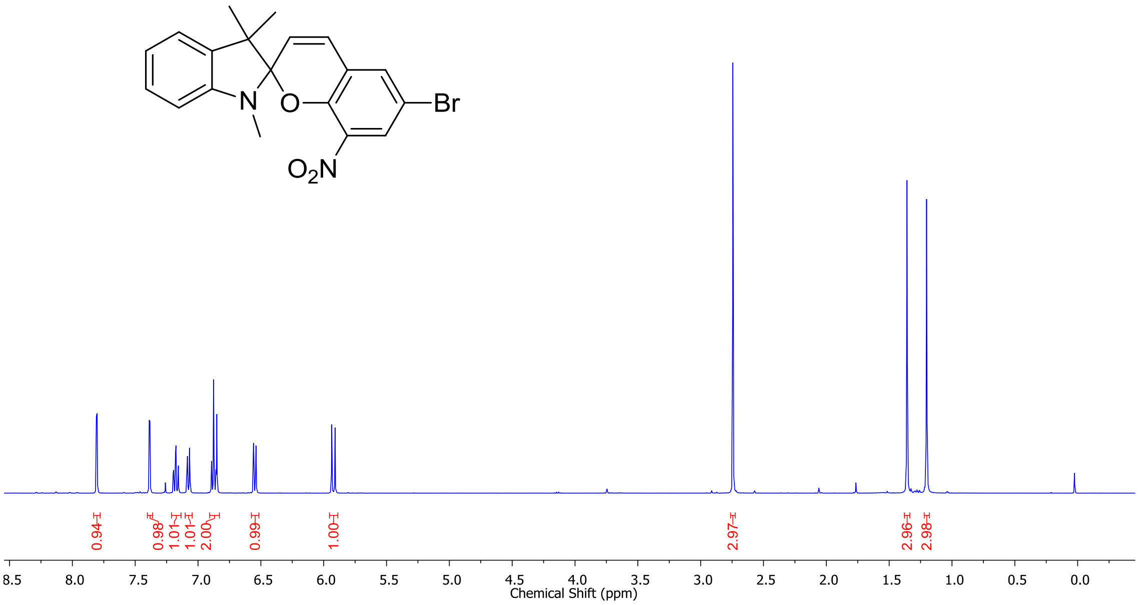
Br
N
O
O_2N
0.94
0.98
1.01
1.01
2.00
0.99
1.00
2.97
2.96
2.98
8.5 8.0 7.5 7.0 6.5 6.0 5.5 5.0 4.5 4.0 3.5 3.0 2.5 2.0 1.5 1.0 0.5 0.0
Chemical Shift (ppm)

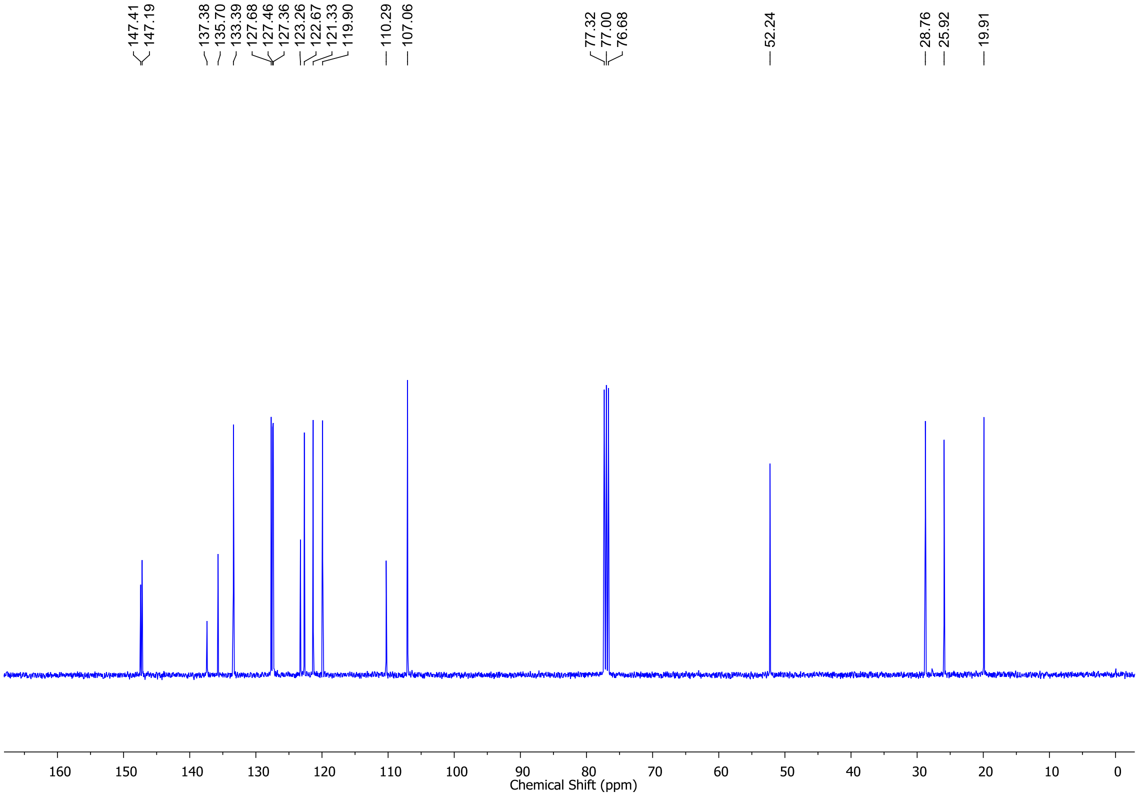
147.41
147.19
137.38
135.70
133.39
127.68
127.46
127.36
123.26
122.67
121.33
119.90
110.29
107.06
77.32
77.00
76.68
52.24
28.76
25.92
19.91
160 150 140 130 120 110 100 90 80 70 60 50 40 30 20 10 0
Chemical Shift (ppm)

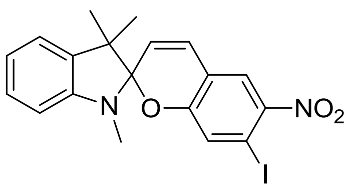
N
O
NO2
I

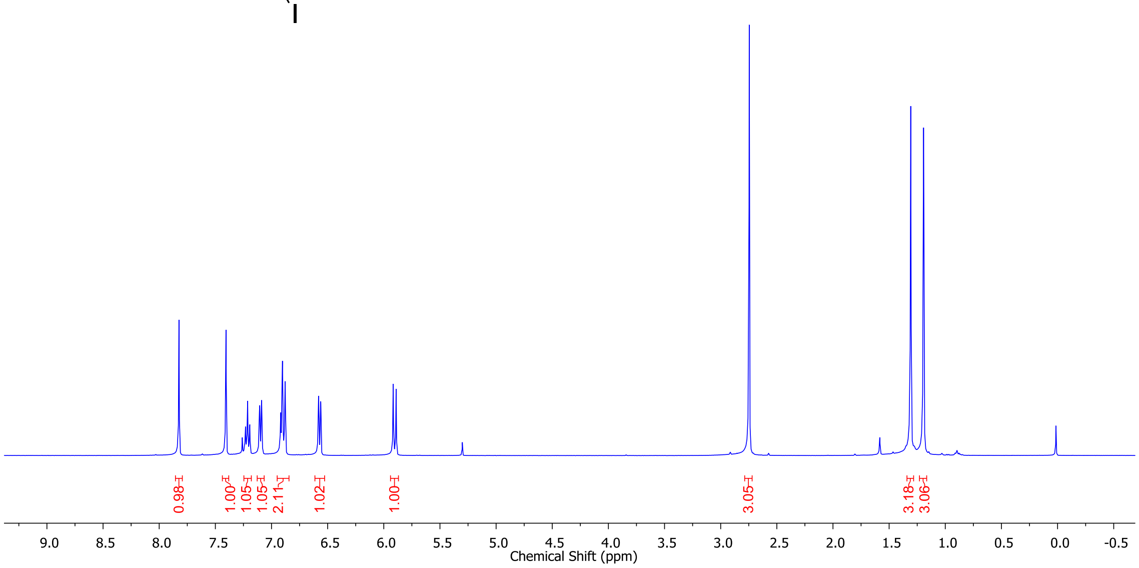
0.98
1.00
1.05
1.05
2.11
1.02
1.00
3.05
3.18
3.06
9.0 8.5 8.0 7.5 7.0 6.5 6.0 5.5 5.0 4.5 4.0 3.5 3.0 2.5 2.0 1.5 1.0 0.5 0.0 -0.5
Chemical Shift (ppm)

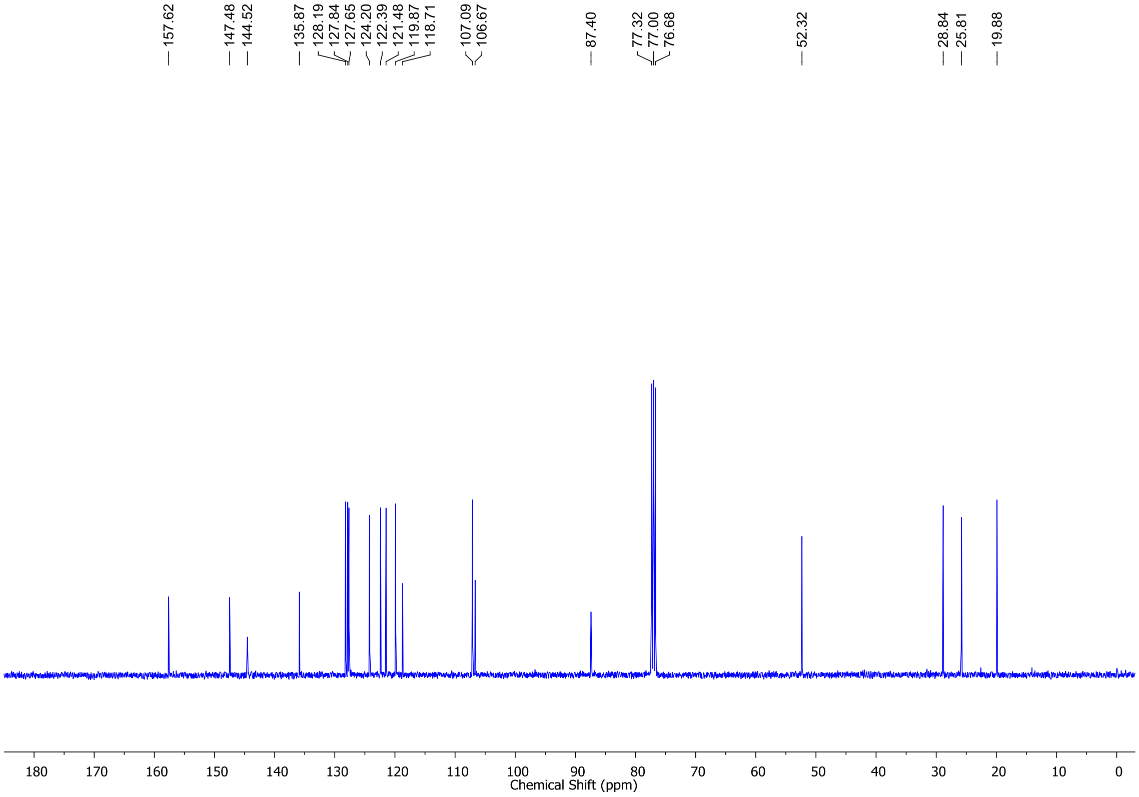
157.62
147.48
144.52
135.87
128.19
127.84
127.65
124.20
122.39
121.48
119.87
118.71
107.09
106.67
87.40
77.32
77.00
76.68
52.32
28.84
25.81
19.88
180 170 160 150 140 130 120 110 100 90 80 70 60 50 40 30 20 10 0
Chemical Shift (ppm)

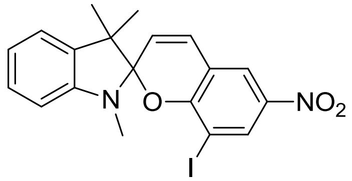

N
O
NO2
I

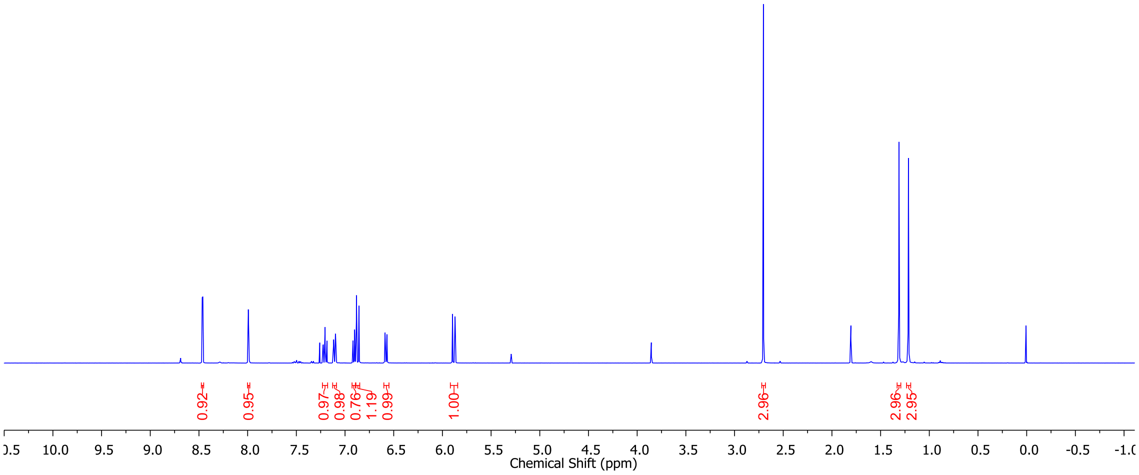

0.92
0.95
0.97
0.98
0.76
1.19
0.99
1.00
2.96
2.96
2.95
0.5
10.0
9.5
9.0
8.5
8.0
7.5
7.0
6.5
6.0
5.5
5.0
4.5
4.0
3.5
3.0
2.5
2.0
1.5
1.0
0.5
0.0
-0.5
-1.0
Chemical Shift (ppm)

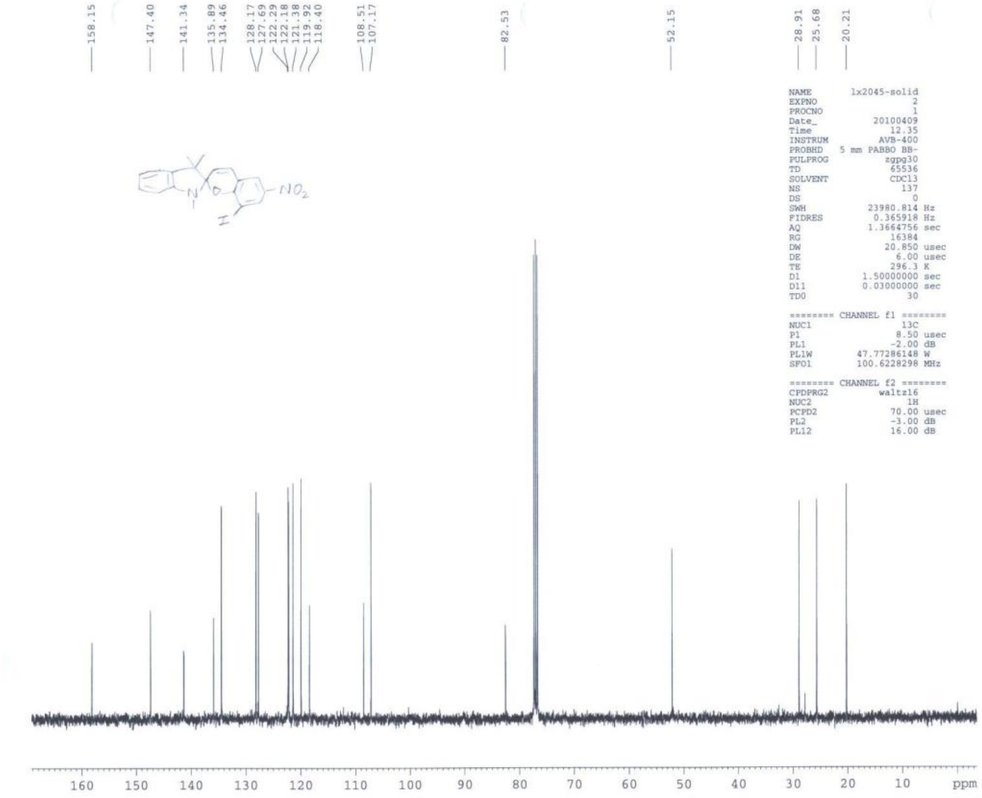

158.15
147.40
141.34
135.89
134.46
128.17
127.69
122.29
122.18
121.38
119.92
118.40
108.51
107.17
82.53
52.15
28.91
25.68
20.21
NAME 1x2045-solid
EXPNO 2
PROCNO 1
Date_ 20100409
Time 12.35
INSTRUM AVB-400
PROBHD 5 mm PABBO BB-
PULPROG zgpg30
TD 65536
SOLVENT CDCl3
NS 137
DS 0
SWH 23980.814 Hz
FIDRES 0.365918 Hz
AQ 1.3664756 sec
RG 16384
DW 20.850 usec
DE 6.00 usec
TE 296.3 K
D1 1.50000000 sec
D11 0.03000000 sec
TD0 30
======== CHANNEL f1 ========
NUC1 13C
P1 8.50 usec
PL1 -2.00 dB
PL1W 47.77286148 W
SFO1 100.6228298 MHz
======== CHANNEL f2 ========
CPDPRG2 waltz16
NUC2 1H
PCPD2 70.00 usec
PL2 -3.00 dB
PL12 16.00 dB
160
150
140
130
120
110
100
90
80
70
60
50
40
30
20
10
ppm

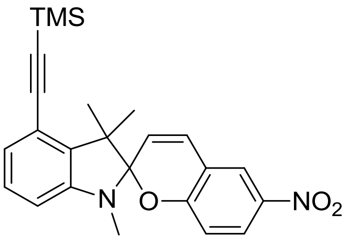
TMS
N
O
NO2

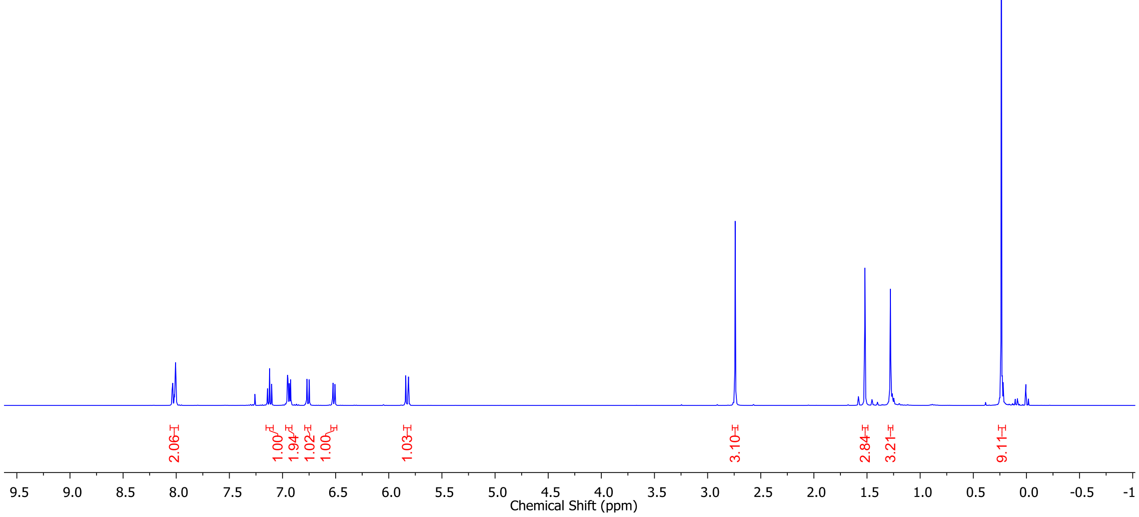
2.06
1.00
1.94
1.02
1.00
1.03
3.10
2.84
3.21
9.11
9.5 9.0 8.5 8.0 7.5 7.0 6.5 6.0 5.5 5.0 4.5 4.0 3.5 3.0 2.5 2.0 1.5 1.0 0.5 0.0 -0.5 -1
Chemical Shift (ppm)

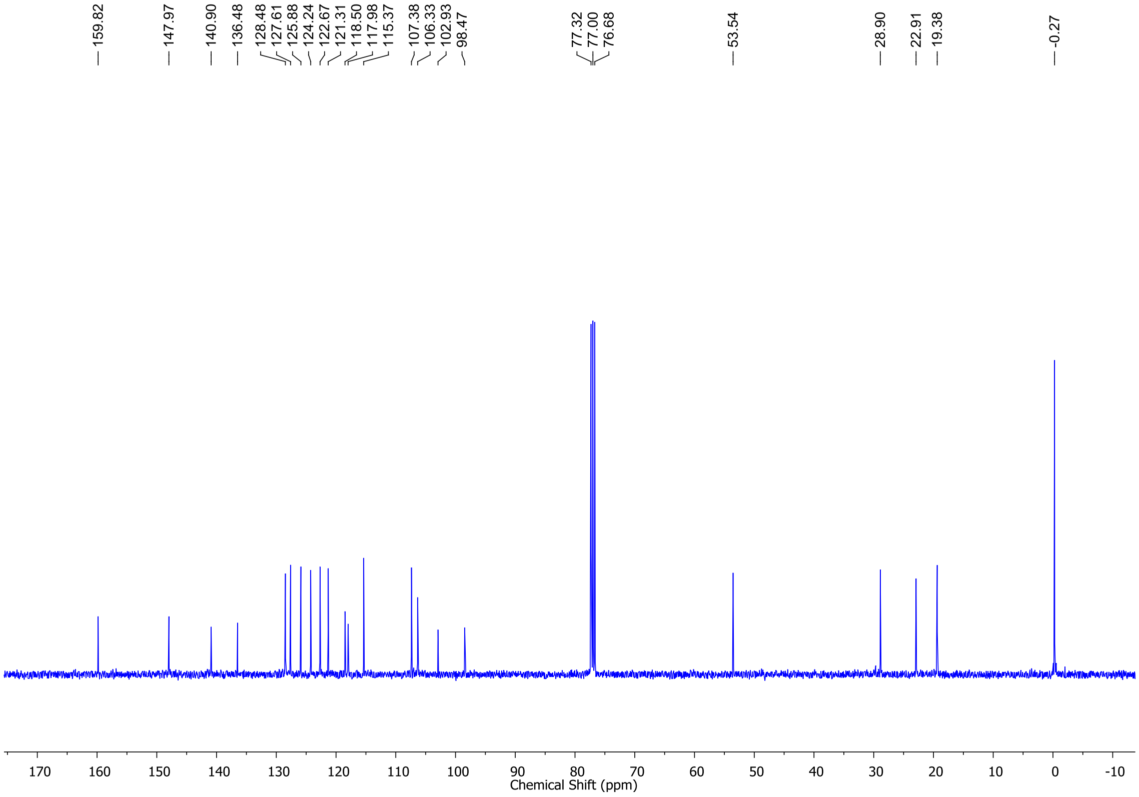
159.82
147.97
140.90
136.48
128.48
127.61
125.88
124.24
122.67
121.31
118.50
117.98
115.37
107.38
106.33
102.93
98.47
77.32
77.00
76.68
53.54
28.90
22.91
19.38
-0.27
170 160 150 140 130 120 110 100 90 80 70 60 50 40 30 20 10 0 -10
Chemical Shift (ppm)

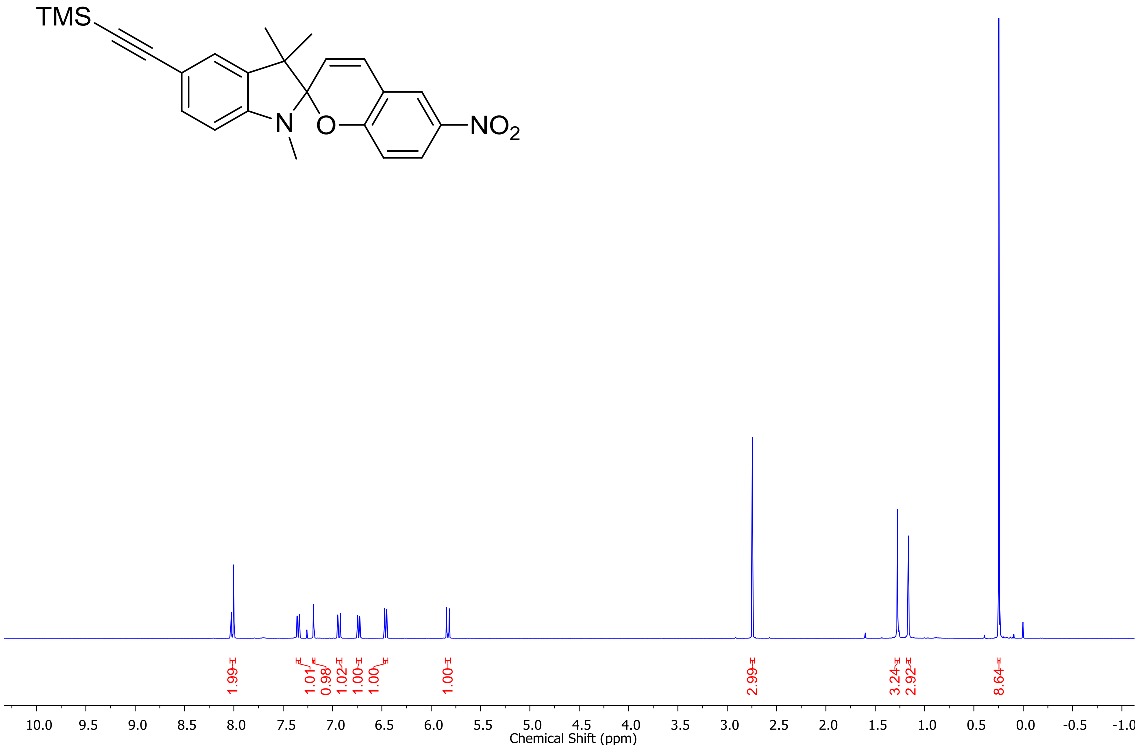
TMS
N
O
NO2
1.99
1.01
0.98
1.02
1.00
1.00
1.00
2.99
3.24
2.92
8.64
Chemical Shift (ppm)

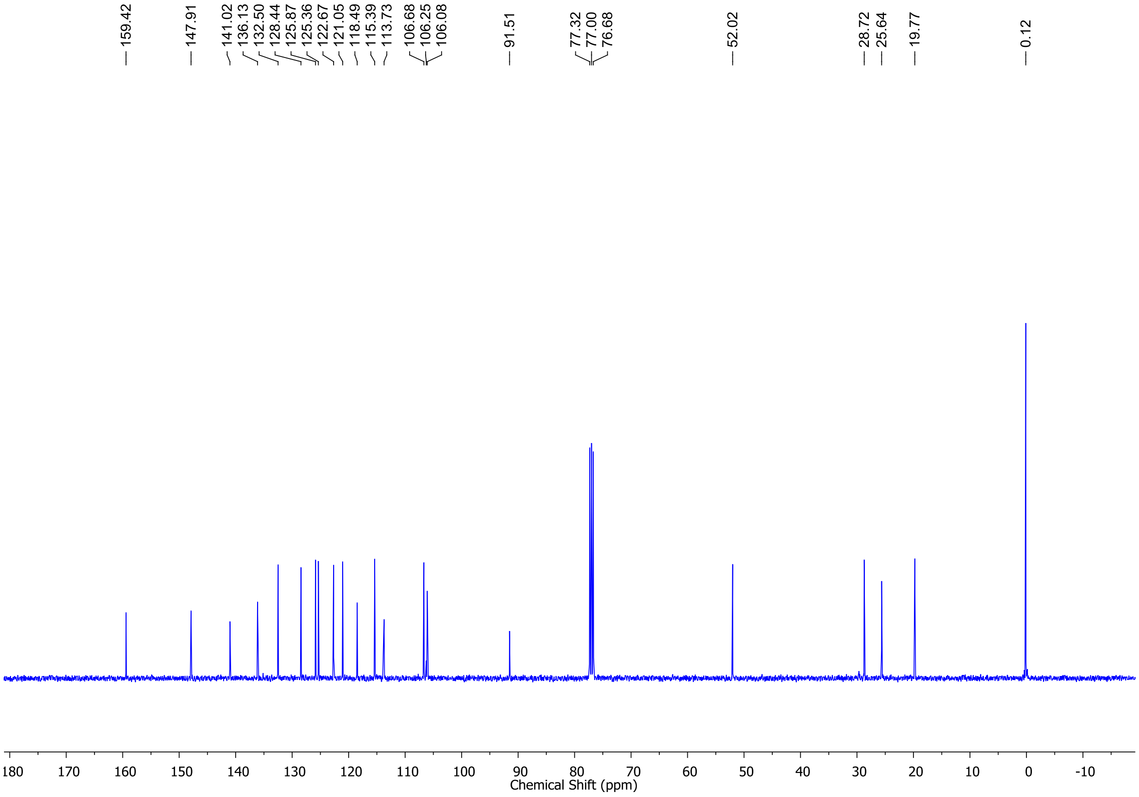
159.42
147.91
141.02
136.13
132.50
128.44
125.87
125.36
122.67
121.05
118.49
115.39
113.73
106.68
106.25
106.08
91.51
77.32
77.00
76.68
52.02
28.72
25.64
19.77
0.12
Chemical Shift (ppm)

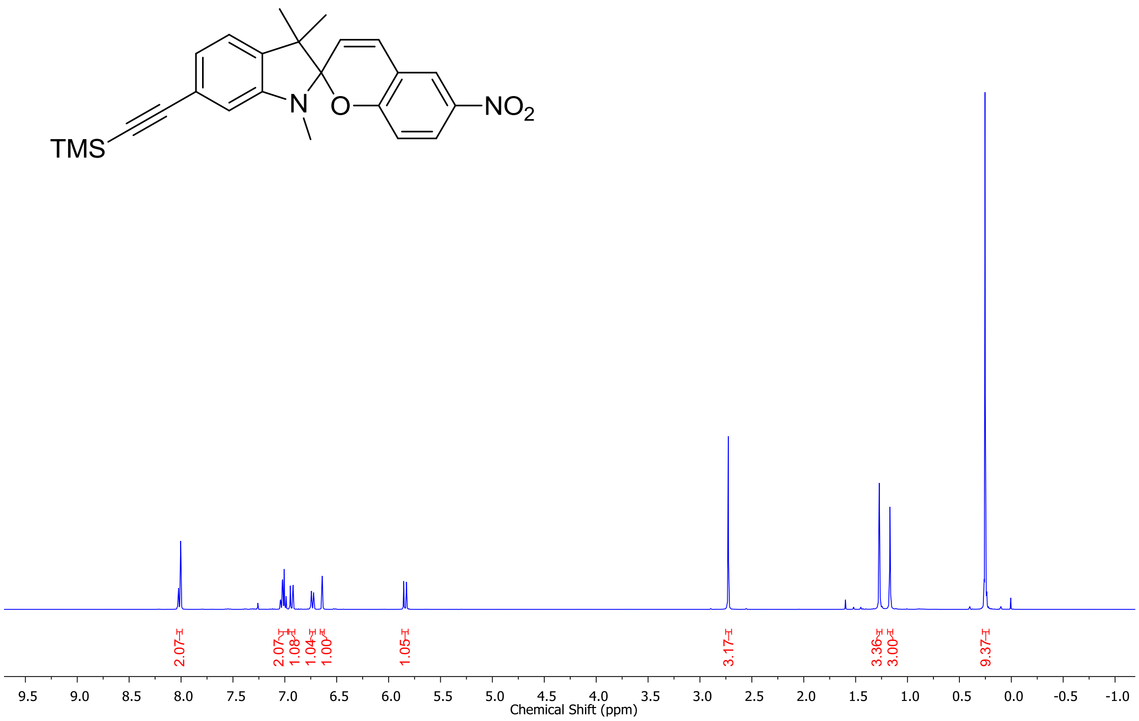
TMS
N
O
NO_2
2.07
2.07
1.08
1.04
1.00
1.05
3.17
3.36
3.00
9.37
9.5 9.0 8.5 8.0 7.5 7.0 6.5 6.0 5.5 5.0 4.5 4.0 3.5 3.0 2.5 2.0 1.5 1.0 0.5 0.0 -0.5 -1.0
Chemical Shift (ppm)

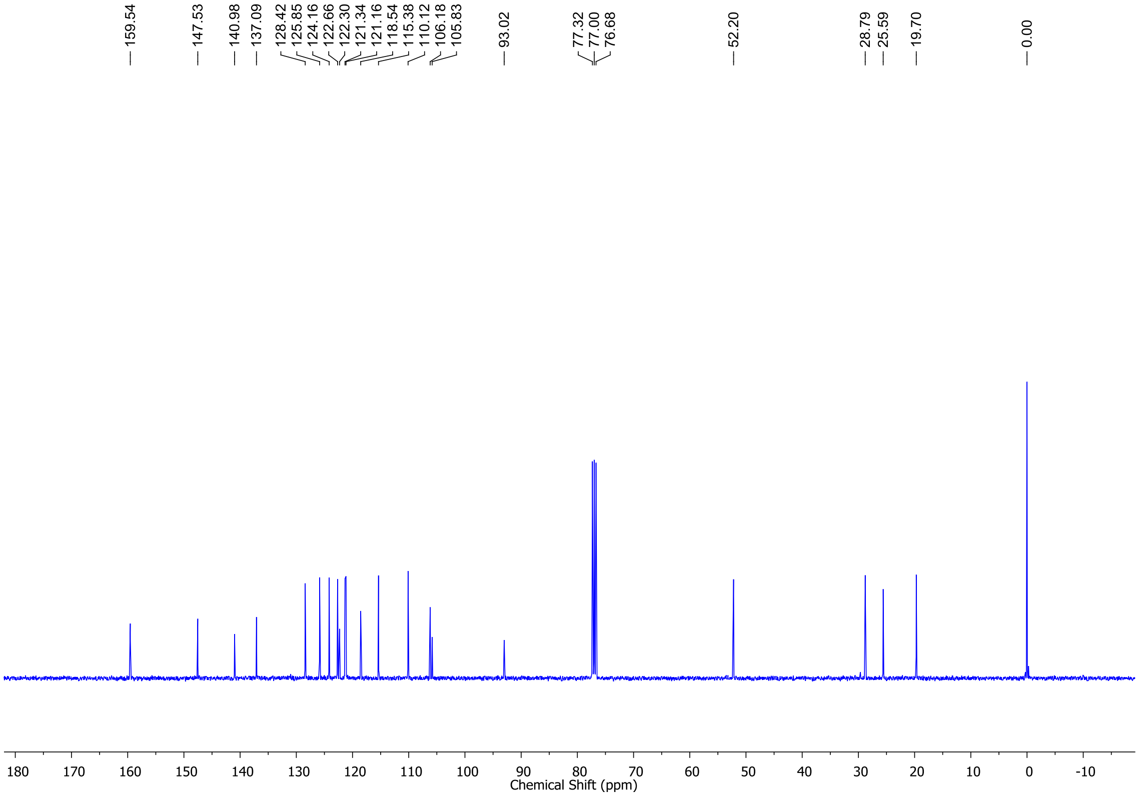
159.54
147.53
140.98
137.09
128.42
125.85
124.16
122.66
122.30
121.34
121.16
118.54
115.38
110.12
106.18
105.83
93.02
77.32
77.00
76.68
52.20
28.79
25.59
19.70
0.00
180 170 160 150 140 130 120 110 100 90 80 70 60 50 40 30 20 10 0 -10
Chemical Shift (ppm)

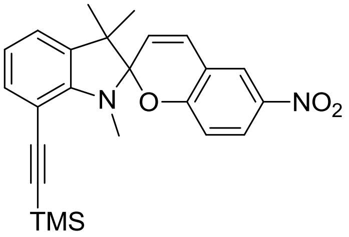
N
O
NO2
TMS

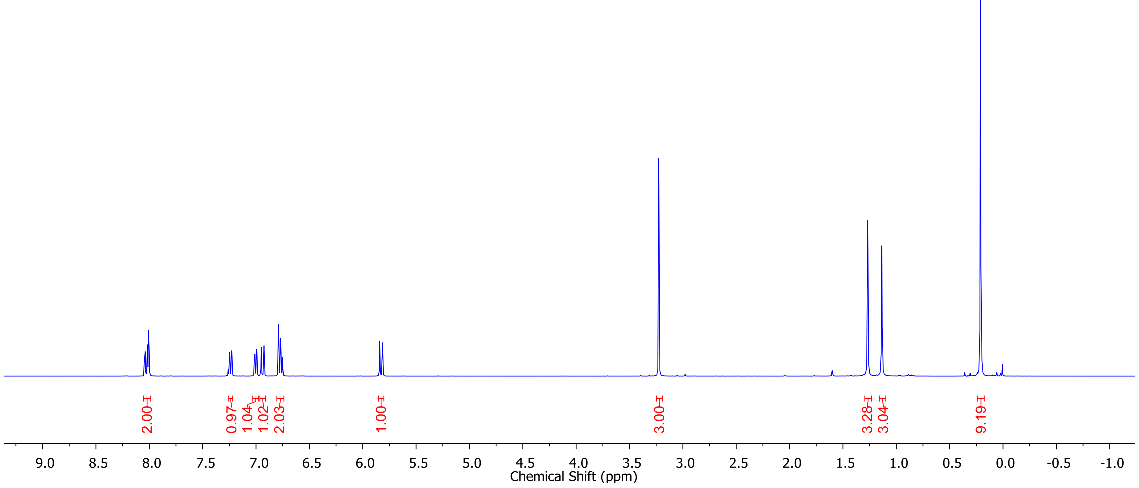
2.00
0.97
1.04
1.02
2.03
1.00
3.00
3.28
3.04
9.19
9.0 8.5 8.0 7.5 7.0 6.5 6.0 5.5 5.0 4.5 4.0 3.5 3.0 2.5 2.0 1.5 1.0 0.5 0.0 -0.5 -1.0
Chemical Shift (ppm)

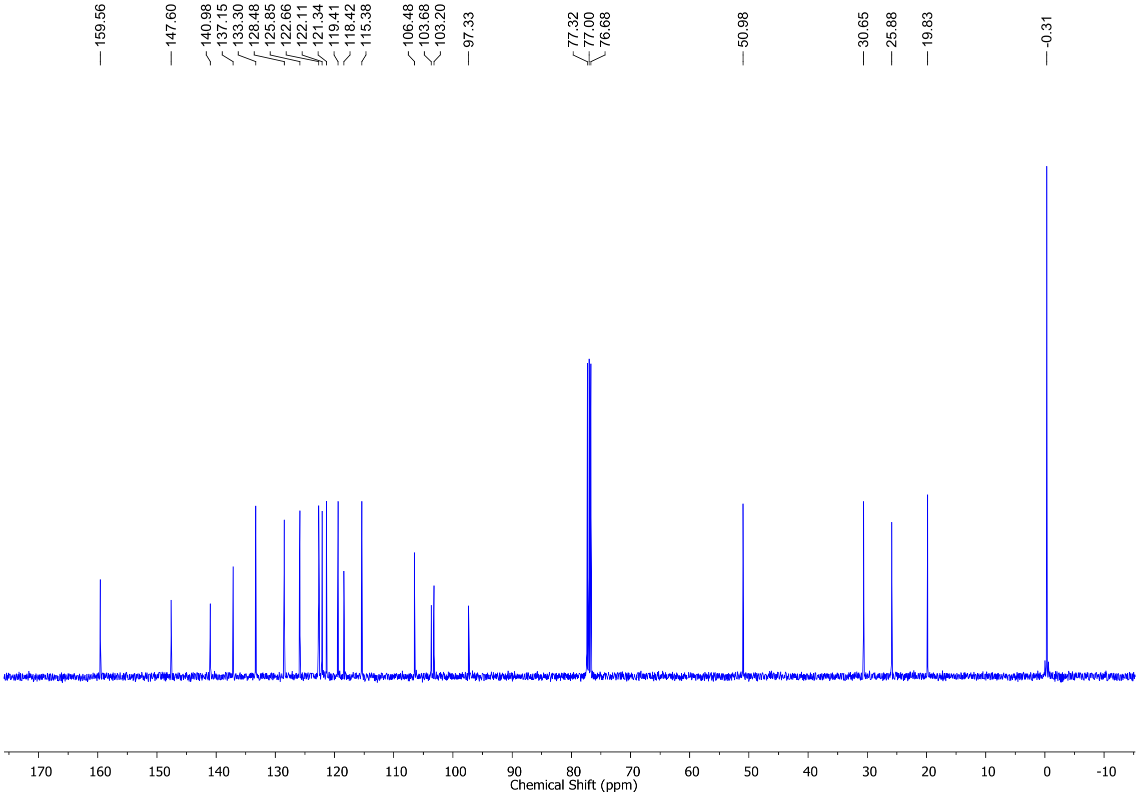
159.56
147.60
140.98
137.15
133.30
128.48
125.85
122.66
122.11
121.34
119.41
118.42
115.38
106.48
103.68
103.20
97.33
77.32
77.00
76.68
50.98
30.65
25.88
19.83
-0.31
170 160 150 140 130 120 110 100 90 80 70 60 50 40 30 20 10 0 -10
Chemical Shift (ppm)

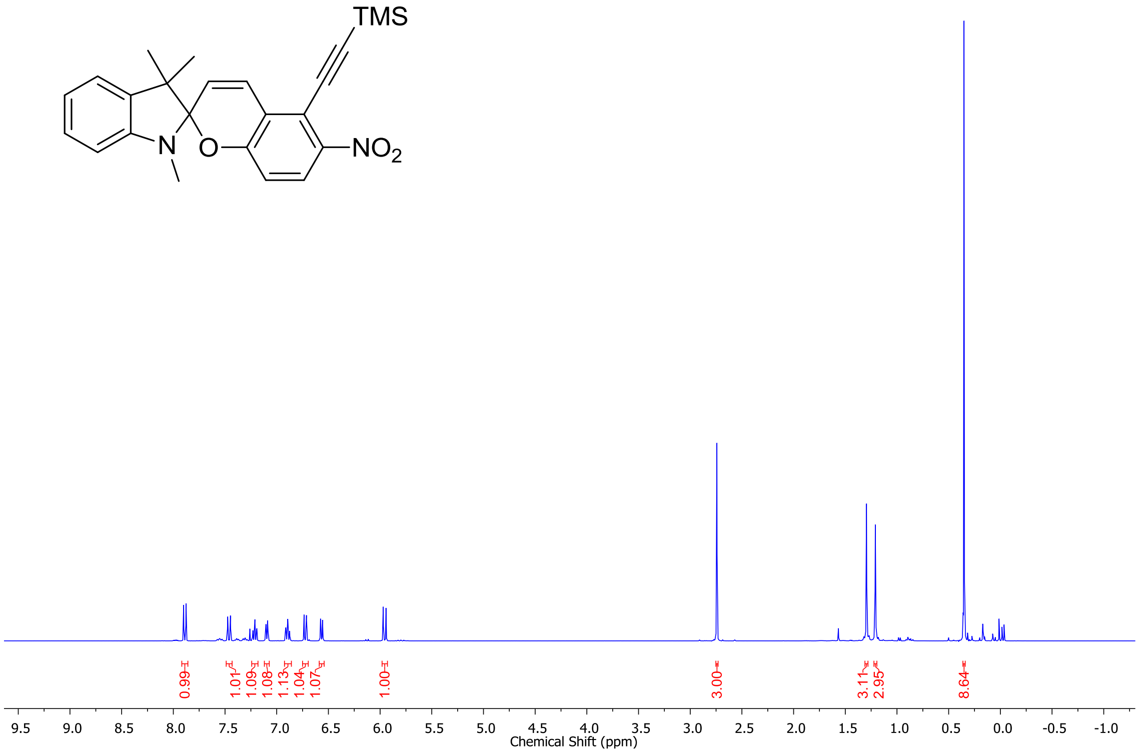
TMS
N
O
NO2
0.99
1.01
1.09
1.08
1.13
1.04
1.07
1.00
3.00
3.11
2.95
8.64
9.5 9.0 8.5 8.0 7.5 7.0 6.5 6.0 5.5 5.0 4.5 4.0 3.5 3.0 2.5 2.0 1.5 1.0 0.5 0.0 -0.5 -1.0
Chemical Shift (ppm)

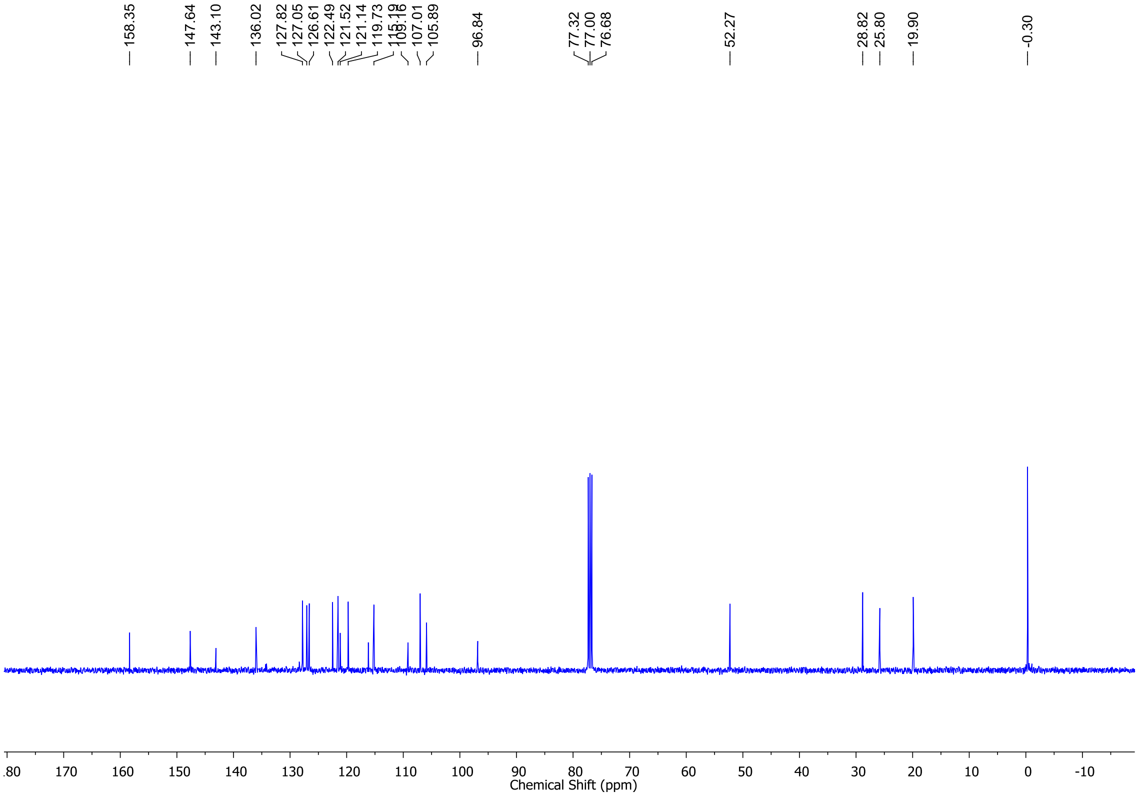
158.35
147.64
143.10
136.02
127.82
127.05
126.61
122.49
121.52
121.14
119.73
115.19
109.16
107.01
105.89
96.84
77.32
77.00
76.68
52.27
28.82
25.80
19.90
-0.30
.80 170 160 150 140 130 120 110 100 90 80 70 60 50 40 30 20 10 0 -10
Chemical Shift (ppm)

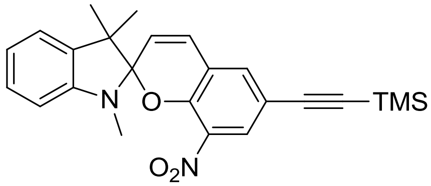

O2N
N
O
TMS

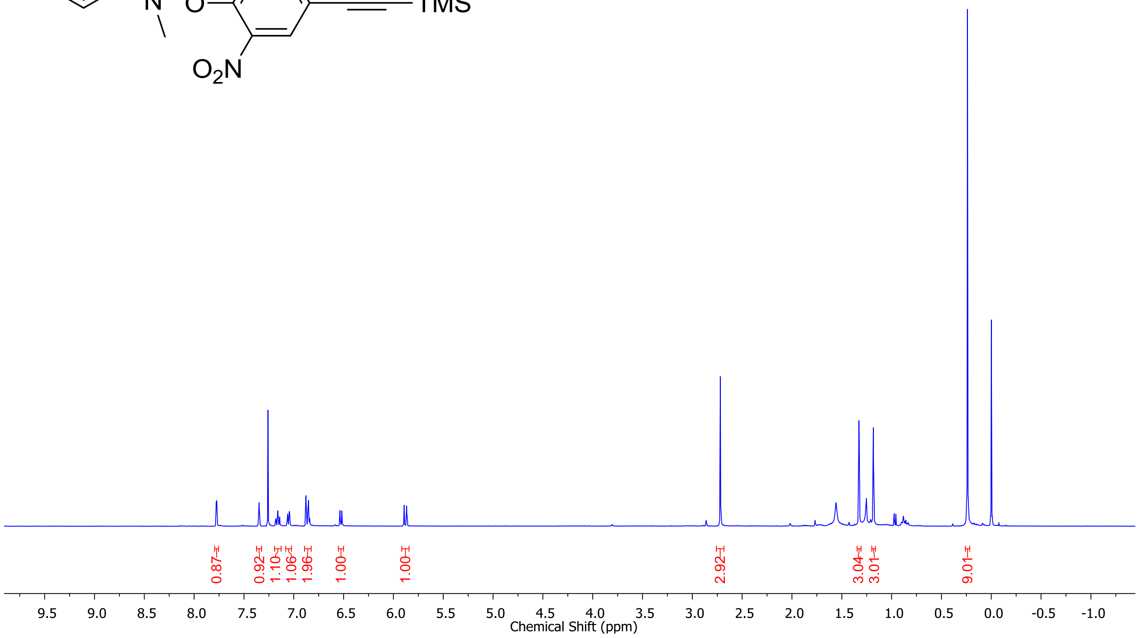

0.87
0.92
1.10
1.06
1.96
1.00
1.00
2.92
3.04
3.01
9.01
9.5 9.0 8.5 8.0 7.5 7.0 6.5 6.0 5.5 5.0 4.5 4.0 3.5 3.0 2.5 2.0 1.5 1.0 0.5 0.0 -0.5 -1.0
Chemical Shift (ppm)

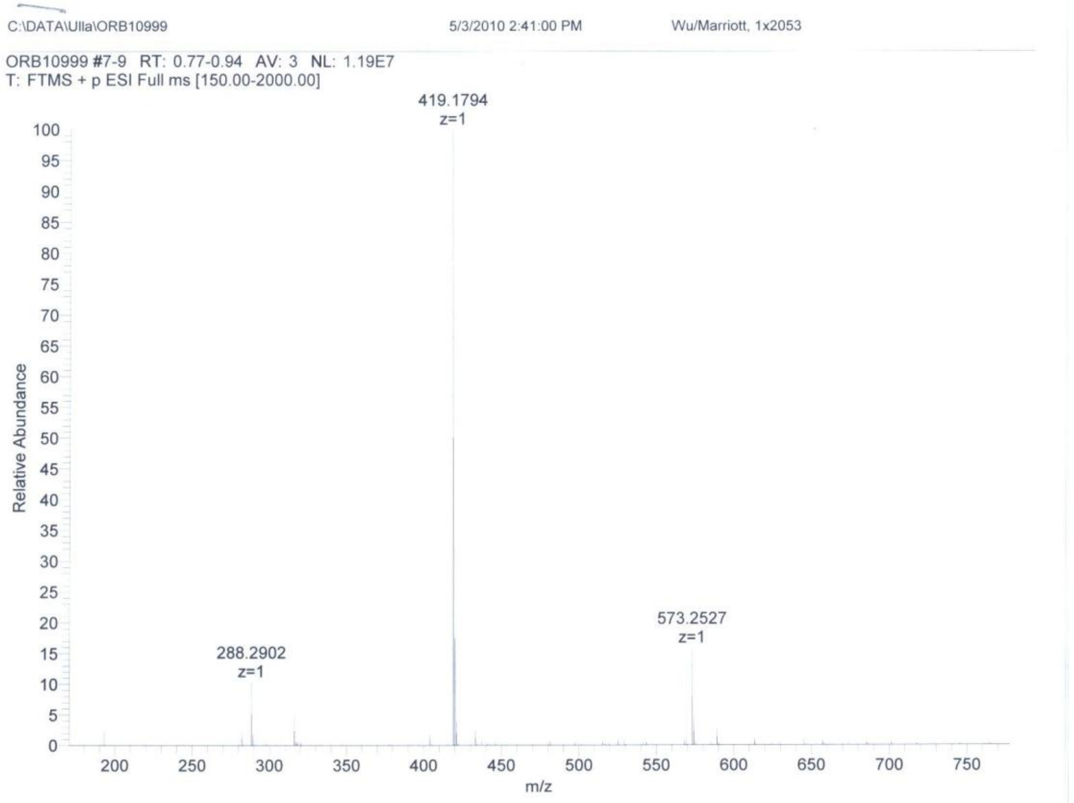

C:\DATA\Ulla\ORB10999
5/3/2010 2:41:00 PM
Wu/Marriott, 1x2053
ORB10999 #7-9 RT: 0.77-0.94 AV: 3 NL: 1.19E7
T: FTMS + p ESI Full ms [150.00-2000.00]
419.1794
z=1
573.2527
z=1
288.2902
z=1
Relative Abundance
m/z

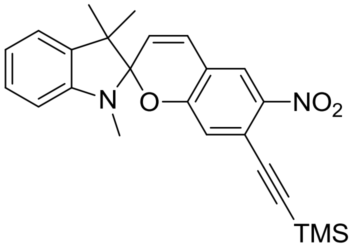
N
O
NO2
TMS

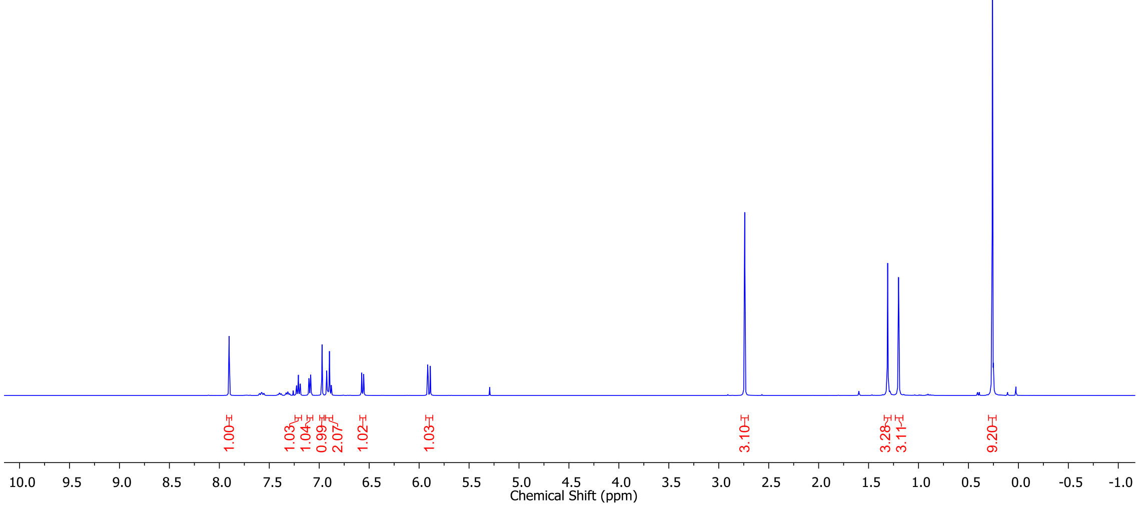
1.00
1.03
1.04
0.99
2.07
1.02
1.03
3.10
3.28
3.11
9.20
10.0 9.5 9.0 8.5 8.0 7.5 7.0 6.5 6.0 5.5 5.0 4.5 4.0 3.5 3.0 2.5 2.0 1.5 1.0 0.5 0.0 -0.5 -1.0
Chemical Shift (ppm)

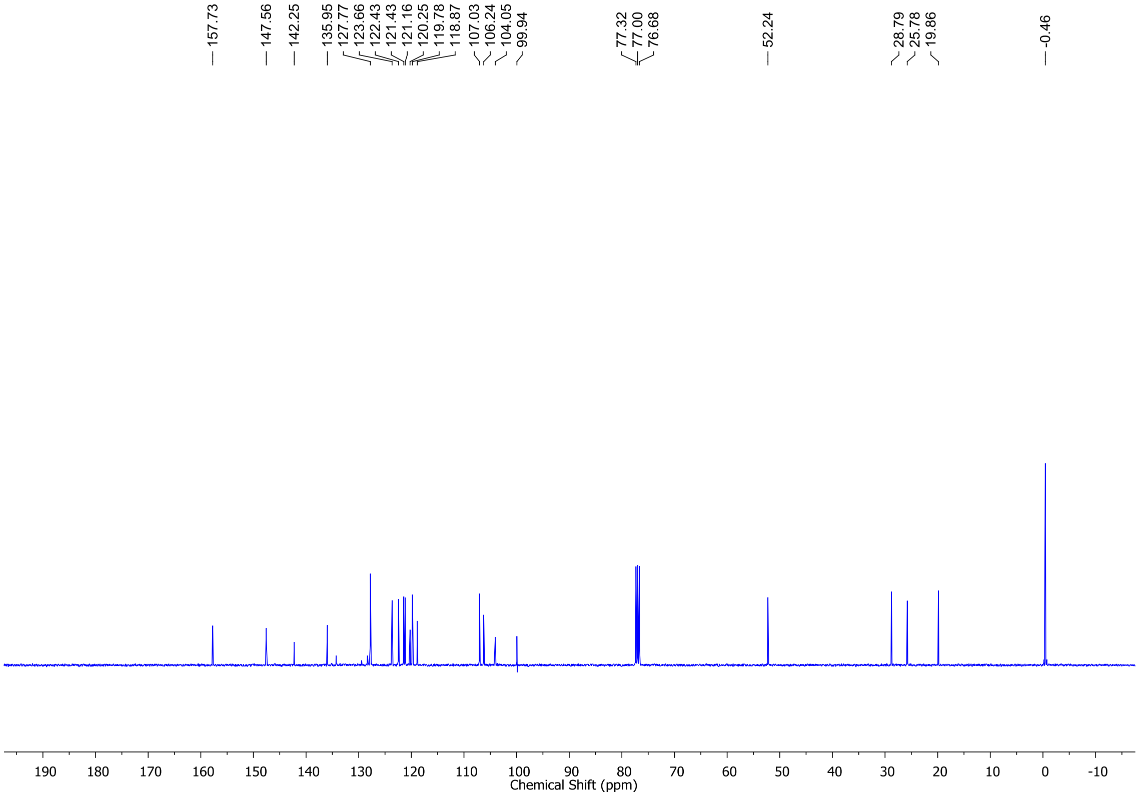
157.73
147.56
142.25
135.95
127.77
123.66
122.43
121.43
121.16
120.25
119.78
118.87
107.03
106.24
104.05
99.94
77.32
77.00
76.68
52.24
28.79
25.78
19.86
-0.46
190 180 170 160 150 140 130 120 110 100 90 80 70 60 50 40 30 20 10 0 -10
Chemical Shift (ppm)

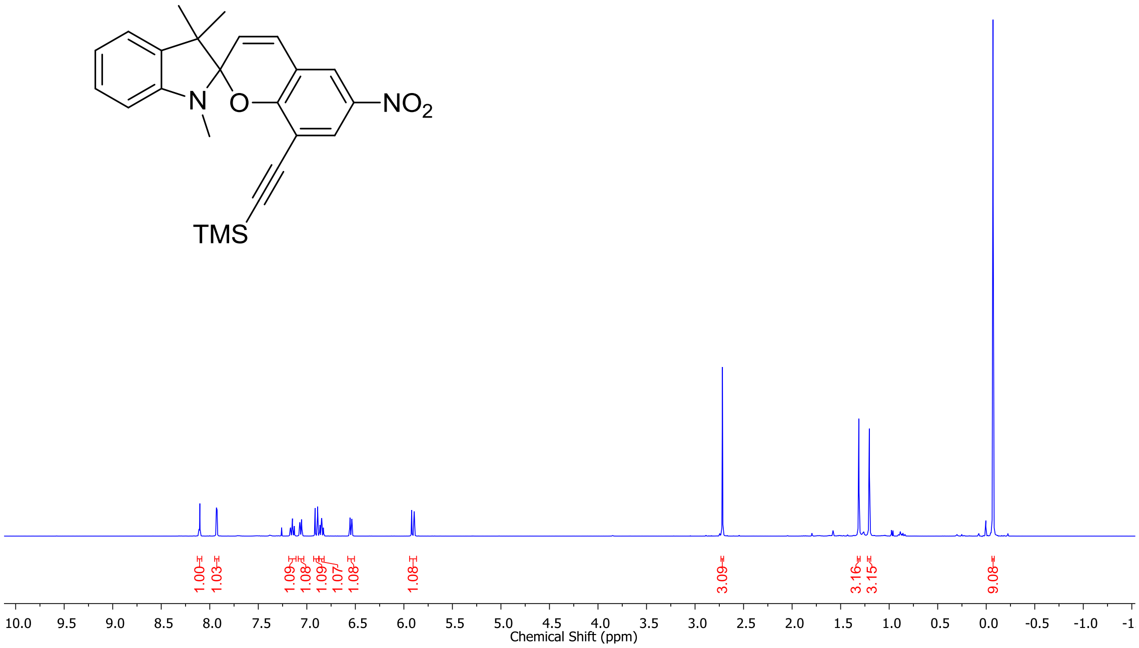
NO2
N
O
TMS
1.00
1.03
1.09
1.08
1.09
1.07
1.08
1.08
3.09
3.16
3.15
9.08
10.0 9.5 9.0 8.5 8.0 7.5 7.0 6.5 6.0 5.5 5.0 4.5 4.0 3.5 3.0 2.5 2.0 1.5 1.0 0.5 0.0 -0.5 -1.0 -1
Chemical Shift (ppm)

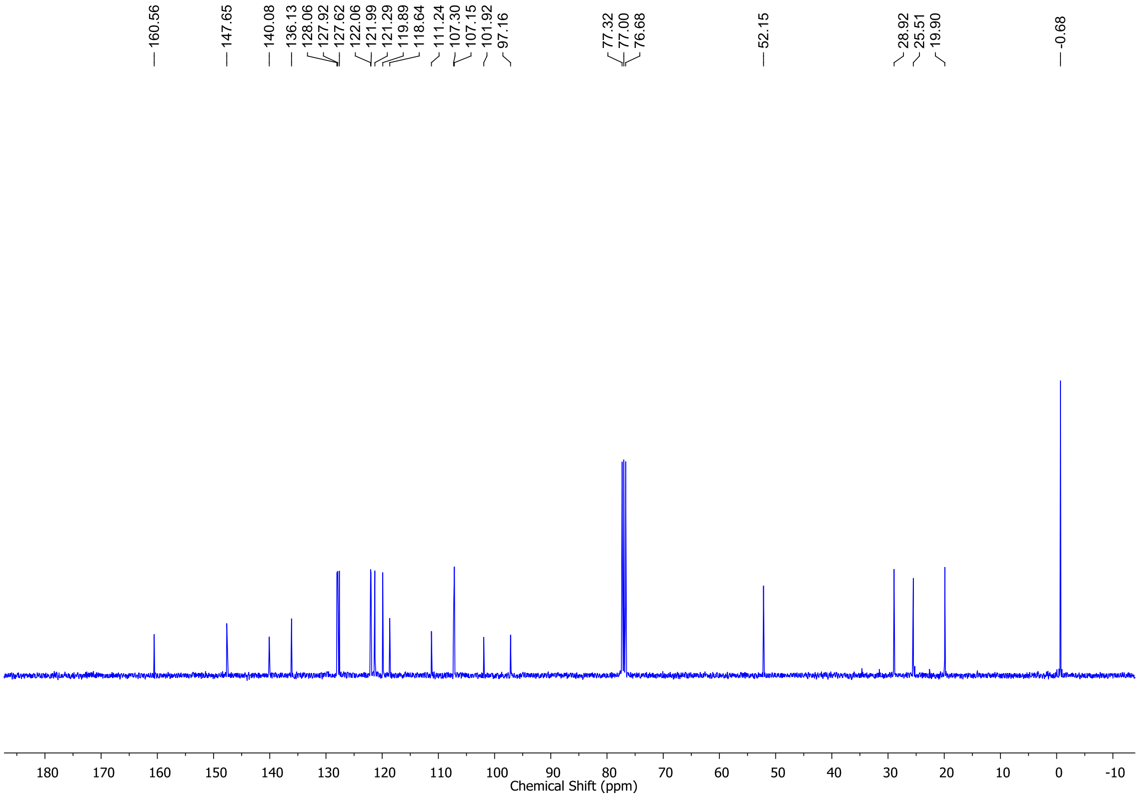
160.56
147.65
140.08
136.13
128.06
127.92
127.62
122.06
121.99
121.29
119.89
118.64
111.24
107.30
107.15
101.92
97.16
77.32
77.00
76.68
52.15
28.92
25.51
19.90
-0.68
180 170 160 150 140 130 120 110 100 90 80 70 60 50 40 30 20 10 0 -10
Chemical Shift (ppm)

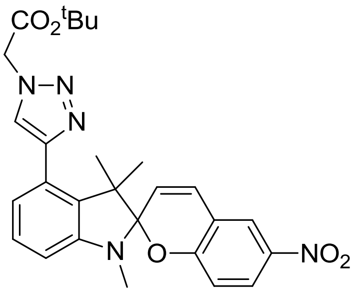
CO2tBu
N
N
N
N
O
NO2

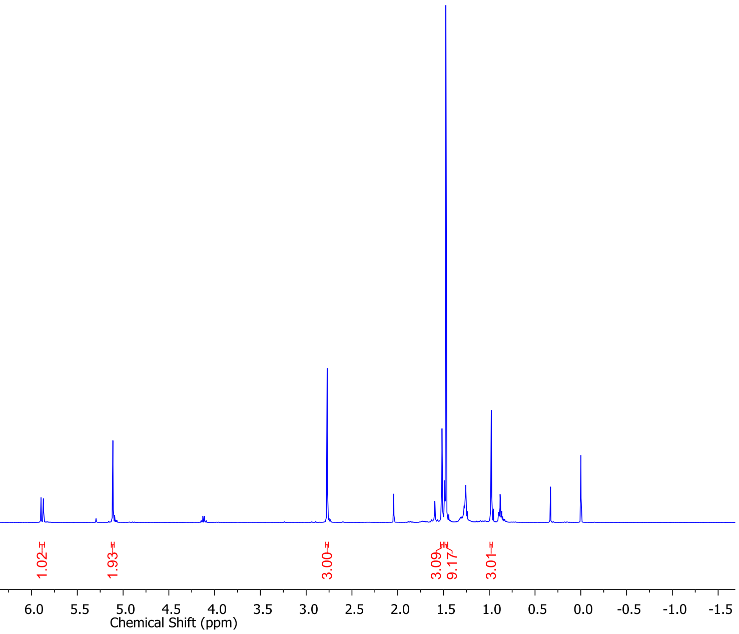
2.10
1.00
0.99
1.06
1.01
1.02
1.02
1.02
1.93
3.00
3.09
9.17
3.01
10.5 10.0 9.5 9.0 8.5 8.0 7.5 7.0 6.5 6.0 5.5 5.0 4.5 4.0 3.5 3.0 2.5 2.0 1.5 1.0 0.5 0.0 -0.5 -1.0 -1.5
Chemical Shift (ppm)

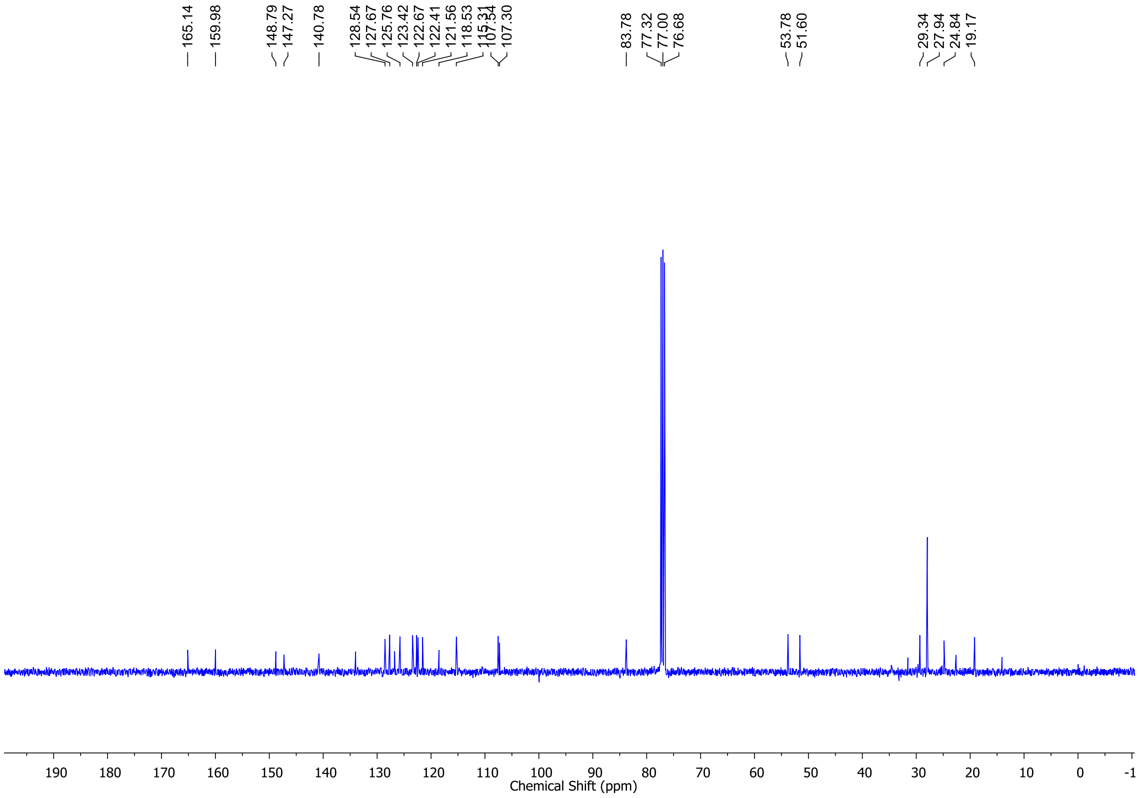
165.14
159.98
148.79
147.27
140.78
128.54
127.67
125.76
123.42
122.67
122.41
121.56
118.53
115.31
107.54
107.30
83.78
77.32
77.00
76.68
53.78
51.60
29.34
27.94
24.84
19.17
190 180 170 160 150 140 130 120 110 100 90 80 70 60 50 40 30 20 10 0 -1
Chemical Shift (ppm)

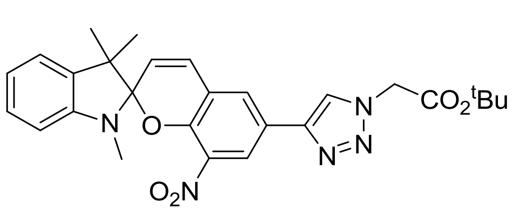
N
O
O2N
N
N
N
CO2tBu

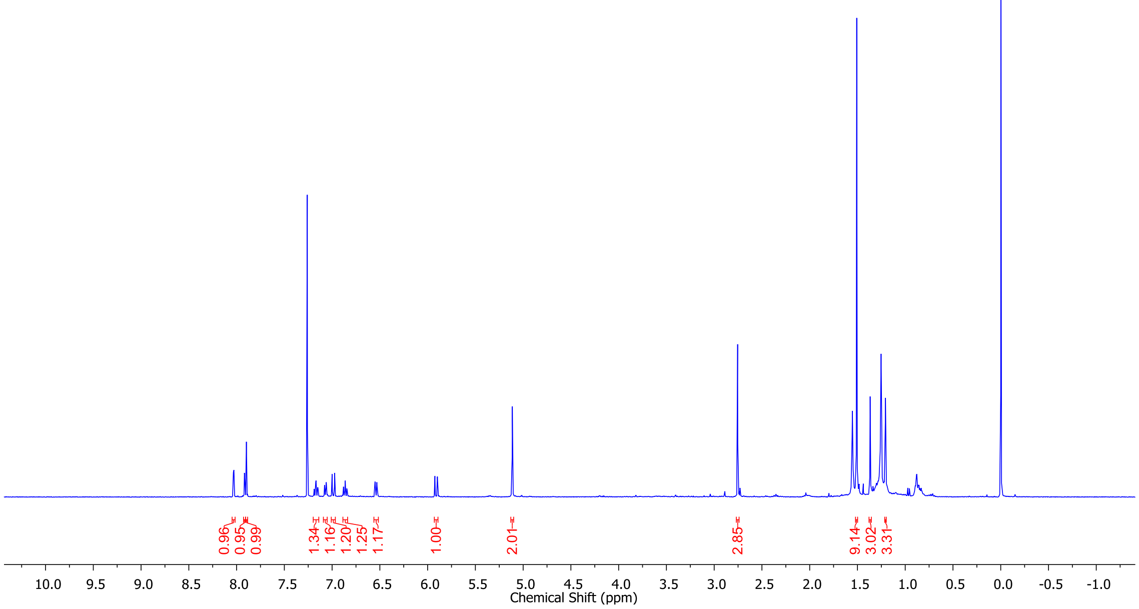
0.96
0.95
0.99
1.34
1.16
1.20
1.25
1.17
1.00
2.01
2.85
9.14
3.02
3.31
10.0 9.5 9.0 8.5 8.0 7.5 7.0 6.5 6.0 5.5 5.0 4.5 4.0 3.5 3.0 2.5 2.0 1.5 1.0 0.5 0.0 -0.5 -1.0
Chemical Shift (ppm)

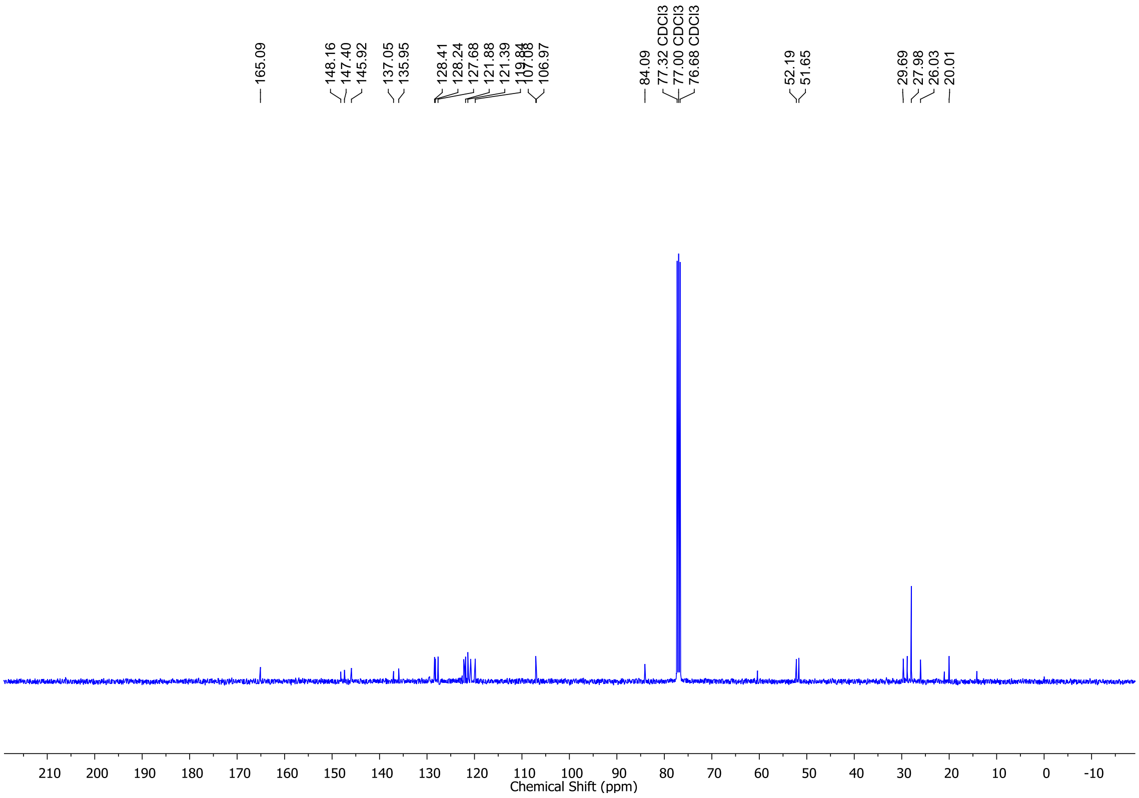
165.09
148.16
147.40
145.92
137.05
135.95
128.41
128.24
127.68
121.88
121.39
107.08
106.97
84.09
77.32 CDCl3
77.00 CDCl3
76.68 CDCl3
52.19
51.65
29.69
27.98
26.03
20.01
210 200 190 180 170 160 150 140 130 120 110 100 90 80 70 60 50 40 30 20 10 0 -10
Chemical Shift (ppm)

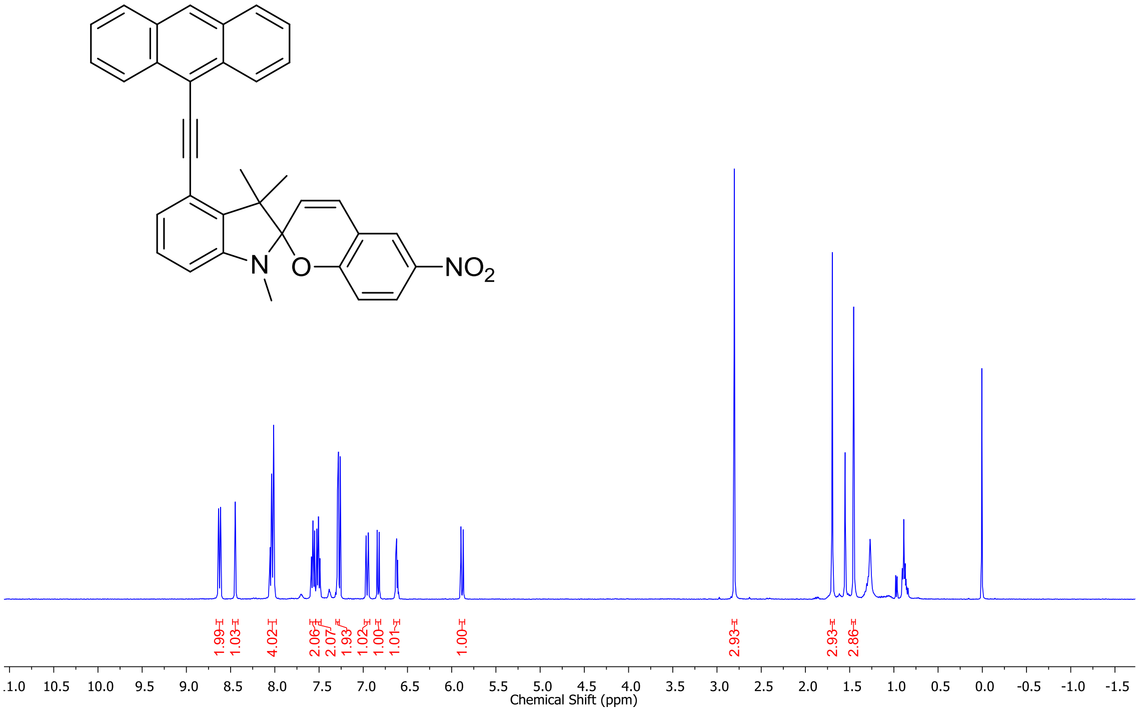

N
O
NO_2
1.99
1.03
4.02
2.06
2.07
1.93
1.02
1.00
1.01
1.00
2.93
2.93
2.86
Chemical Shift (ppm)

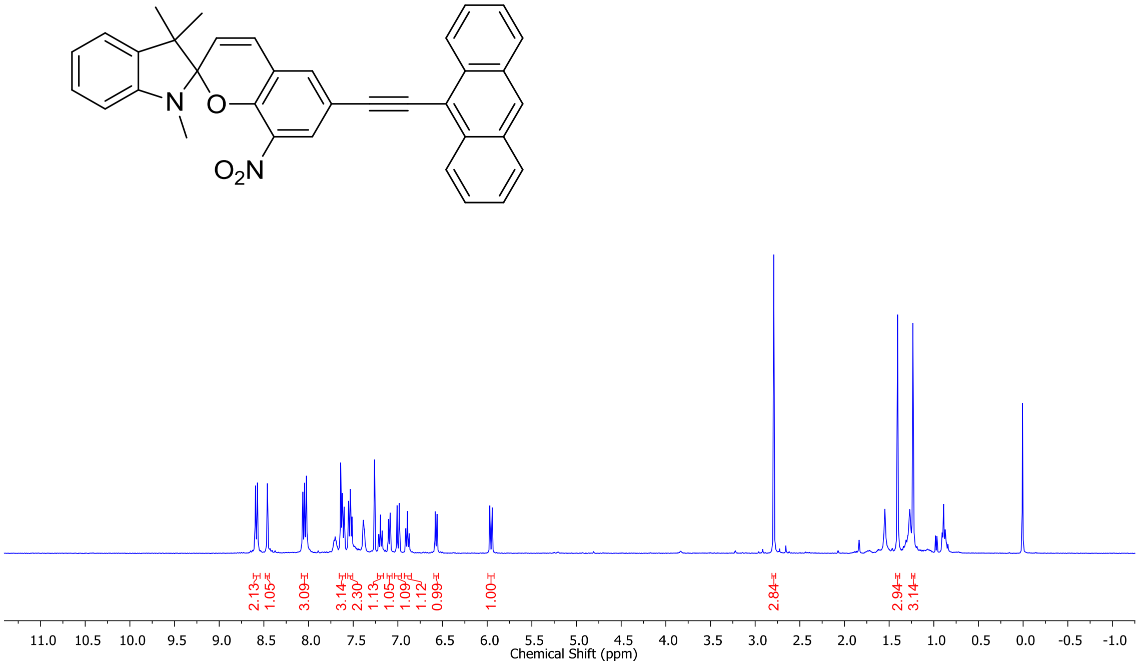

N
O
O_2N
2.13
1.05
3.09
3.14
2.30
1.13
1.05
1.09
1.12
0.99
1.00
2.84
2.94
3.14
Chemical Shift (ppm)

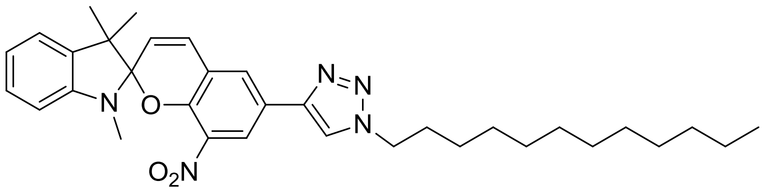
N
N
N
N
O
O2N

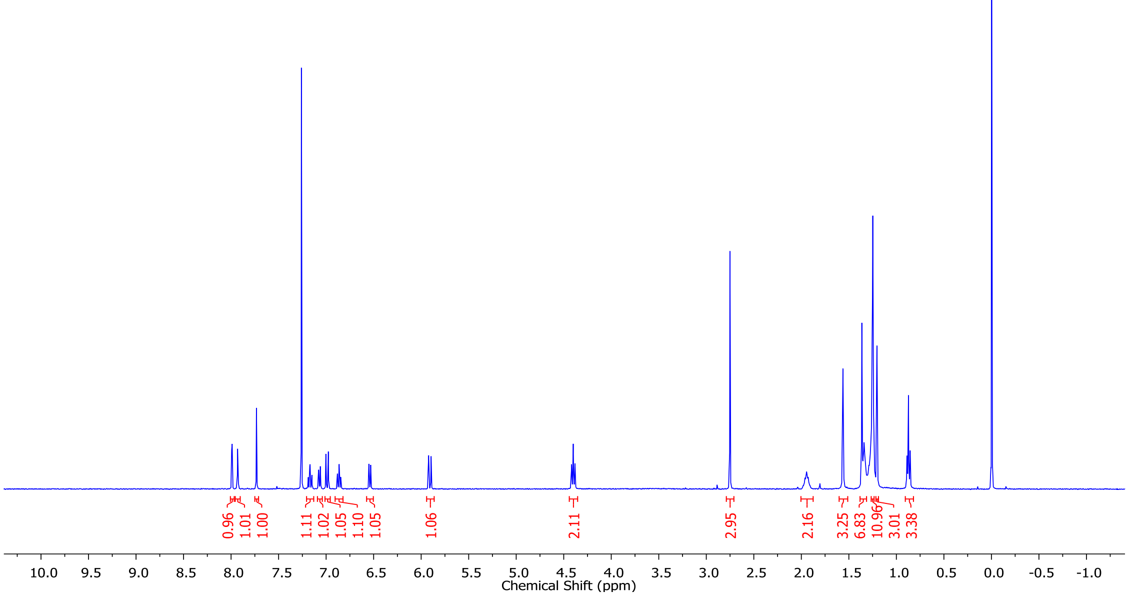
0.96
1.01
1.00
1.11
1.02
1.05
1.10
1.05
1.06
2.11
2.95
2.16
3.25
6.83
10.96
3.01
3.38
10.0 9.5 9.0 8.5 8.0 7.5 7.0 6.5 6.0 5.5 5.0 4.5 4.0 3.5 3.0 2.5 2.0 1.5 1.0 0.5 0.0 -0.5 -1.0
Chemical Shift (ppm)

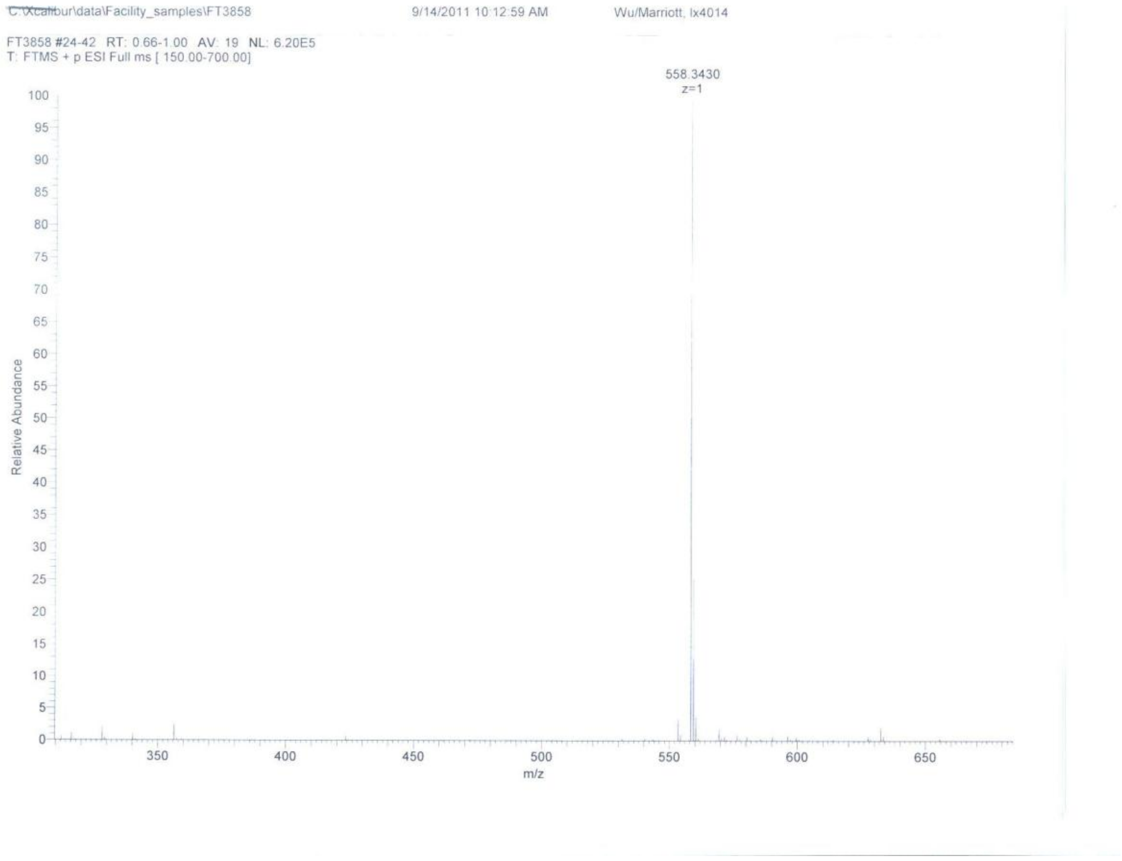
C:\Xcalibur\data\Facility_samples\FT3858
9/14/2011 10:12:59 AM
Wu/Marriott, lx4014
FT3858 #24-42 RT: 0.66-1.00 AV: 19 NL: 6.20E5
T: FTMS + p ESI Full ms [150.00-700.00]
558.3430
z=1
Relative Abundance
100
95
90
85
80
75
70
65
60
55
50
45
40
35
30
25
20
15
10
5
0
350
400
450
500
550
600
650
m/z
